# Long‐term and widespread changes in agricultural practices influence ring‐necked pheasant abundance in California

**DOI:** 10.1002/ece3.2675

**Published:** 2017-03-15

**Authors:** Peter S. Coates, Brianne E. Brussee, Kristy B. Howe, Joseph P. Fleskes, Ian A. Dwight, Daniel P. Connelly, Matt G. Meshriy, Scott C. Gardner

**Affiliations:** ^1^ U.S. Geological SurveyWestern Ecological Research CenterDixonCAUSA; ^2^ Nevada Natural Heritage ProgramCarson CityNVUSA; ^3^ California Department of Fish and WildlifeSacramentoCAUSA; ^4^ Pheasants ForeverSaint PaulMNUSA

**Keywords:** abundance, Annual Game Take Survey, Breeding Bird Survey, California, Christmas Bird Count, cropland, habitat loss, land use conversion, pesticide, *Phasianus colchicus*

## Abstract

Declines in bird populations in agricultural regions of North America and Europe have been attributed to agricultural industrialization, increases in use of agrochemical application, and increased predation related to habitat modification. Based on count data compiled from Breeding Bird Survey (BBS) from 1974 to 2012, Christmas Bird Count (CBC) collected from 1914 to 2013, and hunter data from Annual Game Take Survey (AGTS) for years 1948–2010, ring‐necked pheasants (*Phasianus colchicus*) in California have experienced substantial declines in agricultural environments. Using a modeling approach that integrates all three forms of survey data into a joint response abundance index, we found pheasant abundance was related to the amount of harvested and unharvested crop land, types of crops produced, amount of total pesticide applied, minimum temperature, precipitation, and numbers of avian competitors and predators. Specifically, major changes in agricultural practices over the last three decades were associated with declines in pheasant numbers and likely reflected widespread loss of habitat. For example, increases in cropland were associated with increased pheasant abundance during early years of study but this effect decreased through time, such that no association in recent years was evidenced. A post hoc analysis revealed that crops beneficial to pheasant abundance (e.g., barley) have declined substantially in recent decades and were replaced by less advantageous crops (e.g., nut trees). An additional analysis using a restricted data set (1990–2013) indicated recent negative impacts on pheasant numbers associated with land use practices were also associated with relatively high levels of pesticide application. Our results may provide valuable information for management policies aimed at reducing widespread declines in pheasant populations in California and may be applicable to other avian species within agricultural settings. Furthermore, this general analytical approach is not limited to pheasants and could be applied to other taxa for which multiple survey data sources exist.

## 1 Introduction

Avian diversity and abundance have recently experienced rapid declines in agricultural lands within North America (Dahlgren, 1988; Hiller, Taylor, Lusk, Powell, & Tyre, 2015), largely attributed to agricultural industrialization which predominantly includes declines in Conservation Reserve Program (CRP; Berner, 1988; Haroldson, Kimmel, Riggs, & Berner, 2006), removal of hedgerows, and the reduction in other uncultivated seminatural habitats adjacent to crop fields (i.e., “clean‐farming” practices; Chamberlain, Fuller, Bunce, Duckworth, & Shrubb, 2000; Benton, Vickery, & Wilson, 2003). These changes in agricultural practices have been linked to substantial losses of ecological heterogeneity at multiple spatiotemporal scales and widespread declines in biodiversity (Benton et al., 2003). However, other causal factors related to agriculture are also important, including crop type conversion and timing of cultivation (Glemnitz, Zander, & Stachow, 2015), increased agrochemical application (Mineau & Whiteside, 2006), and increased predation largely resulting from habitat changes (Evans, 2004).

Longitudinal studies using legacy survey data at relatively large spatial scales can help address how broad‐scale changes in agricultural lands have impacted birds that are generally associated with agricultural landscapes, referred to hereafter as farmland birds. Standardized count surveys have been conducted for decades in many agricultural settings and can provide a useful index for avian abundance to help inform decisions related to environmental management and policy (Stephens, Pettorelli, Barlow, Whittingham, & Cadotte, 2015). Studies are often limited to one form of survey design which usually has logistical and analytical limitations (Gregory, Gibbons, & Donald, 2004) and potential to mislead conservation and management decision processes. Given limitations in spatial and temporal data in most ecological studies, integrating multiple survey responses into a single analytical approach may improve predictive outputs and provide better proxies to inform management efforts. Thus, methodology to combine multiple responses into a single joint index allows access to more data across space and time and will likely prove beneficial to understanding the impacts of changes in agricultural lands on farmland bird abundance.

The ring‐necked pheasant (*Phasianus colchicus*, hereafter pheasant) is a common farmland bird species in California that was originally introduced from Asia, and populations have been successfully established since the early 1900s (Lever, 1987). In the relatively arid western United States, pheasants are traditionally associated with irrigated agriculture, as well as wetlands and open rangelands, where they feed on a variety of cereal grains, weed seeds, and invertebrates (Warner, Etter, Joselyn, & Ellis, 1984). Early establishment of pheasants in California was most successful in areas producing cereal grain crops with adjacent fencerows, headlands, wetlands, riparian, and other natural features, and the species became an economically important upland game bird (Hart, 1990; Lauckhart & McKean, 1956). However, current pheasant populations have decreased to historically low numbers throughout California (Sauer et al., 2014). In the case where pheasants may serve as a biomonitor species for broad environmental changes within agricultural ecosystems (Nielson et al., 2008), identifying factors that affect pheasant populations will likely provide insight regarding population dynamics of multiple avian species that are faced with similar changing environments.

To summarize declines in pheasant abundance over the course of nearly 100 years (1914–2013), we quantitatively combined three long‐term data sets based on standardized surveys, which were Annual Game Take Survey (AGTS; CDFW 2010), statewide Breeding Bird Survey (BBS; Sauer et al., 2014), and Christmas Bird Count (CBC; National Audubon Society 2014), into a joint pheasant abundance index. We employed a correlational study approach by modeling environmental factors that likely affect other wildlife in agricultural settings. Although a body of literature exists on addressing changes in biodiversity using composite diversity indices (Buckland, Magurran, Green, & Fewster, 2005), few studies have investigated factors influencing a single species of interest using data from multiple survey sources (Freeman, Noble, Newsom, & Baillie, 2007; Link & Sauer, 2007; Link, Sauer, & Niven, 2008). Specifically, we investigated relationships between agricultural land use, indices of avian predators and competitors, and climate trends. Using a restricted data set, we evaluated pesticide application for which data were only available since the 1990s. Lastly, we conducted a post hoc analysis to evaluate differences among specific crop types.

## 2 Methods

### 2.1 Study area

**Figure 1:**
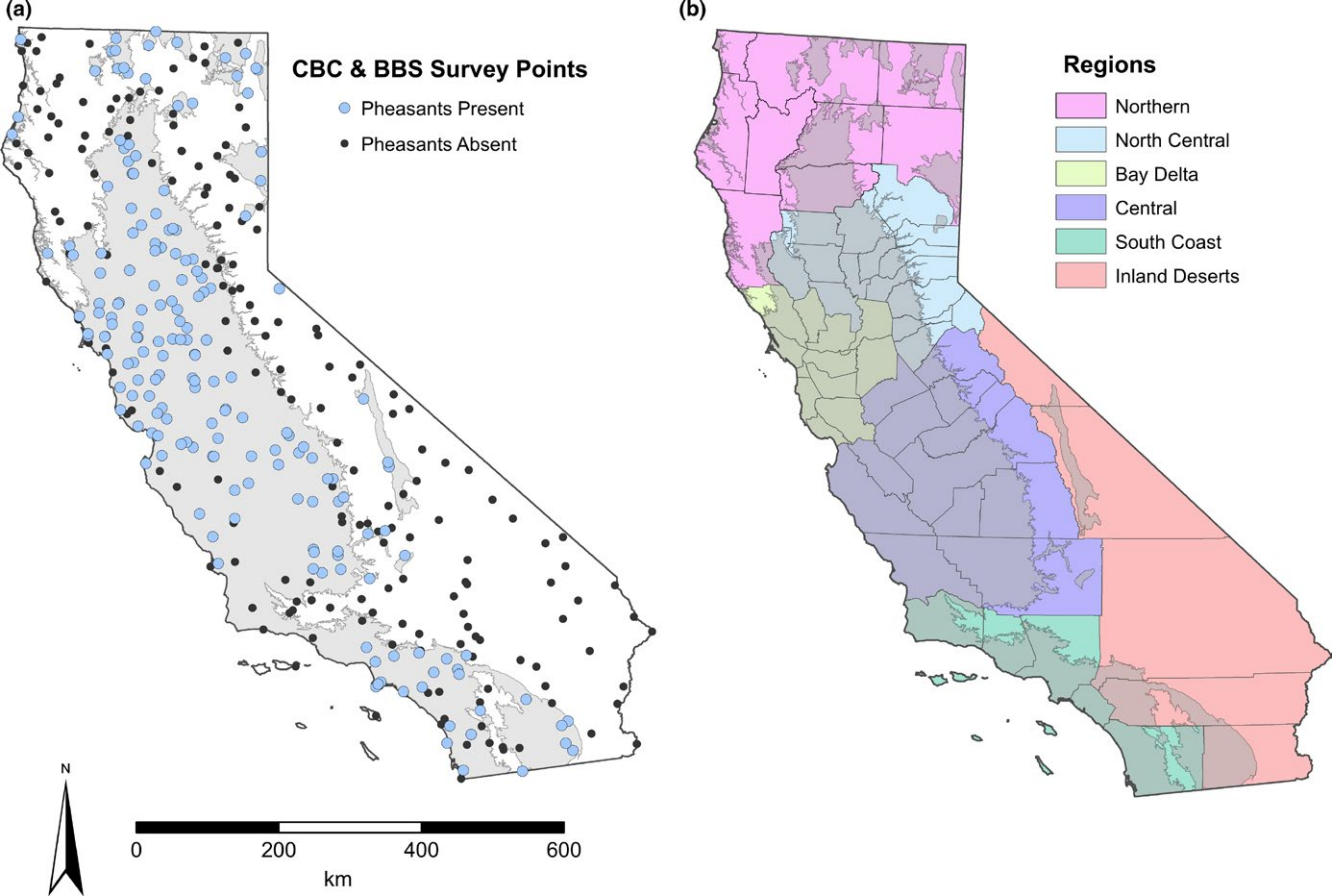
Maps depicting (a) ring‐necked pheasant (*Phasianus colchicus*) locations at Breeding Bird Survey (BBS) and Christmas Bird Count (CBC) surveys in California from 1914 to 2013, (b) California regions modified from California Department of Fish and Wildlife Areas. Gray boundary represents the study area delineation

The study area was delineated based on pheasant occurrence, elevation, and suitable land cover types within the state of California, USA (Figure 1). Specifically, using a geographical information system (GIS; ArcGIS 10.2, ESRI, Redlands, CA), we mapped all BBS routes conducted during 1968–2013 and CBC stations conducted during 1914–2013. Survey routes and stations in which pheasants were detected on greater than one occasion over the history of the surveys were used to create the study area boundary (Figure 1a). We excluded survey routes and stations located in land cover types (forests and deserts) and elevations (>1,500 m) that do not typically support pheasant populations (Hartman & Sheffer, 1971). Of the 260 BBS routes previously or currently surveyed in California, 70 routes were used to delineate the study boundary. Of the 232 CBC stations previously or currently surveyed in California, 115 stations were used to delineate the study boundary. We sought to carry out a hierarchical study design to make inferences among multiple spatial scales (i.e., region and statewide), allowing an investigation of regional variation in factors influencing pheasant abundance. Therefore, the study area was divided into the six state wildlife regions defined by the California Department of Fish and Wildlife (CDFW; Figure 1b). This delineation allowed us to conduct our data analyses across the entire state while accounting for variation between regions as well as allowing separate analyses for each individual wildlife region.

### 2.2 Statistical methods

We compiled data on pheasant abundance for each county in California in which pheasants have occurred based on BBS data from 1974 to 2012 (Sauer et al., 2014), CBC data collected from 1914 to 2013 (National Audubon Society 2014), and hunter harvest data from the AGTS for years 1948–2010 (CDFW 2010). Using methods detailed in Appendix S1, we combined data from the three different sources to create a “joint pheasant abundance index” for our response variable (see Appendix S1) and modeled the joint index as the response of multiple environmental factors (Table 1). We collected and compiled data on land use practices in California during 1949–2012 from Department of Agriculture census data (USDA 2014a), avian nest predators (corvids), predators of adult pheasants (raptors), pheasant competitors (wild turkeys) from BBS during 1974–2012 (Sauer et al., 2014), PRISM (Parameter‐elevation Regressions on Independent Slopes Model) climate data from 1913 to 2013 (Daly et al., 2008), county‐specific pesticide application data (total pounds applied annually) during 1990–2013 (California Department of Pesticide Regulation 2014), and crop acreage data from Department of Agriculture survey data (USDA 2014b). Detailed descriptions of all data sources and data compilation are located in Appendix S1.

**Table 1:** Predictor variables considered for a generalized linear mixed effects models of ring‐necked pheasant (*Phasianus colchicus*) abundance in California

Variable	Years available	Source^a^	Units
Harvested cropland	1949–2012	USDA census data	% area
Cropland as pasture	1949–2012	USDA census data	% area
Unharvested cropland	1949–2012	USDA census data	% area
CRP	1949–2012	USDA census data	% area
Corvid abundance	1969–2013	BBS	Birds counted
Raptor abundance	1969–2013	BBS	Birds counted
Turkey abundance	1969–2013	BBS	Birds counted
Minimum temperature (Breeding)	1913–2013	PRISM	°C
Minimum temperature (Brood rearing)	1913–2013	PRISM	°C
Minimum temperature (Winter)	1913–2013	PRISM	°C
Precipitation (Breeding)	1913–2013	PRISM	mm
Precipitation (Brood rearing)	1913–2013	PRISM	mm
Precipitation (Winter)	1913–2013	PRISM	mm
Barley	1954–2013	USDA survey data	% area
Sugar beets	1975–2012	USDA survey data	% area
Nut Trees	1980–2012	USDA survey data	% area
Winter wheat	1945–2013	USDA survey data	% area
Sorghum	1972–2008	USDA survey data	% area
Veg seed	1980–2012	USDA survey data	% area
Rice	1953–2012	USDA survey data	% area
Cotton	1960–2012	USDA survey data	% area
Grapes	1980–2012	USDA survey data	% area
Hay	1980–2012	USDA survey data	% area
Corn	1959–2013	USDA survey data	% area
Wheat	1974–2013	USDA survey data	% area
Oats	1974–2011	USDA survey data	% area
Fruit trees	1980–2012	USDA survey data	% area
Total pesticides	1990–2012	CDPR	kg/ha

a USDA = U.S. Department of Agriculture; BBS = Breeding Bird Survey; CDPR = California Department of Pesticide Regulation.

For all analyses, we developed generalized linear mixed effects models (Zuur, Ieno, Walker, Saveliev, & Smith, 2009), whereby we fit environmental factors as fixed effects and specified random effects of county nested within region to account for repeated measures (Faraway, 2006). All analyses were conducted at the county–year level. An additional factor variable “survey type” (i.e., AGTS, BBS, and CBC) was included as a fixed effect (i.e., categorical variable) in every model to account for differences in intercepts among the three data sources. A model with only survey type and random effects served as a “baseline model” to compare fit with other models that consisted of additional fixed effects. A null model was also fit which consists of random effects only (without survey type). All variables were modeled as 1‐year lag effects because pheasant abundance in a given year is related to productivity and factors influencing productivity (e.g., weather, predators, and land use) in the previous year (Benton, Bryant, Cole, & Crick, 2002).

Prior to developing additive‐effect models of pheasant abundance, we evaluated the efficacy of the joint pheasant abundance index in relation to individual independent survey type response (BBS, CBC, and AGTS; Table S1). The primary purpose of this assessment was to investigate whether or not the joint index was more reliable in estimating effects of environmental factors influencing pheasant abundance than any single‐survey abundance index. To assess each response index, we derived parameter estimates (i.e., slope coefficients) and their 95% confidence intervals (CI) from single‐variable models using data sets restricted for each survey response index, as well as for the joint index. The joint index consisted of the most amount of data through space and time, and parameter estimates from this index appeared to be less prone to the biases associated with any single index. Therefore, further modeling was evaluated using the joint index as a response variable rather than any one type of survey.

As an initial variable reduction technique prior to developing more complex models, we evaluated groups of models (e.g., climate effects) individually with unique data sets to identify the most parsimonious model within each group (Table S2) and no comparisons were made between variables from different groups. Data sets varied in time spans (Table 1), which allowed for full use of the available data. For example, some variables dated back nearly 100 years, such as CBC and PRISM (climate data). For each restricted data set, the response variable was standardized using procedures described in Appendix S1. We included interactions between the amount of cropland (harvested and unharvested) and year (e.g., harvested cropland × year) to investigate the influence of agricultural land use changes through time. To assess model fit and evidence between models within a group, we used Akaike information criterion (AIC) corrected for sample size (*c*), computed differences in AIC_c_ values (Δ), and calculated Akaike weights (*w*_*i*_) and evidence ratios (Anderson, 2008). Models that consisted of AIC_c_ scores less than two units below the baseline model (i.e., “survey type” as fixed effect) and below the null model (i.e., random effects only) were supported by the data, and variables from those models were carried forward from this initial variable reduction technique to the modeling analysis. For the land use group, the higher order effects that met this same criterion were carried forward. All models were fit using lme4 package (Bates & Maechler, 2010) in Program R, and the MuMIn package (Barton, 2014) was used to calculate conditional *R* ^2^ to further assess model fit.

In a first analysis, we developed models of additive effects using variables that were carried forward from the variable reduction process. Data sets were restricted (1974–2013) to omit all lines with missing data for any given predictor, and no more than four effects were allowed in each model to prevent overfitting (maximum number of estimated parameters was 8). The purpose of this analysis was to investigate the effects of changes in agricultural land use over time (i.e., interactions), while accounting for other environmental factors, on pheasant abundance using data that date back nearly 40 years. We excluded all models that had covariates with evidence of correlation among predictors (*R* ≥ |.65|). Relative importance of covariates using adjusted (Adj.) probabilities and evidence ratios were calculated for each of the covariates within the analysis, accounting for unequal representation of covariates across the model set following procedures described in Appendix S2. To investigate variation in effects across the landscape, we carried out the same modeling approach separately for each region and calculated variable importance. Interpretations of parameter estimates were calculated based on the joint pheasant abundance index which represented relative abundance.

To investigate the effects of pesticides, we conducted a separate analysis using data further restricted to later years of study (1990–2013) when pesticide information was available. The purpose of this second analysis was to investigate more complex relationships between pesticides, cropland, and years on pheasant abundance using data that date back to 1990s. We examined pesticide application as an interaction with amount of cropland (harvested and unharvested) and year (three‐way interaction; e.g., harvested cropland × pesticide × year). We evaluated this three‐way interaction for two primary reasons. First, we hypothesized that the effects of pesticide varied across levels of cropland (two‐way interaction; e.g., harvested cropland × pesticide) and those effects varied through time (three‐way interaction). Additionally, this interaction accounted for inherent correlation between amount of cropland and pesticide application (i.e., pesticide use on croplands was greater than on other lands). We developed a model set that included this higher order interaction as an additive term with other variables supported by the data that were identified from the variable reduction procedure. Similar diagnostic tests for correlation among predictors and model evaluation techniques using AIC_c_ criterion were used in this analysis, as described previously. For interpretation purposes of the three‐way interaction, we illustrate the interaction by assigning low pesticide amounts as the bottom 10th percentile (≤0.1 kg ha^−1^ yr^−1^) and high pesticide as the top 10th percentile (≥7.7 kg ha^−1^ yr^−1^) and assigned year as early (1991–2000) and late (2001–2013) classes, even though both pesticide and year were treated as continuous variables in the model.

For a post hoc crop type analysis, we again restricted the data to all crop types available (1986–1989 and 2002–2009) so that we could compare support across all crop types (Table 1) as single‐variable models and evaluated model evidence using similar AIC_c_ criterion.

## 3 Results

### 3.1 Modeling pheasant abundance index

**Figure 2:**
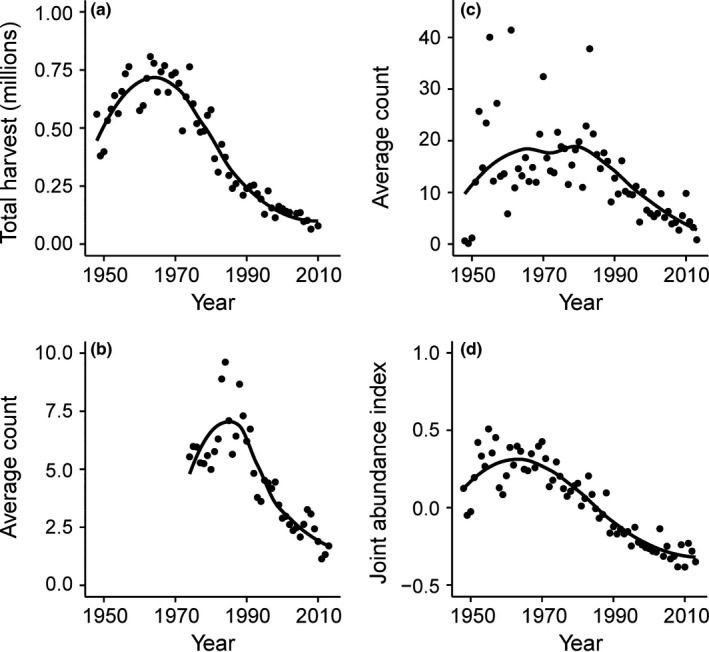
Statewide annual ring‐necked pheasant (*Phasianus colchicus*) (a) total harvest, (b) average Breeding Bird Survey count, (c) average Christmas Bird Count, and (d) the joint pheasant abundance index in California, USA. For illustrative purposes, we depict data from 1948 to 2013. Solid line represents LOESS curve

Pheasant populations have declined substantially over the past 25 years based on abundance indices from all three survey techniques (Figure 2). A detailed description of statewide and regional trends in pheasant abundance indices and predictor variables is provided in Appendix S3. In comparing each survey response (BBS, CBC, and AGTS) with the joint index on single‐variable effects, we found the strongest evidence of support from results of the joint index was observed for those variables that garnered strong evidence across all three indices independently (i.e., single‐survey response, Table S1). For example, we found substantial support (95% CIs of the parameter estimates did not overlap zero) for the single variables unharvested cropland, corvid abundance, raptor abundance, turkey abundance, minimum temperature (breeding, brood rearing, and winter), and precipitation (breeding and brood rearing), as well as support for the interaction between harvested cropland and year. In comparison with the independent single‐survey responses (BBS, CBC, and AGTS), these variables were all supported by the data. We found lack of support (95% CIs overlap zero) for variables harvested cropland, precipitation (winter), and the interaction between unharvested cropland and year. These variables consisted of disagreement of support across the single‐survey types. We found that disagreement across single indices resulted in uncertainty in the joint index parameter estimate (e.g., 95% CI of parameter estimate overlapped zero), even in those cases which one particular index was strongly supported (e.g., interaction between unharvested cropland and year). For those variables with evidence of support (95% CI of parameter estimate does not overlap zero) using the joint index, we found disagreement in the uncertainty in the effects across indices, but rarely did the direction of the parameter (positive vs. negative) disagree. These results provided rationale to carry forward the full analysis using the joint abundance index.

**Figure 3:**
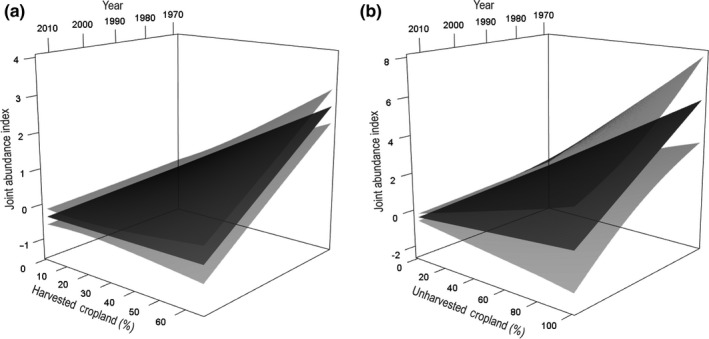
Effect of an interaction between (a) percent harvested cropland and year, and (b) percent unharvested cropland and year on the joint ring‐necked pheasant (*Phasianus colchicus*) abundance index from Breeding Bird Survey, Christmas Bird Count, and Annual Game Take Survey data in California, USA, ranging from 1914 to 2013. Predictions were derived from the most parsimonious model while all other effects were held at their means. Note differences in *y*‐axis scales. Dark gray plane represents mean estimate, whereas light gray represents 95% confidence intervals

Results of the variable reduction procedure for predictor variables carried forward to the additive modeling analysis using the joint index are provided in Appendix S4 and Table S2. Within the analysis, we ultimately ran 292 models, excluding models with variables with evidence of correlation ([*R* ≥ |.65|]; see Tables S3 and S4) using 3,231 county/year samples to assess factors influencing pheasant abundance. The top model relating pheasant abundance to environmental variables included two interactions of harvested cropland × year and unharvested cropland × year (Table S4). This model indicated an increase in pheasant abundance with increased cropland (harvested and unharvested). However, this effect varied through time. For example, the positive effect of harvested cropland on pheasant abundance was most influential during early years of study and decreased through time, such that in recent years, no general association was evidenced between pheasant abundance and harvested cropland (Figure 3a). A similar pattern was observed with unharvested cropland (Figure 3b). However, the unharvested interaction showed substantially less support from the data than the harvested interaction. In comparing model evidence between two models with each consisting of an interaction, the harvested × year interaction was 174.2 AIC_*c*_ units lower (Table S4). Furthermore, 95% prediction intervals of the unharvested × year were relatively greater (Figure 3a,b).

In evaluating evidence of all of variables within the final model set, higher order interactions that included harvested cropland × year and unharvested cropland × year were the best predictors of pheasant abundance (Adj. probability = 1.00; Table 2). After accounting for the variation described by cropland × year, we found some evidence that minimum temperature affected pheasant abundance during the breeding period (Adj. ER = 1.01). However, 95% CIs of the parameter estimate for temperature overlapped zero in each of the top models.

**Table 2:** Relative importance of covariates based on adjusted (Adj.) probability and Adj. evidence ratio (ER) derived from ring‐necked pheasant (*Phasianus colchicus*) abundance models in California

Rank	Covariate	Adj. probability	Adj.
1	Harvested cropland × year	1.00	>100
2	Unharvested cropland × year	1.00	>100
3	Minimum temperature (Breeding)	.50	1.01
4	Precipitation (Brood rearing)	.40	0.67
5	Minimum temperature (Winter)	.33	0.49
6	Precipitation (Breeding)	.33	0.49
7	Minimum temperature (Brood rearing)	.33	0.48
8	Turkey abundance	.24	0.32
9	Corvid abundance	.24	0.32
10	Raptor abundance	.24	0.31

### 3.2 Regional results

For every region, we found evidence for a land use covariate (Table 3). For the Northern, North Central, Bay Delta, Central, and Inland Desert regions, the interaction between harvested cropland and year garnered substantial support from the data. The interaction including unharvested cropland and year was also supported by the data in the Northern, North Central, Central, and Inland Desert regions (Adj. ER > 1.00) and garnered the most support in the South Coast region. Corvid abundance was the most influential variable to pheasant abundance in the North Central Region (Adj. ER > 100.00), second most influential in the Bay Delta, and third most influential in the South Coast region (Adj. probability = .60 and 0.64, respectively). In the Central region, precipitation during the brood rearing period was the second most important variable (Adj. probability = .98) after land use practices. Additionally, the climate variable describing minimum temperature during the breeding period was influential in the North Central, Central, and South Coast regions, and minimum temperature during the brood rearing period was influential in the Inland Desert Regions (Adj. ER > 1.00). Detailed model results for each of the six regions are available in Supplemental Information Tables S5–S16.

**Table 3:** Relative importance of covariates based on adjusted (Adj.) probability and Adj. evidence ratio (ER) derived from ring‐necked pheasant (*Phasianus colchicus*) abundance models in six regions of California, 1914–2013

Region	Rank	Covariate	Adj. probability	Adj. ER
Northern	1	Harvested cropland × year	1.00	>100
	2	Unharvested cropland × year	1.00	>100
	3	Minimum temperature (Breeding)	.47	0.89
	4	Minimum temperature (Winter)	.34	0.52
	5	Minimum temperature (Brood rearing)	.34	0.52
North central	1	Corvid abundance	1.00	>100
	2	Harvested cropland × year	1.00	>100
	3	Unharvested cropland × year	.92	11.21
	4	Minimum temperature (Breeding)	.61	1.59
	5	Minimum temperature (Winter)	.37	0.59
	6	Minimum temperature (Brood rearing)	.31	0.44
	7	Precipitation (Brood rearing)	.13	0.15
Bay delta	1	Harvested cropland × year	1.00	>100
	2	Corvid abundance	.60	1.51
	3	Minimum temperature (Winter)	.45	0.81
	4	Minimum temperature (Brood rearing)	.43	0.74
	5	Raptor abundance	.32	0.47
	6	Minimum temperature (Breeding)	.30	0.43
	7	Turkey abundance	.29	0.42
	8	Unharvested cropland × year	.15	0.18
Central	1	Harvested cropland × year	1.00	>100
	2	Precipitation (Brood rearing)	.98	63.63
	3	Minimum temperature (Breeding)	.88	7.50
	4	Unharvested cropland × year	.54	1.19
	5	Minimum temperature (Winter)	.24	0.32
	6	Raptor abundance	.19	0.24
	7	Corvid abundance	.15	0.18
	8	Minimum temperature (Brood rearing)	.09	0.10
South coast	1	Unharvested cropland × year	.68	2.12
	2	Minimum temperature (Breeding)	.68	2.09
	3	Corvid abundance	.64	1.78
	4	Harvested cropland × year	.32	0.47
	5	Minimum temperature (Brood rearing)	.28	0.39
	6	Minimum temperature (Winter)	.27	0.37
Inland deserts	1	Harvested cropland × year	1.00	>100
	2	Unharvested cropland × year	.98	56.10
	3	Minimum temperature (Brood rearing)	.93	12.34
	4	Precipitation (Winter)	.14	0.16
	5	Minimum temperature (Breeding)	.10	0.11

### 3.3 Pesticide analysis

**Figure 4:**
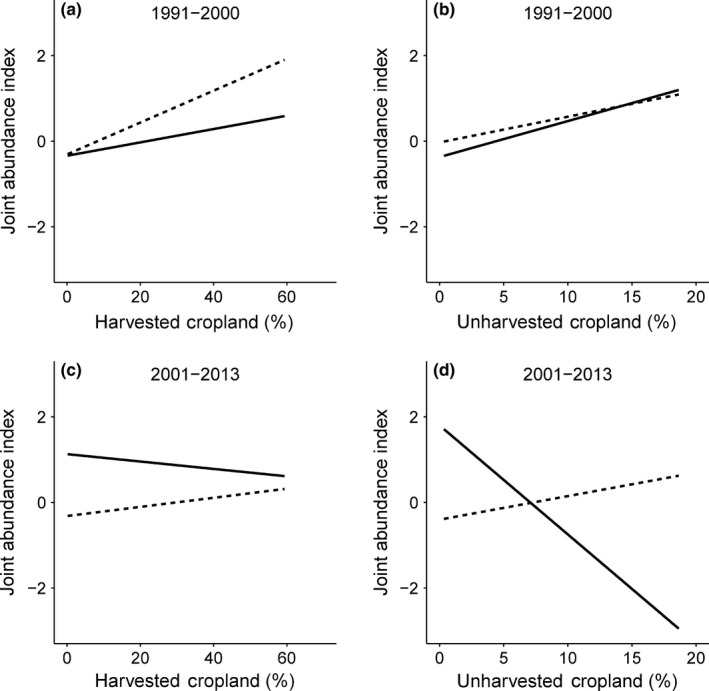
Effect of the interaction of (a) harvested cropland with pesticide and year from 1991 to 2000, (b) unharvested cropland with pesticide and year from 1991 to 2000, (c) harvested cropland with pesticide and year from 2001 to 2013, and (d) unharvested cropland with pesticide and year from 2001 to 2013 on the joint ring‐necked pheasant (*Phasianus colchicus*) abundance index from Breeding Bird Survey, Christmas Bird Count, and Annual Game Take Survey data in California, USA. Solid lines represent high levels of pesticides, and dotted lines represent low levels. Predictions were derived from the most parsimonious model while all other effects were held at their means

The pesticide analysis model set consisted of 292 models (see Table S17) using 1,600 county/year samples. The top model in the pesticide analysis included the interaction of harvested cropland with pesticide and year, and the interaction of unharvested cropland with pesticide and year. This model predicted that across all years at low pesticide levels, there was a positive influence of harvested and unharvested cropland on pheasant abundance (Figure 4a–d), whereas high levels of pesticide application resulted in a reduced impact of both types of cropland practices in later years, particularly in unharvested areas (Figure 4d). The two interactions of cropland (harvested and unharvested) with pesticides and year were the most influential covariates within the final model set (Adj. ER = >100.00; Table 4).

**Table 4:** Relative importance of covariates based on adjusted (Adj.) probability and Adj. evidence ratio (ER) derived from ring‐necked pheasant (*Phasianus colchicus*) abundance models from a restricted data set that included pesticide effects in California

Rank	Covariate	Adj. probability	Adj. ER
1	Harvested cropland × Total pesticide × year	1.00	>100
2	Unharvested cropland × Total pesticide × year	1.00	>100
3	Corvid abundance	.46	0.85
4	Minimum temperature (Breeding)	.39	0.63
5	Minimum temperature (Brood rearing)	.38	0.61
6	Precipitation (Brood rearing)	.33	0.48
7	Minimum temperature (Winter)	.33	0.48
8	Precipitation (Breeding)	.29	0.40
9	Raptor abundance	.24	0.31
10	Turkey abundance	.23	0.29

### 3.4 Post hoc crop type analysis

**Figure 5:**
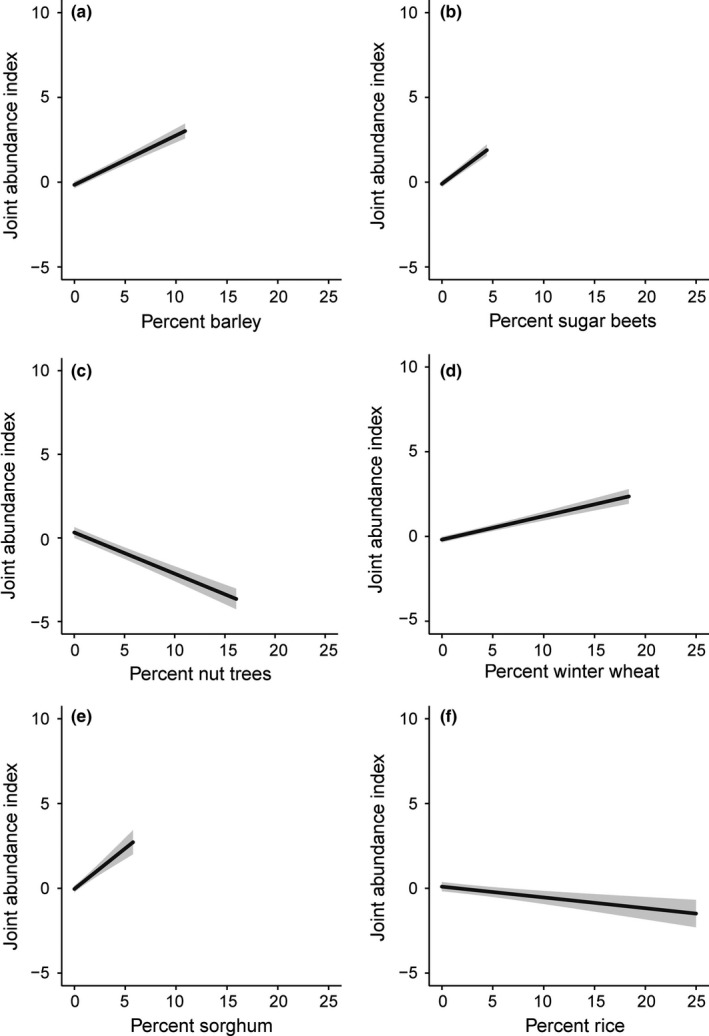
Effect of (a) percent barley, (b) percent sugar beets, (c) percent nut trees, (d) percent winter wheat, (e) percent sorghum, and (f) percent rice on the joint ring‐necked pheasant (*Phasianus colchicus*) abundance index from Breeding Bird Survey, Christmas Bird Count, and Annual Game Take Survey data in California, USA, ranging from 1914 to 2013. Shaded area represents 95% confidence intervals

We restricted data set to 2,100 samples (county/year) to evaluate how different crop types affect pheasant abundance and found strong support for multiple crop types (Table 5). For example, pheasant abundance was positively affected by the amount of barley (*w* = 1.00), sugar beets, winter wheat, sorghum (Figure 5), as well as vegetable seed crops, cotton, and corn (Table 5). We found the joint pheasant abundance index increased by 29.3% (95% CI: 25.5%–33.5%), 44.9% (95% CI: 38.8%–51.4%), 13.9% (95% CI: 11.3%–16.3%), 47.9% (95% CI: 35.8%–60.3%), and 33.8% (95% CI: 22.5%–44.4%) with a 1% increase in barley (Figure 5a), sugar beets (Figure 5b), winter wheat (Figure 5d), sorghum (Figure 5e), and vegetable seed, respectively. Trees (specifically, nut tree), rice, and grape production resulted in negative effects on pheasant abundance (Table 5). The joint pheasant abundance index decreased by 24.7% (95% CI: 20.8%–28.5%), 6.4% (95% CI: 3.1%–9.9%), and 10.3% (95% CI: 5.8%–14.2%) with a 1% increase in nut trees (Figure 5c), rice (Figure 5f), and grapes, respectively. We found no evidence for effects of oats, hay, wheat, or fruit trees on the joint pheasant abundance index (Table 5). Full results for the crop type analysis are provided in Table S18.

**Table 5:** Estimated parameter estimates (β) and 95% confidence intervals of post hoc crop type analysis using generalized linear mixed effects models on a joint index of ring‐necked pheasant (*Phasianus colchicus*) abundance in California

Model covariate	β (95% CI)
Barley	29.3 (25.5 to 33.5)
Sugar beets	44.9 (38.8 to 51.4)
Nut trees	−24.7 (−28.5 to −20.8)
Winter wheat	13.9 (11.3 to 16.3)
Sorghum	47.9 (35.8 to 60.3)
Vegetable seed	33.8 (22.5 to 44.4)
Cotton	5.0 (3.0 to 7.1)
Grapes	−10.3 (−14.2 to −5.8)
Rice	−6.4 (−9.9 to −3.1)
Corn	3.7 (0.0 to 6.8)
Oats	4.4 (−1.0 to 9.8)
Hay	−1.7 (−3.8 to 0.5)
Wheat	−4.4 (−11.4 to 2.7)
Fruit trees	0.5 (−6.0 to 7.5)

## 4 Discussion

Although declines in pheasant populations have been attributed to changes in farm management practices elsewhere (Chamberlain et al., 2000; Warner et al., 1984), to our knowledge, this study represents the first analysis of integrating multiple types of legacy survey data sets (BBS, CBC, and AGTS) to identify long‐term predictable patterns in the decline of a farmland species in relation to cropland practices interacting with other environmental drivers across a broad geographical region. Despite minimal changes in the amount of area used for crop production collectively across the study regions during 1945–2012 (see Appendix S3), changes were often drastic among different regions which corresponded to changes in pheasant abundance. For example, land used for farming became increasingly concentrated toward the Central and San Joaquin valleys as land in other regions became developed for nonfarm purposes (e.g., urbanization). As such, habitat value of agricultural lands for pheasants and other wildlife decreased as land idling decreased, farming intensity increased, and crops providing nesting or winter habitat decreased (Grove, Buhler, Henny, & Drew, 2001; Warner et al., 1984).

Similar to agricultural habitats in Europe (Robinson & Sutherland, 2002), small, diversified farms in California's Central Valley have given way to large, monoculture production fields (Mitchell et al., 2001). Such consolidation of farm units, as well as simplified crop rotations, has led to large, contiguous tracks of land under similar management systems at any given time (Robinson & Sutherland, 2002). Like most farmland birds, pheasants seasonally select a mosaic of farmed and natural habitats for food and shelter, often moving between a variety of crop types as fields mature and are harvested (Ramey, Bourassa, & Furuta, 2006). Most row crop acreage throughout California's Central Valley now produces only one crop per year, leaving large acreages of bare ground between crops, particularly during winter months from October through March (Mitchell et al., 2003). Furthermore, postharvest treatments such as increased levels of mechanization that reduce stubble height, herbicide application to stubble (Rodgers, 2002), as well as increased field flooding of harvested rice fields in response to phased reduction in rice straw burning (Connelly‐Areiras‐Chandler Rice Straw Burning Reduction, Assembly Bill 1378, Ch. 787, 1991), have reduced the value of harvested cropland for pheasants. Adult hen pheasants inhabiting intensively farmed habitats in northern California were shown to have lower body mass and shorter tarsal length than those inhabiting areas that were not intensively farmed, suggesting that pheasants in intensively farmed habitats may be nutritionally stressed (Grove et al., 2001).

The concentration of agriculture in the Central and San Joaquin valleys of California has also resulted in a significant loss of unharvested pastoral lands. For example, California has experienced loss of CRP land across the state, a land cover type positively associated with pheasant abundance across much of their range in North America (Nielson et al., 2008). Loss of CRP, coupled with reduction in other fallowed fields and cropland used as pasture, has resulted in a shrinking island of suitable refugia inhabited by populations of pheasants and other farmland species. Furthermore, size and types of crops surrounding unharvested cropland undoubtedly influence the value of remaining patches of noncropped land, whereby patches of suitable habitat surrounded by large, low crop diversity landscapes likely become population sinks for farmland bird species (Clark, Schmitz, & Bogenschutz, 1999; Schmitz & Clark, 1999; Wilson, Evans, Browne, & King, 1997).

Our post hoc analysis indicated that the value of agricultural lands to pheasants varies considerably depending on crop type. For example, conversion of barley to rice fields has been the strongest driver of pheasant habitat loss in the North Central region of California. Rice is generally avoided by nesting pheasants because rice fields are flooded for much of the growing season (Ramey et al., 2006). Many harvested rice fields that once provided pheasants with winter cover and foraging habitat have experienced increased rates of postharvest flooding for straw decomposition since the 1980s (Central Valley Joint Venture 2006; Fleskes, Perry, Petrik, Spell, & Reid, 2005). Conversion of cereal grain and row crops to nut orchards also has had negative effects on pheasant abundance in the Central Valley, and as little as 15% tree cover can severely limit pheasant populations (Jorgensen, Powell, Lusk, Bishop, & Fontaine, 2014). Nut tree orchards are typically devoid of any vegetative understory, thereby providing little to no cover or food. They may also alter predator–prey relationships by increasing perch and nesting substrates of avian predators as well as provide adequate habitat for turkey populations. Our analysis also revealed loss of sugar beets and sorghum has contributed to declines, crops which provide structural nesting cover (Glemnitz et al., 2015) and easily metabolized high‐energy food (Douglas, Sullivan, Bond, & Struwe, 1990), respectively.

Although land use changes garnered the most widespread statistical support, our regional analyses supported effects that varied spatially, such as climatic conditions. The temperature and amount of precipitation varied across regions of the state, and we found regional variation in the relative influence of these climatic variables (see Table 5). In general, drier and warmer areas of the state reflected stronger relationships with precipitation and temperature, whereby pheasant abundance increased in regions of relatively higher precipitation and lower temperatures. For example, minimum temperature during breeding, brood rearing, and winter influenced pheasant abundance throughout most of California, with exception of the Bay Delta region. Precipitation was also important during brood rearing period, particularly in the relatively dry central region. Water availability is influenced by temperature and precipitation and has been shown to be an important component of pheasant habitat (Johnsgard, 1999), reducing negative effects of drought conditions (Santilli & Bagliacca, 2008) and providing shrubby vegetation for winter, nesting, and escape cover, as well as an abundance of invertebrates (Smith, Stewart, & Gates, 1999). Low precipitation during brood rearing also decreases insects necessary to sustain chicks and juveniles (Hill, 1985; Messick, Bizeau, Benson, & Mullins, 1974). As a whole, California tends to be more arid than other areas where pheasant occur in the United States, so the relationships identified here might not exist in wetter and cooler regions of their range.

The effects that varied substantially across regions were those related to competitors and predators, receiving relatively less support than other variables but nonetheless substantiated by data. The strongest of such effects was corvid abundance, notably important in the North Central, Bay Delta, and South Coast regions. Corvid populations have significantly increased across western United States (Sauer et al., 2014) in response to ample anthropogenic resources (Boarman, Patten, Camp, & Collis, 2006; Marzluff, McGowan, Donnelly, & Knight, 2001). In California, corvid populations have increased most dramatically along coastal areas. Increases in raven numbers have been shown to negatively impact nesting success of other Galliformes (Coates & Delehanty, 2010). Raptor abundance also garnered support from these data, and predation by raptors on adult pheasants can be significant in some areas (Kenward, Marcström, & Karlbom, 1981) including northern California (Grove et al., 2001). However, negative effects related to increased avian predators (i.e., ravens and raptors) are likely exacerbated by lack of adequate cover remaining in intensively farmed cropland, low‐quality environments in which populations are more sensitive to predation (Evans, 2004). Interestingly, our results indicated a negative relationship between pheasant and wild turkey, another non‐native game bird species to California. This could be an indication that the two species respond differently to the reported land use changes, or could indicate a form of interspecific competition. To our knowledge, competition between these species has not been reported previously and warrants well‐designed experimental study.

Analysis from our restricted data set (1990–2013) revealed evidence of a three‐way interaction between cropland, pesticide uses, and years. Specifically, in the past decade, the effect of cropland, especially unharvested, was influenced by the amount of pesticide application. However, this effect was not supported in earlier years (i.e., 1990s). Effects of pesticide application in unharvested cropland was likely most influential in our study because these areas were more suitable for pheasant populations. For instance, changes in harvested crop types (e.g., barley to rice) have appeared to reduce pheasant habitat, perhaps increasing their dependence of unharvested cropland in recent decades. Furthermore, rice is primarily flooded during pesticide application and therefore of limited use to pheasants during that time, despite rice fields posing one of the highest cumulative risk of death to bird species as a result of high application rates of pesticides (Mineau & Whiteside, 2006). Pesticides applied for agricultural or human health (i.e., mosquito abatement) purposes have been shown to reduce carrying capacity of farmland birds directly through lethal and sublethal effects (Mineau & Whiteside, 2006; Ramey & Sterner, 1995), and indirectly by reducing cover quality and invertebrate food for chicks and adults (Grove et al., 2001; Messick et al., 1974). The use of insecticides such as organophosphates and neonicotinoids is pervasive in both agricultural and residential areas across California. Organophosphates affect functioning of nervous system bodies of insects and other organisms (Reigart & Roberts, 1999), and systemic insecticides such as neonicotinoids are absorbed by plants and transferred through the vascular system, making plants toxic to species that forage on them (Blacquière, Smagghe, van Gestel, & Mommaerts, 2012). Pheasant chicks depend on forbs and insects as forage and brood sizes have been shown to be significantly lower on plots sprayed with pesticides than control plots (Rands, 1986). Furthermore, spraying can result in overall food loss because considerable mortality of nontarget arthropods beneficial to farmland birds has been shown to occur following pesticide application (Taylor, Maxwell, & Boik, 2006).

Recent clean‐farming practices have led to biological simplification of agricultural areas and loss of small, wildlife‐friendly nonfarmed habitats through disking or application of herbicides to field edges, crop corners, fence rows, stream banks, and other vegetated corridors (Chamberlain et al., 2000; Gennet et al., 2013). Such areas are important sources of food and refuge for numerous plant and insect species beneficial to farmland birds. Furthermore, new food safety standards implemented to reduce food‐borne pathogens (USDA, 2014c) now encourage California farmers to create buffer zones between field edges and wildlife habitat by cutting back adjacent riparian and wetland habitat (Gennet et al., 2013). The statewide decrease in barley, sugar beet, and sorghum production in conjunction with increases in rice production in the North Central region and nut orchard production in the Central region (see Figure S6) has led to an overall reduction in historic cropland pheasant habitat in California. Loss of heterogeneity and biodiversity in wetland and riparian systems near agricultural areas utilized by pheasant may decrease the capacity of these areas to sustain wild pheasant populations.

Some agricultural practices may increase available year‐round cover and minimize impacts on arthropod food resources leading to benefits for wild pheasant populations. For example, increased unharvested cropland in areas that are intensively farmed provides additional cover during months in which harvested cropland is bare or flooded (Nielson et al., 2008) and may prevent these areas from becoming population sinks (Wilson et al., 1997). Increasing height and acreage of stubble left over after harvesting crops has been shown to increase cover and foraging opportunities within harvested fields (Chamberlain, Wilson, & Fuller, 1999). Furthermore, use of chemicals that target specific pest species or encouraging organic farming practices (Chamberlain et al., 1999) may reduce impacts on arthropod food resources, thereby reducing effects of pesticides on bird communities (Kendall & Akerman, 1992). Additionally, studies that focus on the role of habitat quality in upland areas adjacent to agricultural areas, especially during seasons when cropland areas are unavailable, could help inform potential management actions for the benefit of pheasant.

Lastly, our study combined multiple sources of data into a single response to improve predictive outputs of environmental factors associated with pheasant declines. We provide evidence that this approach reduces potential logistical (Appendix S1) and analytical limitations (see Table S1) associated with common use of a single type of survey. For example, in our initial assessment of single‐variable effects (Table S1), the strongest evidence of support based on the joint index was observed for those variables that garnered strong evidence across all three indices independently (i.e., single‐survey response). In the case that one single index (e.g., AGTS only) disagreed with the other two in supporting a single variable, we observed added uncertainty in the joint index parameter estimate (e.g., 95% CI of parameter estimate overlapped zero). Having used only one survey type alone, results could be misleading as certain effects were subjected to under‐ or overestimation. This combining of response data approach better informs management strategies by improving confidence in those factors identified to affect pheasant abundance (e.g., 95% CI of joint index parameter estimate does not overlap zero), while preventing potential erroneous findings that might occur with responses derived from a single‐survey type.

Our approach employed multiple long‐term legacy survey data sets at relatively large spatial scales to identify factors responsible for pheasant declines observed within California. These results likely provide insight regarding population dynamics of multiple avian species that are faced with similar changing environments. This analytical approach led to findings using a single species that could help to inform environmental management and policy decisions aimed at reducing widespread declines in farmland birds in western United States, but should not be limited to pheasants as other taxa consist of similar data from multiple survey types. Furthermore, studies that focus on variation in effects across large spatial scales such as ours help managers distinguish between local patterns vs. those that are widespread, thus reducing biases often associated with site‐specific studies that could lead to misguided management efforts.

## Conflict of Interest

None declared.

## Data Accessibility

Data and associated metadata will be archived and made available through U.S. Geological Survey ScienceBase‐Catalog located at https://www.sciencebase.gov/catalog/.
