# Supplementary figures and images for: Long‐term and widespread changes in agricultural practices influence ring‐necked pheasant abundance in California
Source: Ecol Evol. 2017 Mar 15;7(8):2546–59. doi: 10.1002/ece3.2675 (PMC5395463; doi:10.1002/ece3.2675)

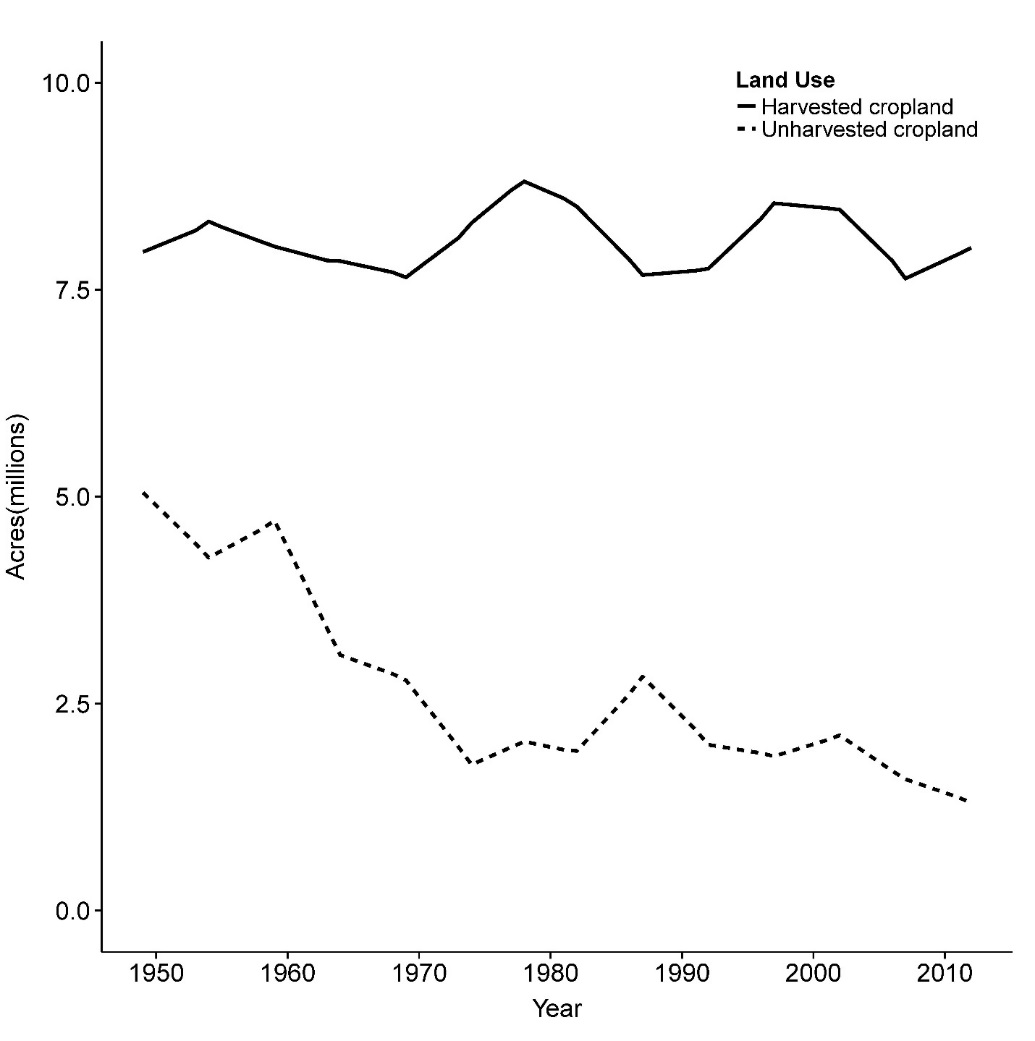

Supplement: Supplementary file 1 [file ECE3-7-2546-s001.tif]

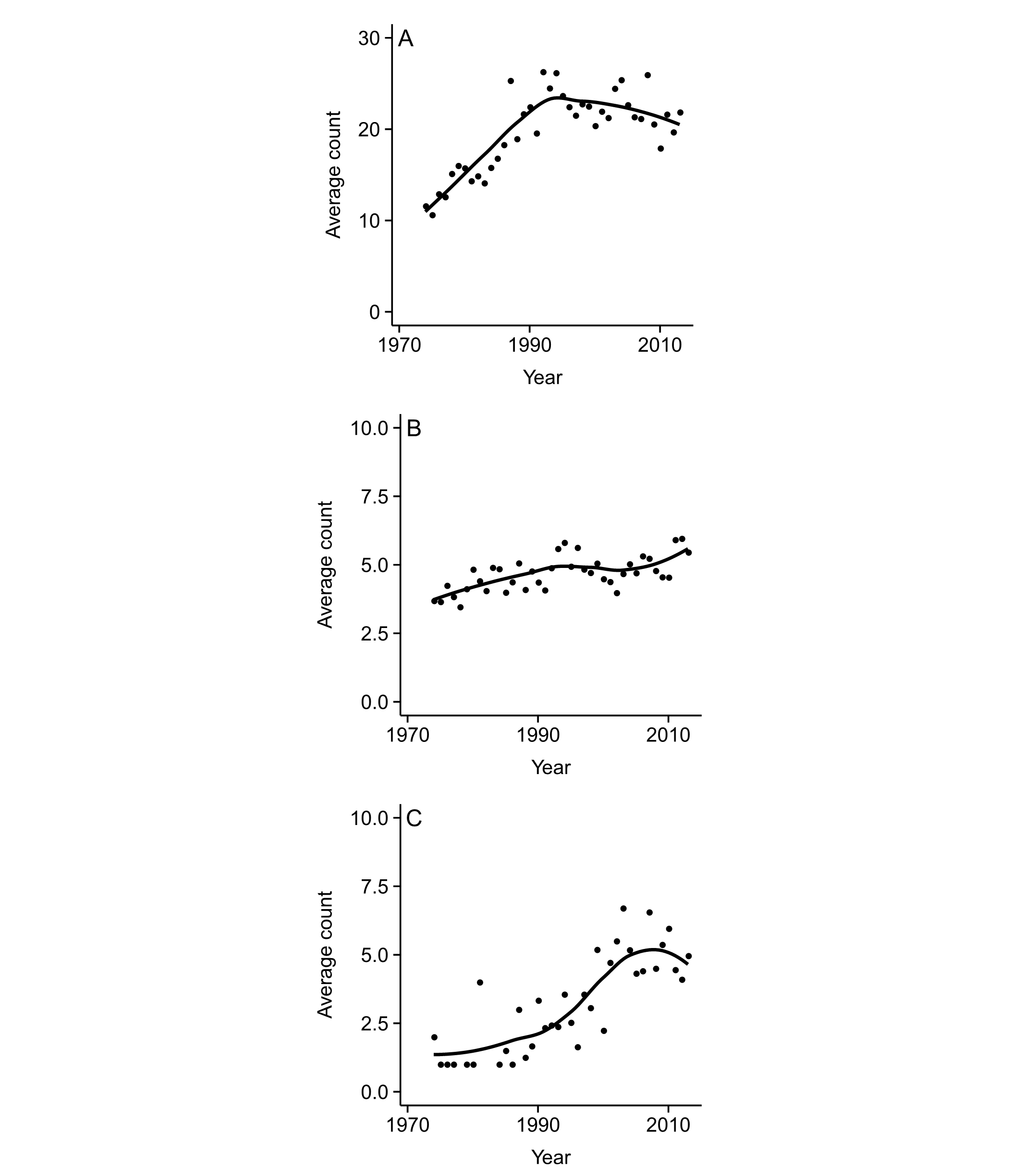

Supplement: Supplementary file 3 [file ECE3-7-2546-s003.tif]

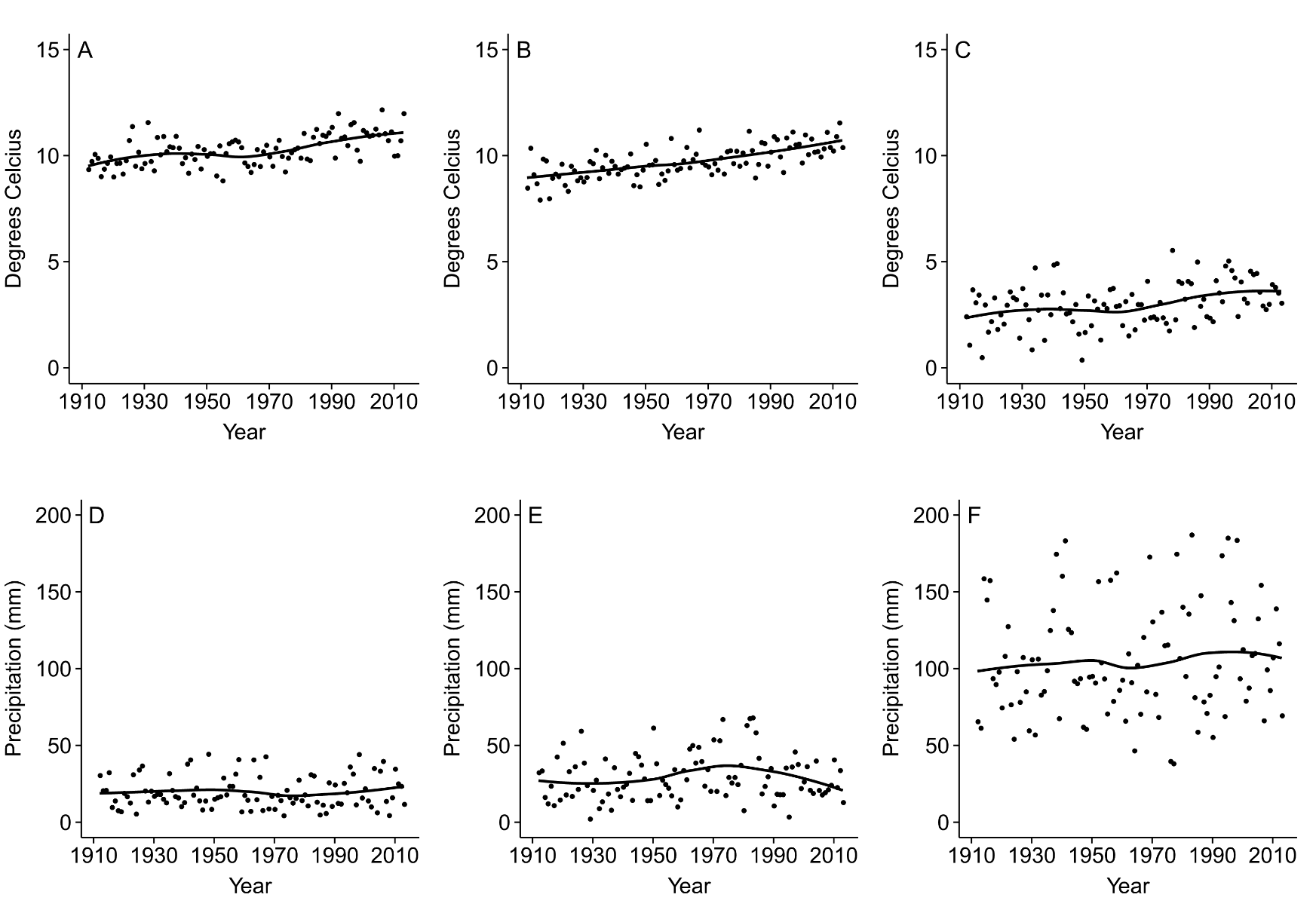

Supplement: Supplementary file 4 [file ECE3-7-2546-s004.tif]

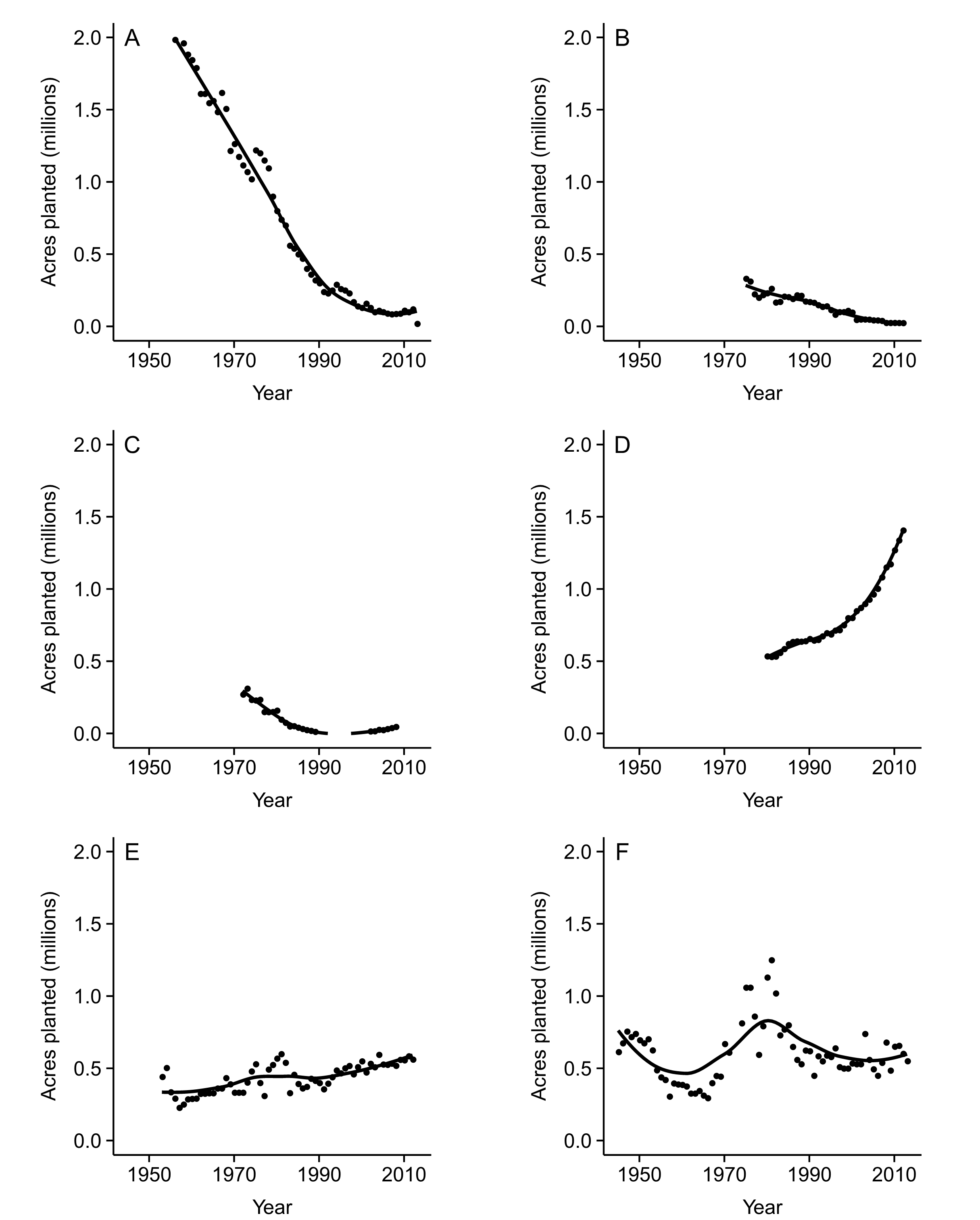

Supplement: Supplementary file 5 [file ECE3-7-2546-s005.tif]

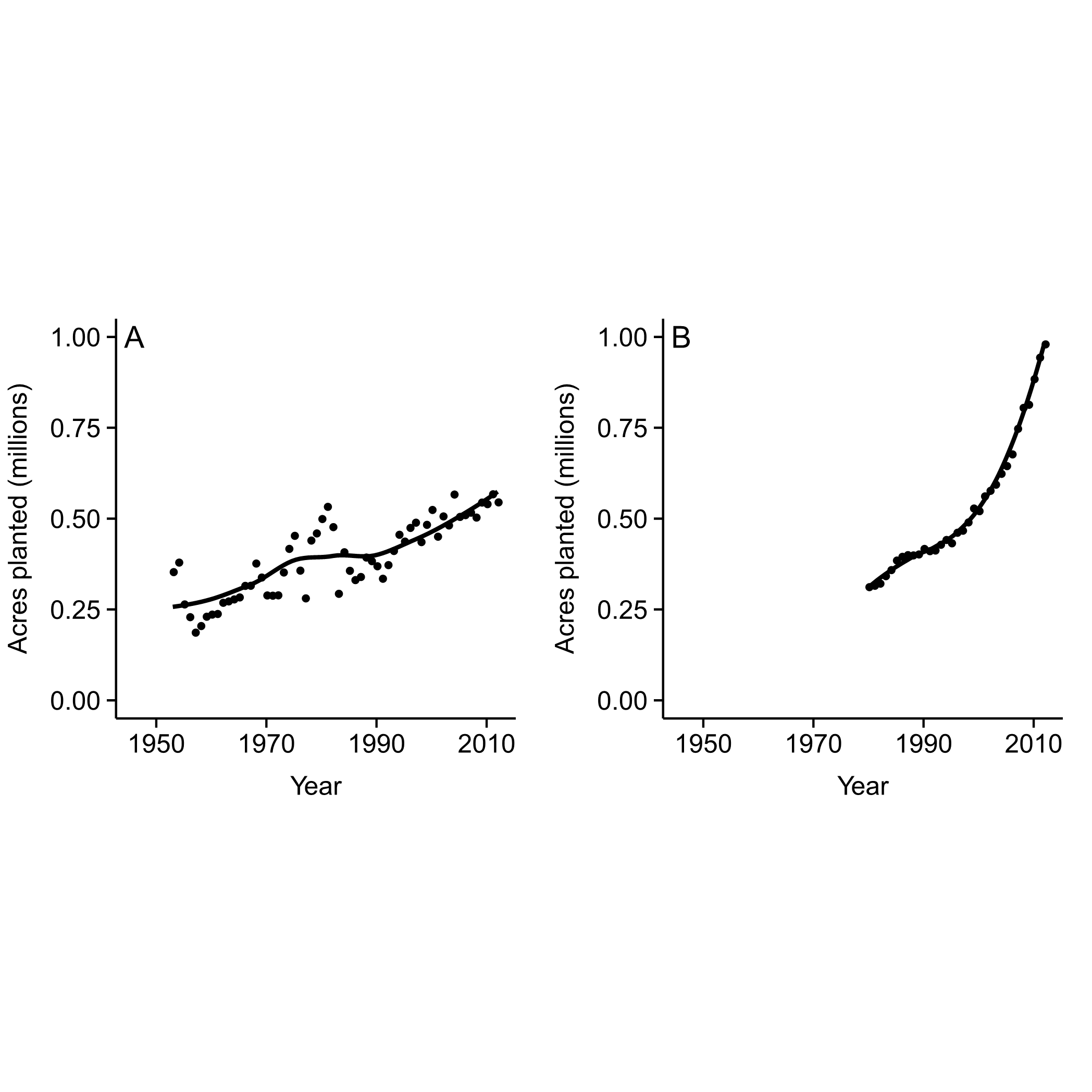

Supplement: Supplementary file 6 [file ECE3-7-2546-s006.tif]
