# Supplementary material for: Long‐term and widespread changes in agricultural practices influence ring‐necked pheasant abundance in California
Source: Ecol Evol. 2017 Mar 15;7(8):2546–59. doi: 10.1002/ece3.2675 (PMC5395463; doi:10.1002/ece3.2675)
Supplement: Supplementary file 7 [file ECE3-7-2546-s007.docx]

**Appendix S1. Summary description for methodology used in data compilation and development of joint abundance index using multiple data sources.**

**Joint Abundance Index**. We developed a joint abundance index using data from three independent sources: Christmas Bird Count (CBC), Breeding Bird Survey (BBS), and Annual Game Take Surveys (AGTS). The purpose of combining the surveys into a single response metric was to improve predictive outputs for factors that influence pheasant populations. Each survey consists of different field methodology and varies in logistical constraints. Below we describe each field methodology.

The BBS was developed in 1965 and became fully operational in 1968 (Sauer *et al.* 2014). Currently, there are over 3,700 BBS routes across the continental U.S. and Canada, of which nearly 3,000 are surveyed annually. There are currently 228 BBS routes in California. Routes are randomly located in order to sample habitats that are representative of the entire region. Each route is 24.5 miles long, with a total of 50 stops located every 0.5 mile interval along the route. At each stop a three-minute point count is conducted and observers record all birds heard or seen within 0.25 mile of the stop. Data from each stop are then totaled over the entire 50 stop route. A major constraint to BBS surveys is routes are generated only along roads and other linear right-of-ways. Survey results may not reflect accurate population abundances of species that either select or avoid linear right-of-ways. There also exist differences in the quality of observers of the BBS (Sauer *et al.* 1994). However, data are used to produce an index of abundance rather than a complete count of breeding bird populations. Thus, BBS may still serve as a reliable index for factors that influence population numbers. Additionally, surveys are conducted during the peak of nesting season (primarily May in California) and not year round. The reason for surveying during the breeding period is to maximize detection of most avian species. The BBS has become the primary source of long-term, large scale population data for more than 400 North American Breeding Bird Species (Sauer *et al.* 2014).

The CBC is the longest running volunteer survey in the world. The survey has occurred annually since 1900 and is conducted during 14 December – 5 January. Survey sampling units consist of nearly 2,400 designated “count circles”, each 15 miles in diameter, located throughout North America. A minimum of 10 observers recorded all birds seen within designated “count circles”. Data is submitted to the National Audubon Society where it is compiled and summarized in Audubon’s CBC database (National Audubon Society 2014). Logistical constraints with CBC data include varying number of observers per circle, difference in observer time, and varying levels of experience among observers. To account for potential biases associated with varying efforts, we adjusted responses by dividing the number of pheasants detected per survey by the total amount of observer time (e.g., number of observers multiplied by amount of time). Additionally, CBC data are subjected to potential seasonal biases associated with detection of species during winter months. Although pheasants are not highly visible, and CBC count precision for the species is likely low, the CBC is still likely a reliable index that can be used to detect large scale population changes. Similar to the BBS, the number of routes surveyed each winter varied over time.

The AGTS is an annual statewide mail survey that provides data on annual harvest, hunters and hunter success (harvest/hunter) by county. The survey is mailed to approximately 10–12% of licensed hunters in California. Generally 20–25% of those receiving survey forms submit harvest information to the California Department of Fish and Wildlife, representing 3–5% of licensed hunters in CA (Hart 1990). During the survey period 1948–1980, harvest of wild pheasants was not separated from game farm pheasants in annual harvest summaries. In 1981, the survey requested hunters to submit only numbers of wild pheasants harvested. Beginning in 1992 the AGTS requested that hunters report their harvest of game farm pheasants separately from their harvest of wild pheasants and game farm and wild pheasant harvests were summarized separately (CDFW 2010). Furthermore, this survey does not reflect data obtained from a random sampling design, such that responses are derived from only harvested pheasant within hunting areas.

Because each independent survey consists of specific logistical constraints, combining the responses into a single modeling framework likely minimizes potential bias associated with any one survey type. Thus, we took multiple steps to combine the three survey types to develop a joint index as a response for our analysis, expanding on methodology described in Freeman (2007). For each BBS and CBC survey, we averaged pheasant counts (pheasant count/effort for CBC) across all surveys conducted in each county during each year to develop an average abundance of pheasants detected per survey. We included all surveys that were within the defined study boundary, including zero counts. For each BBS, CBC and AGTS, the pheasant counts were normalized with a square root transformation (Neter, Wasserman & Kutner 1990). The resulting joint index consisted of the independent response variables from each independent survey represented as a single response vector. Each survey type response was then converted to the standard normal variate (mean = 0, standard deviation = 1) to facilitate interpretation of results when regressed against predictor variables. The purpose of this conversion was to create a single joint index to estimate the relative effect of predictor variables using all three data sources. Estimation of absolute effects for any one survey was not possible with this method. Each time we restricted the dataset for an analysis, we recalculated the standard normal variates for each survey type based on the new dataset. We fit a categorical dependent variable of “survey type” (i.e., BBS, CBC, or AGTS) as an additive effect in all candidate models to account for variation in the response associated with differences between survey procedures. This method allowed for improvement in parameter estimation (e.g., coefficients) of the variables of interest (e.g., land cover changes) by allowing intercepts to vary across the different survey types.

**Land Use Practices.** We collected and compiled U.S. Department of Agriculture (USDA) census data (USDA 2014a) to categorize land use practices in each county within the study area boundary during 1949–2012. We established two categories of land use: (1) harvested cropland; and (2) unharvested cropland, including cropland used as pasture, fallowed, idle lands, Conservation Reserve Program (CRP) land and Federal Crop Reduction program lands (prior to CRP).

Land use censuses are conducted roughly every 5 years. Values were imputed in non-survey years by taking the difference between the pre- and post- survey years and dividing it equally across non-surveyed years. We then calculated a percentage of each land use per county acres. We included only that area that occurred within the pheasant study area, with the assumption that most farming also takes place within this region. For the analyses, we considered both the effects of land use practices on pheasant abundance (single-variable) and the changes of the effects of land use practices through time (interaction of land use with year).

**Avian predator and competitor abundance data.** Predation of nests, chicks and adults may be an important factor limiting pheasant populations. We compiled BBS data (Sauer *et al.* 2014) indexing avian predator abundance during 1974–2012 for avian nest predators (corvids) and predators of adult pheasants (raptors). For nest predators, hereinafter called corvids, we were particularly interested in changes in the abundance of common ravens (*Corvus corax*) and American crow (*Corvus brachyrhynchos*). For adult predators, hereinafter called raptors, we included golden eagle (*Aquila chrysaetos*), bald eagle (*Haliaeetus leucocephalus*), northern harrier (*Circus cyaneus*), sharp-shinned hawk (*Accipiter striatus*), Cooper’s hawk (*Accipiter cooperii*), red-shouldered hawk (*Buteo lineatus*), Swainson’s hawk (*Buteo swainsoni*), red-tailed hawk (*Buteo jamaicensis*), barn owl (*Tyto alba*), great horned owl (*Bubo virginianus*) and short-eared owl (*Asio flammeus*). We averaged corvid and raptor counts across surveys conducted during each county/year to develop an average abundance of nest and adult predators detected per survey-year in each county.

Anecdotal evidence suggests that wild turkeys chase pheasants and interfere with attempts by roosters to attract hens. We summarized BBS data to index abundance of wild turkeys in California. We averaged turkey counts across surveys conducted in each county during each year to develop an average abundance of turkeys counted per survey-year.

**Climate data.** We used monthly PRISM (Parameter-elevation Regressions on Independent Slopes Model) climate data ([Daly *et al.* 2008](#_ENREF_9)), collected for the Basin Characterization Model for California (CA-BCM; ([Flint *et al.* 2013](#_ENREF_16)) and downscaled to 270 m grid cell size ([Flint & Flint 2012](#_ENREF_15)) for 1913–2013 (Table 1). To look at differences in climate effects throughout the lifecycle of the pheasant, we created three distinct climatic seasons: 1) winter (December–March); 2) pheasant breeding season (April–July); and 3) pheasant brood-rearing season (August–November). We averaged the climatic variables, precipitation and minimum temperature, temporally (by season/year) and spatially (by county).

**Pesticide data.** We compiled county-specific pesticide application data (total pounds applied annually) for the study area during 1990–2013 (California Department of Pesticide Regulation 2014). We calculated the amount of total pesticide application (kg/ha) for each county-year. For 11 select insecticides, Chlorpyrifos, Carbofuron, Methyl Parathion, Carbaryl, and Malathion, 3 types of neonicotinoids (including Imidacloprid), Permethrin, Copper Sulfate, and Lambda Cyhalothrin we report general trends regionally and across the state.

**Crop data.** We compiled and summarized regional crop acreage data (USDA 2014b) within the study area boundary to examine trends in available crops that may or may not provide habitat for pheasants. Available crops included: winter wheat, spring wheat, barley, oats and seed vegetation (i.e., rye grass, alfalfa, clover, sudangrass, vetch, other), rice, corn, sorghum, safflower, cotton, sugar beets and hay (i.e., alfalfa, grain, green chop hay, wild hay), grapes (i.e., raisin, table, wine, unspecified), nut trees (i.e., almonds, pistachios, English, black walnuts) and fruit trees (i.e., apples, apricots, cherries, citrus unspecified, dates, figs, grapefruit, kiwi fruit, lemons, nectarines, olives, oranges, peaches, pears, plums, prunes, tangelos, tangerines/mandarins). Values were imputed in no-data years by taking the difference between the pre- and post- years and dividing it equally across non-surveyed years. We calculated a percentage of each crop per total county acres within the study area boundary for each county-year.

**Literature Cited**

California Department of Fish and Wildlife. 2010. Game take hunter survey reports. [online]. Available at: https://www.dfg.ca.gov/wildlife/hunting/uplandgame/reports/surveys.html (13 July 2015).

California Department of Pesticide Regulation. 2014. Pesticide use report database. [online]. Available at: http://www.cdpr.ca.gov (24 July 2015).

Daly, C., Halbleib, M., Smith, J.I., Gibson, W.P., Doggett, M.K., Taylor, G.H., Curtis, J. and Pasteris, P.P. 2008. Physiographically sensitive mapping of climatological temperature and precipitation across the conterminous United States. International journal of climatology 28:2031.

Flint, L. and Flint, A. 2012. Downscaling future climate scenarios to fine scales for hydrologic and ecological modeling and analysis. Ecological Processes 1:2.

Flint, L.E., Flint, A.L., Thorne, J.H. and Boynton, R. 2013. Fine-scale hydrologic modeling for regional landscape applications: the California Basin Characterization Model development and performance. Ecological Processes 2:1-21.

Freeman, S.N., Noble, D.G., Newsom, S.E., and Baillie, S.R. 2007. Modelling population changes using data from different surveys: The Common Birds Census and the Breeding Bird Survey. Bird Study 54:61-72.

National Audubon Society. 2014. The Christmas Bird Count Historical Results [online]. Available at: http://www.christmasbirdcount.org (24 July 2015).

Hart, C. H. 1990. Management plan for the ring-necked pheasant in California. California Department of Fish and Game publication.

Neter, J., Wasserman, W., and Kutner, M.H. 1990. Applied Linear Statistical Models: regression, analysis of variance, and experimental designs, 3^rd^ edn. Irwin, Inc., Homewood, Illinois.

Sauer, J. R., Peterjohn, B. G. and Link, W.A. 1994. Observer differences in the North American Breeding Bird Survey. The Auk 111:50-62.

Sauer, J. R., Hines, J. E., Fallon, J. E., Pardieck, K. L., Ziolkowski, D. J. Jr. and Link, W. A. 2014. The North American Breeding Bird Survey, Results and Analysis 1966 - 2012. Version 02.19.2014, USGS Patuxent Wildlife Research Center, Laurel, MD.

USDA 2014a. Census of Agriculture. Available at: http://www.agcensus.usda.gov/Publications/index.php (14 July 2015)

USDA 2014b. Crop Acreage Data. Available at: http://www.fsa.usda.gov/FSA/webapp?area=newsroomandsubject=landingandtopic=foi-er-fri-cad (14 July 2015)

**Appendix S2. Procedure for calculating adjusted probabilities and evidence ratios (ER) for covariates within models with unequal representation.**

Based on unequal representation of variables across models, we carried out multiple steps to calculate adjusted evidence ratios (ER). The unadjusted ER took the form:

$$ER=\frac{{Ʃw}_{i}}{(1-{Ʃw}_{i})}$$

Where *w*_i_ is the weight of the model with the given covariate. We then adjusted the evidence ratio (Adj ER) to account for unequal representation of covariates within the model sets, which took the form:

$$Adj. ER= \frac{ER}{{\frac{n_{i}}{n_{t}}}/{(1-\frac{n_{i}}{n_{t}})}}$$

Where *n_i_* is the number of models with a given covariate, and *n_t_* is the total number of models in the model set. We then calculated an adjusted probability (Adj. probability), which took the form:

$$Adj. Probability=\frac{Adj. ER}{(1+Adj. ER)}$$

Covariates that have Adj. ER > 1, were more likely to have support from the data than covariates with Adj. ER < 1. Similarly, covariate with Adj. probability > 0.5 had support from the data.

**Appendix S3. Summary description of statewide and regional trends in pheasant abundance index and predictor variables**

**Pheasant survey data**. Pheasant populations have declined substantially over the past 25 years based on indices from all three survey techniques (Fig 2). For example, based on AGTS, annual statewide pheasant harvest declined greatly from >500,000 birds in the 1950s and 1960s to <100,000 birds/year during 2007–2012 (Fig. 2A). BBS data indicate a decreasing pheasant population beginning in 1989, and pheasant detections during 2011–2012 were the lowest recorded since the survey started in 1968 (Fig. 2B). Similarly, total pheasant detections during CBC surveys increased to a peak in the early 1980s before declining to near record lows by 2012 (Fig. 2C).

**Land use.** The total amount of harvested cropland in California remained relatively stable during 1949–2012 averaging 8.1 million acres (95% CI: 8.0 – 8.2 million; Fig. S1). However, the amount of land that remained in production varied regionally across the state, with many regions losing cropland as production became increasingly concentrated in the North Central and Central regions of the state (Fig. S2). The total amount of unharvested cropland declined by approximately 74% since 1949–2012, from ~5.0 million acres to ~1.3 million acres. CRP increased from 0 acres in 1949 to a peak of around 0.7 million acres in 1987, and has since decreased to ~0.1 million in 2012. Both cropland used as pasture and fallowed fields have experienced drastic reductions since 1949. Cropland used as pasture has had an 81% reduction in acreage from 1949 to 2012, going from over 3.5 million acres to less than 1 million acres. Likewise, the amount of fallowed cropland has declined by 65% since 1949 from ~1.5 million acres to about 0.5 acres.

**Avian predator and competitor abundance data.** BBS data indicate steady increases in corvid populations from 1974–1990, mainly along the coastal regions, after which they level off and remain stable (Fig. S3A). Raptors have steadily increased throughout the course of this study (Fig. S3B). Likewise, wild turkeys have markedly increased since 1970 (Fig. S3C).

**Climate data.** Minimum temperature has increased in all seasons by about 1 to 2 °C from 1912- 2013 (Fig. S4). The largest increase (2.6 °C) took place during the breeding season and the least change (0.63 °C) occurred during the winter. There are no evident trends in precipitation from 1912 to 2013.

**Pesticide data.** The total amount of pesticides applied annually to the California environment varied greatly during 1990-2012 but was greatest in 1993-1998, 2005, and 2011. For California overall during 1990-2012, use of 11 select insecticides had widely different use trends with Chlorpyrifos, Carbofuron, Methyl Parathion, Carbaryl, and Malathion decreasing, neonicotinoids (esp. Imidacloprid) increasing, and Permethrin, Copper Sulfate, and Lambda Cyhalothrin showing no consistent trend.

Additionally, annual amounts and patterns of use varied among regions. For California overall during 1990-2012, total pounds of human health pesticides applied peaked in 2006 (>7 million pounds of sodium hypochlorite [bleach] used in San Francisco County in 1991-1992 excluded). Petroleum (i.e., oils) comprised most of the total human health pesticides used (>7 million pounds of sodium hypochlorite [bleach] used in San Francisco County in 1991-1992 excluded) except during 2008-2012. Even more recently (starting in the 1990s), neonicontinoids, the first new class of insecticides introduced in the last 50 years, were introduced in response to widespread insect resistance and growing health and safety concerns of organophosphates. Neonicontinoids are now the most widely-used insecticides in the world with nearly 300 neonicontinoid products registered for use in California. Neonicontinoids have been linked to honey-bee colony collapse, and have been called “the new DDT” because of their persistent toxicity in the environment to aquatic invertebrates, birds, and other wildlife (Monbiot 2013). The American Bird Conservancy called for the ban of neonicontinoid products in March 2013 (American Bird Conservancy 2013). Among selected mosquito abatement pesticides, Bacillus Thuringiensis, Naled, and Piperonyl Butoxide use increased after 2004 (the year West Nile became established in California) whereas use of Malathion declined.

**Crop data.** The types of crops produced statewide have also changed considerably. Most notably, there has been a significant reduction in barley production, from ~2.1 million acres in 1954 to approximately 0.02 million acres in 2013 (Fig. S5A). Similarly, sugar beet production has declined from an average of 0.3 million acres in 1975 to ~0.02 million acres in 2012 (Fig. S5B), and sorghum production has declined from 0.3 in 1972, to approximately 0.05 million acres in 2008 (Fig. S5C). Concurrently, there were substantial increases in the production of nut trees and rice, with nut trees in the number of acres planted since 1980, from about 0.5 million acres to almost 1.5 million acres (Fig. S5D). Rice production has also increased by ~27% since 1953 (Fig. S5E). The total number of acres producing winter wheat has fluctuated through time, with a peak of 1.3 million acres in 1981 (Fig. S5F).

**Appendix S4. Within group, initial variable reduction modeling procedure**

Within the land cover type model set (county/year samples = 6,696), the top model relating pheasant abundance to land cover included an interaction between harvested cropland and year (*w* = 1.00; Table S2). The interaction with unharvested cropland and year was also supported, with ΔAIC*_c_* >2 from the baseline, and each of these covariates were carried forward to the modeling analysis. We used 3,231 county/year samples to evaluate the avian predator model set. The top model included the covariate for corvid abundance (*w* = 1.00). However, we found supporting evidence for both corvid and raptor abundance, and both covariates were carried forward to the modeling analyses. Within the competitor model set (county/year samples = 3,375), the data supported evidence for turkey abundance, thus this covariate was carried forward as well. Five covariates describing climate conditions (county/year samples = 6,887) had ΔAIC*_c_* > 2 from the baseline, and thus were carried forward.

**Table S1.** Estimated parameter estimates (β ) and 95% confidence intervals for three different survey types and a joint index from single variable generalized linear mixed effects models on ring-necked pheasant (*Phasianus colchicus*) abundance in California.

Covariates Joint Index BBS CBC AGTS

HC × Year -0.081 (-0.088 – -0.074) -0.062 (-0.077 – -0.046) -0.04 (-0.058 – -0.022) -0.082 (-0.088 – -0.076)

UC × Year -0.008 (-0.022 – 0.009) -0.116 (-0.18 – -0.056) -0.081 (-0.114 – -0.043) 0.025 (0.012 – 0.038)

UC 2.90 (2.46 – 3.33) 0.72 (-0.02 – 1.47) 2.86 (2.02 – 3.75) 3.21 (2.79 – 3.61)

HC -0.29 (-0.88 – 0.24) 0.81 (-0.36 – 1.84) 1.18 (0.29 – 2.15) 0.24 (-0.3 – 0.81)

Corv -0.004 (-0.005 – -0.003) -0.012 (-0.022 – -0.004) -0.003 (-0.005 – -0.002) -0.007 (-0.008 – -0.006)

Rapt -0.022 (-0.028 – -0.016) -0.001 (-0.002 – 0) -0.007 (-0.018 – 0.004) -0.042 (-0.051 – -0.034)

Turk -0.037 (-0.058 – -0.016) -0.031 (-0.057 – -0.004) -0.041 (-0.08 – -0.003) -0.057 (-0.085 – -0.026)

TMIN(Breed) -0.15 (-0.17 – -0.13) -0.04 (-0.07 – 0.00) -0.10 (-0.15 – -0.06) -0.23 (-0.25 – -0.21)

TMIN(Brood) -0.13 (-0.15 – -0.11) -0.05 (-0.08 – -0.01) -0.12 (-0.16 – -0.08) -0.15 (-0.17 – -0.13)

TMIN(Win) -0.07 (-0.09 – -0.06) -0.02 (-0.04 – 0.01) -0.05 (-0.08 – -0.02) -0.11 (-0.12 – -0.09)

PPT(Brood) 0.002 (0.001 – 0.003) 0.002 (0 – 0.003) 0.002 (0 – 0.004) 0.002 (0.001 – 0.003)

PPT(Breed) -0.002 (-0.003 – -0.001) -0.002 (-0.003 – 0) -0.001 (-0.004 – 0.001) -0.003 (-0.004 – -0.001)

PPT(Win) 0.000 (-0.001 – 0.000) 0.000 (-0.001 – 0.000) 0.000 (-0.001 – 0.001) 0.000 (-0.001 – 0.000)

**Table S2.** Model evaluation of variable reduction analysis using generalized linear mixed effects models on a joint index of ring-necked pheasant (*Phasianus colchicus*) abundance in California.

Group Model Covariate *K* *LL* ΔAIC*_c_* *w*

Land Use Harvested Cropland × Year^a^ 8 -6513.79 0.00 1.00

Unharvested Cropland × Year^a^ 8 -6792.72 557.86 0.00

Unharvested Cropland 6 -7115.21 1198.84 0.00

Baseline Model 5 -7211.92 1390.26 0.00

Harvested Cropland 6 -7211.44 1391.30 0.00

Random Effects Only 3 -7215.67 1393.75 0.00

Avian Predators Corvid Abundance^a^ 6 -3669.05 0.00 1.00

Raptor Abundance^a^ 6 -3682.78 27.46 0.00

Baseline Model 5 -3705.04 69.98 0.00

Random Effects Only 3 -3710.15 76.18 0.00

Competition Turkey Abundance^a^ 6 -3698.82 0.00 0.99

Baseline Model 5 -3705.04 10.44 0.01

Random Effects Only 3 -3710.15 16.64 0.00

Climate Minimum Temperature (Breeding)^a^ 6 -7331.87 0.00 1.00

Minimum Temperature (Brood-rearing)^a^ 6 -7367.82 71.90 0.00

Minimum Temperature (Winter)^a^ 6 -7389.29 114.83 0.00

Precipitation (Brood-rearing)^a^ 6 -7426.49 189.24 0.00

Precipitation (Breeding)^a^ 6 -7431.32 198.90 0.00

Baseline Model 5 -7435.69 205.63 0.00

Precipitation (Winter) 6 -7435.06 206.38 0.00

Random Effects Only 3 -7440.15 210.53 0.00

^a^ These models were carried forward to the additive modeling procedures.

Column Abbreviations: *LL* = Log(*Likelihood*); *K* = number of estimated parameters; AIC*_c_* = Akaike’s Information Criterion with second-order bias correction; ΔAIC*_c_* = difference (Δ) in AIC_c_ between best approximating model and model of interest; *w* = model probability.

**Table S3.** Correlation matrix for variables carried forward to additive generalized linear mixed effects models on a joint index of ring-necked pheasant (*Phasianus colchicus*) abundance in California.

Corv Rapt Turk TMIN(Breed) TMIN(Brood) TMIN(Win) PPT(Brood) PPT(Breed)

Corv 1.00 0.31 0.01 0.14 0.14 0.18 -0.10 -0.05

Rapt 0.31 1.00 0.08 -0.19 -0.18 -0.11 -0.17 -0.11

Turk 0.01 0.08 1.00 -0.03 0.00 0.05 -0.01 0.14

TMIN(Breed) 0.14 -0.19 -0.03 1.00 0.89 0.67 -0.29 -0.31

TMIN(Brood) 0.14 -0.18 0.00 0.89 1.00 0.82 -0.21 -0.25

TMIN(Win) 0.18 -0.11 0.05 0.67 0.82 1.00 -0.06 -0.08

PPT(Brood) -0.10 -0.17 -0.01 -0.29 -0.21 -0.06 1.00 0.41

PPT(Breed) -0.05 -0.11 0.14 -0.31 -0.25 -0.08 0.41 1.00

**Table S4.** Model evaluation of generalized linear mixed effects models on a joint index of ring-necked pheasant (*Phasianus colchicus*) abundance in California.

Model Covariate *K* *LL* ΔAIC*_c_* *w R^2^c*

HC × Year + UC × Year 10 -3390.58 0.00 0.09 0.54

HC × Year + UC × Year + PPT(Brood) 11 -3389.92 0.69 0.07 0.54

HC × Year + UC × Year + TMIN(Breed) 11 -3389.92 0.70 0.07 0.54

HC × Year + UC × Year + PPT(Breed) 11 -3390.16 1.18 0.05 0.54

HC × Year + UC × Year + TMIN(Breed) + PPT(Brood) 12 -3389.22 1.32 0.05 0.54

HC × Year + UC × Year + PPT(Brood) + PPT(Breed) 12 -3389.36 1.58 0.04 0.54

HC × Year + UC × Year + TMIN(Win) 11 -3390.52 1.90 0.04 0.54

HC × Year + UC × Year + Turk 11 -3390.54 1.94 0.04 0.54

HC × Year + UC × Year + Corv 11 -3390.55 1.95 0.04 0.54

HC × Year + UC × Year + TMIN(Brood) 11 -3390.55 1.95 0.04 0.54

HC × Year + UC × Year + Rapt 11 -3390.58 2.01 0.03 0.54

HC × Year + UC × Year + TMIN(Breed) + PPT(Breed) 12 -3389.64 2.15 0.03 0.54

HC × Year + UC × Year + TMIN(Win) + PPT(Brood) 12 -3389.87 2.61 0.03 0.54

HC × Year + UC × Year + Turk + PPT(Brood) 12 -3389.88 2.62 0.03 0.54

HC × Year + UC × Year + TMIN(Brood) + PPT(Brood) 12 -3389.88 2.62 0.03 0.54

HC × Year + UC × Year + Corv + PPT(Brood) 12 -3389.89 2.64 0.03 0.54

HC × Year + UC × Year + Corv + TMIN(Breed) 12 -3389.89 2.65 0.03 0.54

HC × Year + UC × Year + Turk + TMIN(Breed) 12 -3389.89 2.65 0.03 0.54

HC × Year + UC × Year + Rapt + PPT(Brood) 12 -3389.92 2.70 0.02 0.54

HC × Year + UC × Year + Rapt + TMIN(Breed) 12 -3389.92 2.71 0.02 0.54

HC × Year + UC × Year + Corv + PPT(Breed) 12 -3390.13 3.12 0.02 0.54

HC × Year + UC × Year + TMIN(Brood) + PPT(Breed) 12 -3390.13 3.13 0.02 0.54

HC × Year + UC × Year + Turk + PPT(Breed) 12 -3390.14 3.15 0.02 0.54

HC × Year + UC × Year + TMIN(Win) + PPT(Breed) 12 -3390.16 3.18 0.02 0.54

HC × Year + UC × Year + Rapt + PPT(Breed) 12 -3390.16 3.19 0.02 0.54

HC × Year + UC × Year + Turk + TMIN(Win) 12 -3390.49 3.85 0.01 0.54

HC × Year + UC × Year + Corv + TMIN(Win) 12 -3390.49 3.85 0.01 0.54

HC × Year + UC × Year + Corv + Turk 12 -3390.51 3.90 0.01 0.54

HC × Year + UC × Year + Turk + TMIN(Brood) 12 -3390.52 3.90 0.01 0.54

HC × Year + UC × Year + Corv + TMIN(Brood) 12 -3390.52 3.91 0.01 0.54

HC × Year + UC × Year + Rapt + TMIN(Win) 12 -3390.52 3.91 0.01 0.54

HC × Year + UC × Year + Corv + Rapt 12 -3390.54 3.95 0.01 0.54

HC × Year + UC × Year + Rapt + Turk 12 -3390.54 3.95 0.01 0.54

HC × Year + UC × Year + Rapt + TMIN(Brood) 12 -3390.55 3.96 0.01 0.54

HC × Year 8 -3400.09 14.99 0.00 0.55

HC × Year + PPT(Brood) 9 -3399.27 15.37 0.00 0.55

HC × Year + TMIN(Breed) 9 -3399.73 16.28 0.00 0.54

HC × Year + TMIN(Breed) + PPT(Brood) 10 -3398.88 16.59 0.00 0.54

HC × Year + PPT(Breed) 9 -3399.92 16.67 0.00 0.55

HC × Year + TMIN(Win) 9 -3399.96 16.75 0.00 0.55

HC × Year + PPT(Brood) + PPT(Breed) 10 -3398.99 16.83 0.00 0.55

HC × Year + Turk 9 -3400.05 16.93 0.00 0.55

HC × Year + Corv 9 -3400.07 16.97 0.00 0.55

HC × Year + Rapt 9 -3400.08 16.98 0.00 0.55

HC × Year + TMIN(Brood) 9 -3400.09 17.00 0.00 0.55

HC × Year + TMIN(Win) + PPT(Brood) 10 -3399.16 17.17 0.00 0.55

HC × Year + Turk + PPT(Brood) 10 -3399.23 17.30 0.00 0.55

HC × Year + Corv + PPT(Brood) 10 -3399.26 17.35 0.00 0.55

HC × Year + Rapt + PPT(Brood) 10 -3399.26 17.36 0.00 0.55

HC × Year + TMIN(Brood) + PPT(Brood) 10 -3399.27 17.38 0.00 0.55

HC × Year + TMIN(Breed) + PPT(Breed) 10 -3399.63 18.09 0.00 0.54

HC × Year + TMIN(Breed) + PPT(Brood) + PPT(Breed) 11 -3398.69 18.22 0.00 0.54

HC × Year + Turk + TMIN(Breed) 10 -3399.70 18.23 0.00 0.54

HC × Year + Corv + TMIN(Breed) + 10 -3399.71 18.27 0.00 0.54

HC × Year + Rapt + TMIN(Breed) 10 -3399.72 18.28 0.00 0.54

HC × Year + Turk + TMIN(Breed) + PPT(Brood) 11 -3398.84 18.53 0.00 0.54

HC × Year + TMIN(Win) + PPT(Breed) 10 -3399.86 18.55 0.00 0.55

HC × Year + Corv + TMIN(Breed) + PPT(Brood) 11 -3398.86 18.57 0.00 0.54

HC × Year + Rapt + TMIN(Breed) + PPT(Brood) 11 -3398.87 18.59 0.00 0.54

HC × Year + Turk + PPT(Breed) 10 -3399.89 18.63 0.00 0.55

HC × Year + Corv + PPT(Breed) 10 -3399.91 18.65 0.00 0.55

HC × Year + Rapt + PPT(Breed) 10 -3399.91 18.66 0.00 0.55

HC × Year + TMIN(Brood) + PPT(Breed) 10 -3399.92 18.68 0.00 0.55

HC × Year + Turk + TMIN(Win) 10 -3399.93 18.69 0.00 0.55

HC × Year + Corv + TMIN(Win) 10 -3399.95 18.74 0.00 0.55

HC × Year + Rapt + TMIN(Win) 10 -3399.95 18.75 0.00 0.55

HC × Year + TMIN(Win) + PPT(Brood) + PPT(Breed) 11 -3398.96 18.77 0.00 0.55

HC × Year + Turk + PPT(Brood) + PPT(Breed) 11 -3398.96 18.78 0.00 0.55

HC × Year + Corv + PPT(Brood) + PPT(Breed) 11 -3398.98 18.81 0.00 0.55

HC × Year + Rapt + PPT(Brood) + PPT(Breed) 11 -3398.98 18.82 0.00 0.55

HC × Year + TMIN(Brood) + PPT(Brood) + PPT(Breed) 11 -3398.99 18.84 0.00 0.55

HC × Year + Corv + Turk 10 -3400.04 18.92 0.00 0.55

HC × Year + Rapt + Turk 10 -3400.04 18.92 0.00 0.55

HC × Year + Turk + TMIN(Brood) 10 -3400.05 18.94 0.00 0.55

HC × Year + Corv + Rapt 10 -3400.06 18.95 0.00 0.55

HC × Year + Corv + TMIN(Brood) 10 -3400.07 18.98 0.00 0.55

HC × Year + Rapt + TMIN(Brood) 10 -3400.08 19.00 0.00 0.55

HC × Year + Turk + TMIN(Win) + PPT(Brood) 11 -3399.12 19.09 0.00 0.55

HC × Year + Corv + TMIN(Win) + PPT(Brood) 11 -3399.15 19.15 0.00 0.55

HC × Year + Rapt + TMIN(Win) + PPT(Brood) 11 -3399.15 19.15 0.00 0.55

HC × Year + Rapt + Turk + PPT(Brood) 11 -3399.21 19.28 0.00 0.55

HC × Year + Corv + Turk + PPT(Brood) 11 -3399.21 19.28 0.00 0.55

HC × Year + Turk + TMIN(Brood) + PPT(Brood) 11 -3399.23 19.31 0.00 0.55

HC × Year + Corv + Rapt + PPT(Brood) 11 -3399.24 19.32 0.00 0.55

HC × Year + Corv + TMIN(Brood) + PPT(Brood) 11 -3399.26 19.36 0.00 0.55

HC × Year + Rapt + TMIN(Brood) + PPT(Brood) 11 -3399.26 19.37 0.00 0.55

HC × Year + Turk + TMIN(Breed) + PPT(Breed) 11 -3399.60 20.06 0.00 0.54

HC × Year + Corv + TMIN(Breed) + PPT(Breed) 11 -3399.61 20.08 0.00 0.54

HC × Year + Rapt + TMIN(Breed) + PPT(Breed) 11 -3399.62 20.09 0.00 0.54

HC × Year + Corv + Turk + TMIN(Breed) 11 -3399.68 20.22 0.00 0.54

HC × Year + Rapt + Turk + TMIN(Breed) 11 -3399.69 20.23 0.00 0.54

HC × Year + Corv + Rapt + TMIN(Breed) 11 -3399.70 20.25 0.00 0.54

HC × Year + Turk + TMIN(Win) + PPT(Breed) 11 -3399.83 20.51 0.00 0.55

HC × Year + Corv + TMIN(Win) + PPT(Breed) 11 -3399.84 20.53 0.00 0.55

HC × Year + Rapt + TMIN(Win) + PPT(Breed) 11 -3399.85 20.55 0.00 0.55

HC × Year + Corv + Turk + PPT(Breed) 11 -3399.88 20.61 0.00 0.55

HC × Year + Rapt + Turk + PPT(Breed) 11 -3399.88 20.62 0.00 0.55

HC × Year + Corv + Rapt + PPT(Breed) 11 -3399.89 20.63 0.00 0.55

HC × Year + Turk + TMIN(Brood) + PPT(Breed) 11 -3399.89 20.64 0.00 0.55

HC × Year + Corv + TMIN(Brood) + PPT(Breed) 11 -3399.91 20.66 0.00 0.55

HC × Year + Rapt + TMIN(Brood) + PPT(Breed) 11 -3399.91 20.68 0.00 0.55

HC × Year + Corv + Turk + TMIN(Win) 11 -3399.91 20.68 0.00 0.55

HC × Year + Rapt + Turk + TMIN(Win) 11 -3399.92 20.68 0.00 0.55

HC × Year + Corv + Rapt + TMIN(Win) 11 -3399.93 20.71 0.00 0.55

HC × Year + Corv + Rapt + Turk 11 -3400.02 20.89 0.00 0.55

HC × Year + Corv + Turk + TMIN(Brood) 11 -3400.04 20.93 0.00 0.55

HC × Year + Rapt + Turk + TMIN(Brood) 11 -3400.04 20.94 0.00 0.55

HC × Year + Corv + Rapt + TMIN(Brood) 11 -3400.06 20.97 0.00 0.55

UC × Year + Rapt + Turk + PPT(Breed) 11 -3482.07 184.99 0.00 0.60

UC × Year + Corv + Turk + PPT(Breed) 11 -3482.13 185.10 0.00 0.60

UC × Year + Turk + PPT(Breed) 10 -3483.20 185.25 0.00 0.60

UC × Year + Turk + TMIN(Breed) + PPT(Breed) 11 -3482.47 185.80 0.00 0.60

UC × Year + Rapt + Turk 10 -3483.59 186.02 0.00 0.59

UC × Year + Rapt + Turk + TMIN(Breed) 11 -3482.59 186.03 0.00 0.60

UC × Year + Corv + Turk + TMIN(Breed) 11 -3482.61 186.06 0.00 0.61

UC × Year + Corv + Turk 10 -3483.64 186.12 0.00 0.60

UC × Year + Turk + TMIN(Breed) 10 -3483.72 186.27 0.00 0.60

UC × Year + Turk 9 -3484.77 186.37 0.00 0.59

UC × Year + Corv + Rapt + Turk 11 -3482.95 186.75 0.00 0.60

UC × Year + Turk + PPT(Brood) + PPT(Breed) 11 -3483.12 187.09 0.00 0.60

UC × Year + Turk + TMIN(Win) + PPT(Breed) 11 -3483.18 187.22 0.00 0.60

UC × Year + Turk + TMIN(Brood) + PPT(Breed) 11 -3483.20 187.25 0.00 0.60

UC × Year + Corv + PPT(Breed) 10 -3484.25 187.35 0.00 0.60

UC × Year + PPT(Breed) 9 -3485.32 187.47 0.00 0.59

UC × Year + Rapt + PPT(Breed) 10 -3484.32 187.49 0.00 0.60

UC × Year + Rapt + Turk + TMIN(Win) 11 -3483.32 187.50 0.00 0.59

UC × Year + Corv + Turk + TMIN(Win) 11 -3483.43 187.71 0.00 0.60

UC × Year + Corv + TMIN(Breed) + PPT(Breed) 11 -3483.52 187.90 0.00 0.61

UC × Year + Turk + TMIN(Win) 10 -3484.55 187.94 0.00 0.59

UC × Year + Rapt + Turk + PPT(Brood) 11 -3483.56 187.98 0.00 0.59

UC × Year + TMIN(Breed) + PPT(Breed) 10 -3484.58 187.99 0.00 0.60

UC × Year + Rapt + Turk + TMIN(Brood) 11 -3483.58 188.02 0.00 0.59

UC × Year + Rapt + TMIN(Breed) + PPT(Breed) 11 -3483.62 188.10 0.00 0.60

UC × Year + Corv + Turk + PPT(Brood) 11 -3483.62 188.10 0.00 0.60

UC × Year + Corv + Turk + TMIN(Brood) 11 -3483.63 188.12 0.00 0.60

UC × Year + Turk + TMIN(Breed) + PPT(Brood) 11 -3483.69 188.23 0.00 0.60

UC × Year + Corv + Rapt + PPT(Breed) 11 -3483.70 188.25 0.00 0.60

UC × Year + Turk + PPT(Brood) 10 -3484.76 188.36 0.00 0.59

UC × Year + Turk + TMIN(Brood) 10 -3484.77 188.38 0.00 0.59

UC × Year + Corv + TMIN(Breed) 10 -3484.97 188.79 0.00 0.60

UC × Year + Corv 9 -3486.06 188.95 0.00 0.59

UC × Year + TMIN(Breed) 9 -3486.08 188.98 0.00 0.60

UC × Year + Rapt + TMIN(Breed) 10 -3485.09 189.03 0.00 0.60

UC × Year + Rapt 9 -3486.15 189.13 0.00 0.59

UC × Year 8 -3487.19 189.19 0.00 0.59

UC × Year + Corv + PPT(Brood) + PPT(Breed) 11 -3484.19 189.23 0.00 0.60

UC × Year + Corv + TMIN(Brood) + PPT(Breed) 11 -3484.24 189.32 0.00 0.60

UC × Year + Corv + TMIN(Win) + PPT(Breed) 11 -3484.24 189.34 0.00 0.60

UC × Year + Rapt + PPT(Brood) + PPT(Breed) 11 -3484.24 189.34 0.00 0.60

UC × Year + PPT(Brood) + PPT(Breed) 10 -3485.26 189.36 0.00 0.60

UC × Year + Rapt + TMIN(Win) + PPT(Breed) 11 -3484.30 189.45 0.00 0.60

UC × Year + TMIN(Win) + PPT(Breed) 10 -3485.31 189.45 0.00 0.59

UC × Year + TMIN(Brood) + PPT(Breed) 10 -3485.31 189.45 0.00 0.60

UC × Year + Rapt + TMIN(Brood) + PPT(Breed) 11 -3484.31 189.47 0.00 0.60

UC × Year + Corv + Rapt + TMIN(Breed) 11 -3484.44 189.73 0.00 0.60

UC × Year + Corv + Rapt 10 -3485.49 189.81 0.00 0.59

UC × Year + TMIN(Breed) + PPT(Brood) + PPT(Breed) 11 -3484.50 189.86 0.00 0.60

UC × Year + Turk + TMIN(Win) + PPT(Brood) 11 -3484.54 189.93 0.00 0.59

UC × Year + Turk + TMIN(Brood) + PPT(Brood) 11 -3484.76 190.37 0.00 0.59

UC × Year + Corv + TMIN(Win) 10 -3485.84 190.52 0.00 0.59

UC × Year + Rapt + TMIN(Win) 10 -3485.88 190.59 0.00 0.59

UC × Year + TMIN(Win) 9 -3486.95 190.74 0.00 0.59

UC × Year + Corv + TMIN(Breed) + PPT(Brood) 11 -3484.96 190.77 0.00 0.60

UC × Year + Corv + TMIN(Brood) 10 -3486.05 190.93 0.00 0.59

UC × Year + Corv + PPT(Brood) 10 -3486.06 190.95 0.00 0.59

UC × Year + TMIN(Breed) + PPT(Brood) 10 -3486.06 190.97 0.00 0.60

UC × Year + Rapt + TMIN(Breed) + PPT(Brood) 11 -3485.07 191.00 0.00 0.60

UC × Year + Rapt + TMIN(Brood) 10 -3486.14 191.12 0.00 0.59

UC × Year + Rapt + PPT(Brood) 10 -3486.14 191.12 0.00 0.59

UC × Year + TMIN(Brood) 9 -3487.18 191.18 0.00 0.59

UC × Year + PPT(Brood) 9 -3487.18 191.19 0.00 0.59

UC × Year + Corv + Rapt + TMIN(Win) 11 -3485.23 191.32 0.00 0.59

UC × Year + TMIN(Brood) + PPT(Brood) + PPT(Breed) 11 -3485.24 191.34 0.00 0.60

UC × Year + TMIN(Win) + PPT(Brood) + PPT(Breed) 11 -3485.25 191.35 0.00 0.60

UC × Year + Corv + Rapt + TMIN(Brood) 11 -3485.47 191.80 0.00 0.59

UC × Year + Corv + Rapt + PPT(Brood) 11 -3485.48 191.81 0.00 0.59

UC × Year + Corv + TMIN(Win) + PPT(Brood) 11 -3485.84 192.53 0.00 0.59

UC × Year + Rapt + TMIN(Win) + PPT(Brood) 11 -3485.87 192.59 0.00 0.59

UC × Year + TMIN(Win) + PPT(Brood) 10 -3486.95 192.74 0.00 0.59

UC × Year + Corv + TMIN(Brood) + PPT(Brood) 11 -3486.04 192.94 0.00 0.59

UC × Year + Rapt + TMIN(Brood) + PPT(Brood) 11 -3486.13 193.11 0.00 0.59

UC × Year + TMIN(Brood) + PPT(Brood) 10 -3487.17 193.19 0.00 0.59

Corv + Rapt + TMIN(Breed) + PPT(Brood) 9 -3625.03 466.88 0.00 0.68

Corv + Rapt + TMIN(Breed) + PPT(Breed) 9 -3627.95 472.73 0.00 0.67

Corv + TMIN(Breed) + PPT(Brood) + PPT(Breed) 9 -3628.46 473.76 0.00 0.68

Corv + Turk + TMIN(Breed) + PPT(Brood) 9 -3629.39 475.60 0.00 0.68

Corv + TMIN(Breed) + PPT(Brood) 8 -3630.96 476.74 0.00 0.69

Corv + Rapt + Turk + TMIN(Breed) 9 -3630.01 476.85 0.00 0.67

Corv + Rapt + TMIN(Breed) 8 -3631.73 478.28 0.00 0.67

Corv + Turk + TMIN(Breed) + PPT(Breed) 9 -3632.02 480.88 0.00 0.67

Corv + TMIN(Breed) + PPT(Breed) 8 -3633.52 481.86 0.00 0.68

Corv + Rapt + TMIN(Brood) + PPT(Brood) 9 -3633.04 482.91 0.00 0.66

Corv + Turk + TMIN(Breed) 8 -3635.40 485.62 0.00 0.68

Corv + TMIN(Breed) 7 -3637.58 487.98 0.00 0.68

Rapt + TMIN(Breed) + PPT(Brood) + PPT(Breed) 9 -3637.82 492.48 0.00 0.67

Corv + Turk + TMIN(Brood) + PPT(Brood) 9 -3638.10 493.03 0.00 0.66

Corv + Rapt + Turk + TMIN(Brood) 9 -3638.15 493.13 0.00 0.65

Corv + Rapt + TMIN(Brood) + PPT(Breed) 9 -3638.63 494.09 0.00 0.64

Corv + TMIN(Brood) + PPT(Brood) 8 -3639.96 494.74 0.00 0.67

Rapt + Turk + TMIN(Breed) + PPT(Brood) 9 -3639.06 494.95 0.00 0.67

Corv + Rapt + TMIN(Brood) 8 -3640.13 495.08 0.00 0.65

Corv + TMIN(Brood) + PPT(Brood) + PPT(Breed) 9 -3639.28 495.39 0.00 0.66

Rapt + TMIN(Breed) + PPT(Brood) 8 -3640.62 496.07 0.00 0.68

Rapt + Turk + TMIN(Breed) + PPT(Breed) 9 -3642.36 501.55 0.00 0.66

Rapt + TMIN(Breed) + PPT(Breed) 8 -3643.88 502.57 0.00 0.66

Corv + Turk + TMIN(Brood) + PPT(Breed) 9 -3643.24 503.30 0.00 0.65

Corv + Turk + TMIN(Brood) 8 -3644.44 503.70 0.00 0.65

Corv + TMIN(Brood) + PPT(Breed) 8 -3645.35 505.52 0.00 0.65

Corv + TMIN(Brood) 7 -3646.99 506.78 0.00 0.66

Rapt + Turk + TMIN(Breed) 8 -3646.26 507.34 0.00 0.66

Corv + Rapt + TMIN(Win) + PPT(Brood) 9 -3646.47 509.78 0.00 0.61

Rapt + TMIN(Breed) 7 -3648.52 509.84 0.00 0.66

Rapt + Turk + TMIN(Brood) + PPT(Brood) 9 -3648.69 514.22 0.00 0.64

Rapt + TMIN(Brood) + PPT(Brood) 8 -3650.55 515.91 0.00 0.65

Rapt + TMIN(Brood) + PPT(Brood) + PPT(Breed) 9 -3649.74 516.30 0.00 0.64

Turk + TMIN(Breed) + PPT(Brood) + PPT(Breed) 9 -3650.78 518.39 0.00 0.67

Corv + Rapt + Turk + PPT(Brood) 9 -3650.98 518.78 0.00 0.60

Corv + Turk + TMIN(Win) + PPT(Brood) 9 -3651.12 519.08 0.00 0.61

Corv + Rapt + Turk + TMIN(Win) 9 -3651.16 519.15 0.00 0.60

TMIN(Breed) + PPT(Brood) + PPT(Breed) 8 -3652.69 520.20 0.00 0.68

Corv + Rapt + PPT(Brood) + PPT(Breed) 9 -3651.95 520.72 0.00 0.60

Corv + Rapt + PPT(Brood) 8 -3653.29 521.40 0.00 0.60

Corv + TMIN(Win) + PPT(Brood) 8 -3653.48 521.77 0.00 0.61

Turk + TMIN(Breed) + PPT(Brood) 8 -3653.60 522.02 0.00 0.68

Corv + Rapt + TMIN(Win) 8 -3653.66 522.14 0.00 0.60

Corv + Rapt + TMIN(Win) + PPT(Breed) 9 -3652.96 522.74 0.00 0.60

Corv + TMIN(Win) + PPT(Brood) + PPT(Breed) 9 -3653.34 523.50 0.00 0.61

TMIN(Breed) + PPT(Brood) 7 -3656.16 525.12 0.00 0.69

Rapt + Turk + TMIN(Brood) + PPT(Breed) 9 -3654.86 526.55 0.00 0.63

Rapt + Turk + TMIN(Brood) 8 -3656.34 527.50 0.00 0.63

Turk + TMIN(Breed) + PPT(Breed) 8 -3656.40 527.62 0.00 0.67

Corv + Rapt + Turk + PPT(Breed) 9 -3655.74 528.30 0.00 0.59

Rapt + TMIN(Brood) + PPT(Breed) 8 -3657.02 528.85 0.00 0.63

Corv + Turk + TMIN(Win) 8 -3657.46 529.74 0.00 0.60

Corv + Rapt + Turk 8 -3657.74 530.29 0.00 0.59

TMIN(Breed) + PPT(Breed) 7 -3658.81 530.43 0.00 0.67

Rapt + TMIN(Brood) 7 -3658.98 530.77 0.00 0.64

Corv + Turk + TMIN(Win) + PPT(Breed) 9 -3657.04 530.90 0.00 0.60

Corv + Rapt + PPT(Breed) 8 -3658.22 531.26 0.00 0.59

Corv + Turk + PPT(Brood) + PPT(Breed) 9 -3657.42 531.67 0.00 0.59

Corv + Turk + PPT(Brood) 8 -3658.52 531.85 0.00 0.60

Corv + TMIN(Win) 7 -3660.58 533.96 0.00 0.61

Corv + Rapt 7 -3660.85 534.51 0.00 0.59

Corv + TMIN(Win) + PPT(Breed) 8 -3659.88 534.57 0.00 0.60

Turk + TMIN(Breed) 7 -3660.89 534.58 0.00 0.67

Corv + PPT(Brood) + PPT(Breed) 8 -3660.02 534.86 0.00 0.60

Corv + PPT(Brood) 7 -3661.55 535.91 0.00 0.60

TMIN(Breed) 6 -3664.33 539.45 0.00 0.68

Corv + Turk + PPT(Breed) 8 -3663.00 540.81 0.00 0.59

Corv + Turk 7 -3665.12 543.04 0.00 0.59

Rapt + Turk + TMIN(Win) + PPT(Brood) 9 -3663.36 543.55 0.00 0.59

Corv + PPT(Breed) 7 -3666.17 545.16 0.00 0.59

Turk + TMIN(Brood) + PPT(Brood) 8 -3665.66 546.13 0.00 0.65

Rapt + TMIN(Win) + PPT(Brood) 8 -3665.78 546.37 0.00 0.59

Turk + TMIN(Brood) + PPT(Brood) + PPT(Breed) 9 -3664.92 546.66 0.00 0.64

Rapt + TMIN(Win) + PPT(Brood) + PPT(Breed) 9 -3665.55 547.92 0.00 0.59

Corv 6 -3669.05 548.89 0.00 0.59

TMIN(Brood) + PPT(Brood) + PPT(Breed) 8 -3667.65 550.12 0.00 0.65

TMIN(Brood) + PPT(Brood) 7 -3668.74 550.28 0.00 0.66

Rapt + Turk + PPT(Brood) + PPT(Breed) 9 -3669.33 555.48 0.00 0.58

Rapt + Turk + PPT(Brood) 8 -3670.62 556.05 0.00 0.58

Rapt + Turk + TMIN(Win) 8 -3671.08 556.98 0.00 0.58

Rapt + Turk + TMIN(Win) + PPT(Breed) 9 -3670.44 557.72 0.00 0.58

Turk + TMIN(Brood) + PPT(Breed) 8 -3671.71 558.23 0.00 0.63

Rapt + PPT(Brood) + PPT(Breed) 8 -3671.92 558.65 0.00 0.58

Turk + TMIN(Brood) 7 -3673.44 559.69 0.00 0.64

Rapt + PPT(Brood) 7 -3673.67 560.15 0.00 0.59

Rapt + TMIN(Win) 7 -3674.39 561.59 0.00 0.58

Rapt + TMIN(Win) + PPT(Breed) 8 -3673.40 561.63 0.00 0.58

TMIN(Brood) + PPT(Breed) 7 -3675.13 563.08 0.00 0.63

TMIN(Brood) 6 -3677.55 565.90 0.00 0.64

Rapt + Turk + PPT(Breed) 8 -3676.14 567.10 0.00 0.57

Rapt + Turk 7 -3678.68 570.17 0.00 0.57

Rapt + PPT(Breed) 7 -3679.41 571.62 0.00 0.57

Rapt 6 -3682.78 576.35 0.00 0.57

Turk + TMIN(Win) + PPT(Brood) 8 -3680.90 576.61 0.00 0.58

Turk + TMIN(Win) + PPT(Brood) + PPT(Breed) 9 -3680.78 578.39 0.00 0.58

TMIN(Win) + PPT(Brood) 7 -3684.71 582.23 0.00 0.59

TMIN(Win) + PPT(Brood) + PPT(Breed) 8 -3684.44 583.69 0.00 0.58

Turk + TMIN(Win) 7 -3688.73 590.27 0.00 0.57

Turk + TMIN(Win) + PPT(Breed) 8 -3688.09 590.99 0.00 0.57

Turk + PPT(Brood) + PPT(Breed) 8 -3688.97 592.75 0.00 0.57

Turk + PPT(Brood) 7 -3690.58 593.97 0.00 0.57

TMIN(Win) + PPT(Breed) 7 -3692.59 597.99 0.00 0.57

TMIN(Win) 6 -3693.68 598.16 0.00 0.58

PPT(Brood) + PPT(Breed) 7 -3693.16 599.13 0.00 0.57

PPT(Brood) 6 -3695.46 601.72 0.00 0.57

Turk + PPT(Breed) 7 -3695.83 604.46 0.00 0.56

Turk 6 -3698.82 608.43 0.00 0.56

PPT(Breed) 6 -3700.88 612.56 0.00 0.56

Baseline Model 5 -3705.04 618.87 0.00 0.56

Random Effects Only 3 -3710.15 625.07 0.00 0.56

Column Abbreviations: *LL* = Log(*Likelihood*); *K* = number of estimated parameters; AIC*_c_* = Akaike’s Information Criterion with second-order bias correction; ΔAIC*_c_* = difference (Δ) in AIC_c_ between best approximating model and model of interest; *w* = model probability.

**Table S5.** Model evaluation of variable reduction analysis using generalized linear mixed effects models on a joint index of ring-necked pheasants (*Phasianus colchicus*) abundance in the Northern Region of California.

Group Model Covariate *K* *LL* ΔAIC*_c_* *w*

Land Use Harvested Cropland × Year ^a^ 8 -1130.11 0.00 1.00

Unharvested Cropland × Year ^a^ 8 -1144.99 29.77 0.00

Harvested Cropland 6 -1211.55 158.82 0.00

Random Effects Only 3 -1231.19 192.05 0.00

Unharvested Cropland 6 -1229.49 194.70 0.00

Baseline Model 5 -1231.13 195.96 0.00

Avian Predators Random Effects Only 3 -867.35 0.00 0.42

Raptor Abundance 6 -864.63 0.63 0.30

Corvid Abundance 6 -865.12 1.62 0.19

Baseline Model 5 -866.82 2.98 0.09

Competition Random Effects Only 3 -867.35 0.00 0.62

Turkey Abundance 6 -865.25 1.87 0.24

Baseline Model 5 -866.82 2.98 0.14

Climate Minimum temperature (Breeding) ^a^ 6 -1239.31 0.00 0.93

Minimum temperature (Winter) ^a^ 6 -1242.47 6.31 0.04

Minimum temperature (Brood-rearing) ^a^ 6 -1242.72 6.80 0.03

Random Effects Only 3 -1248.23 11.77 0.00

Precipitation (Breeding) 6 -1246.15 13.68 0.00

Baseline Model 5 -1248.16 15.67 0.00

Precipitation (Brood-rearing) 6 -1247.71 16.79 0.00

Precipitation (Winter) 6 -1247.88 17.13 0.00

^a^ These models were carried forward to the second step of the modeling process.

Column Abbreviations: *LL* = Log(*Likelihood*); *K* = number of estimated parameters; AIC*_c_* = Akaike’s Information Criterion with second-order bias correction; ΔAIC*_c_* = difference (Δ) in AIC_c_ between best approximating model and model of interest; *w* = model probability.

**Table S6.** Model evaluation of generalized linear mixed effects models on a joint index of ring-necked pheasant (*Phasianus colchicus*) abundance in the Northern Region of California.

Model Covariate *K* *LL* ΔAIC*_c_* *w R^2^c*

HC × Year + UC × Year 10 -1108.04 0.00 0.44 0.49

HC × Year + UC × Year + TMIN(Breed) 11 -1107.60 1.16 0.24 0.47

HC × Year + UC × Year + TMIN(Win) 11 -1108.02 2.01 0.16 0.49

HC × Year + UC × Year + TMIN(Brood) 11 -1108.03 2.01 0.16 0.49

HC × Year 8 -1130.11 40.06 0.00 0.47

HC × Year + TMIN(Brood) 9 -1129.72 41.32 0.00 0.47

HC × Year + TMIN(Breed) 9 -1130.01 41.89 0.00 0.47

HC × Year + TMIN(Win) 9 -1130.10 42.08 0.00 0.48

UC × Year 8 -1144.99 69.83 0.00 0.50

UC × Year + TMIN(Brood) 9 -1144.68 71.23 0.00 0.47

UC × Year + TMIN(Breed) 9 -1144.85 71.58 0.00 0.52

UC × Year + TMIN(Win) 9 -1144.93 71.73 0.00 0.51

TMIN(Breed) 6 -1222.09 219.97 0.00 0.45

TMIN(Win) 6 -1225.36 226.51 0.00 0.35

TMIN(Brood) 6 -1225.88 227.54 0.00 0.39

Random Effects Only 3 -1231.19 232.11 0.00 0.42

Baseline Model 5 -1231.13 236.02 0.00 0.42

Column Abbreviations: *LL* = Log(*Likelihood*); *K* = number of estimated parameters; AIC*_c_* = Akaike’s Information Criterion with second-order bias correction; ΔAIC*_c_* = difference (Δ) in AIC_c_ between best approximating model and model of interest; *w* = model probability.

**Table S7.** Model evaluation of variable reduction analysis using generalized linear mixed effects models on a joint index of ring-necked pheasants (*Phasianus colchicus*) abundance in the North Central Region of California.

Group Model Covariate *K* *LL* ΔAIC*_c_* *w*

Land Use Harvested Cropland × Year ^a^ 8 -1046.69 0.00 1.00

Unharvested Cropland × Year ^a^ 8 -1140.55 187.71 0.00

Harvested Cropland 6 -1245.27 393.10 0.00

Unharvested Cropland 6 -1247.72 398.02 0.00

Random Effects Only 3 -1262.02 420.56 0.00

Baseline Model 5 -1260.77 422.09 0.00

Avian Predators Corvid Abundance ^a^ 6 -558.30 0.00 0.82

Raptor Abundance 6 -560.45 4.30 0.10

Baseline Model 5 -561.60 4.56 0.08

Random Effects Only 3 -568.21 13.72 0.00

Competition Turkey Abundance 6 -559.89 0.00 0.66

Baseline Model 5 -561.60 1.37 0.33

Random Effects Only 3 -568.21 10.54 0.00

Climate Minimum temperature (Breeding) ^a^ 6 -1309.28 0.00 0.77

Minimum temperature (Winter) ^a^ 6 -1310.77 2.98 0.17

Minimum temperature (Brood-rearing) ^a^ 6 -1311.95 5.33 0.05

Precipitation (Brood-rearing) ^a^ 6 -1314.27 9.99 0.01

Precipitation (Breeding) 6 -1315.16 11.75 0.00

Random Effects Only 3 -1318.91 13.21 0.00

Baseline Model 5 -1317.47 14.37 0.00

Precipitation (Winter) 6 -1317.39 16.22 0.00

^a^ These models were carried forward to the second step of the modeling process.

Column Abbreviations: *LL* = Log(*Likelihood*); *K* = number of estimated parameters; AIC*_c_* = Akaike’s Information Criterion with second-order bias correction; ΔAIC*_c_* = difference (Δ) in AIC_c_ between best approximating model and model of interest; *w* = model probability.

**Table S8.** Model evaluation of generalized linear mixed effects models on a joint index of ring-necked pheasant (*Phasianus colchicus*) abundance in the North Central Region of California.

Model Covariate *K* *LL* ΔAIC*_c_* *w R^2^c*

HC × Year + UC × Year + Corv + TMIN(Breed) 12 -436.14 0.00 0.29 0.67

HC × Year + UC × Year + Corv 11 -437.37 0.39 0.24 0.66

HC × Year + UC × Year + Corv + TMIN(Win) 12 -436.79 1.31 0.15 0.66

HC × Year + UC × Year + Corv + TMIN(Brood) 12 -437.04 1.81 0.12 0.66

HC × Year + UC × Year + Corv + PPT(Brood) 12 -437.20 2.12 0.10 0.66

HC × Year + Corv + TMIN(Breed) 10 -440.28 4.12 0.04 0.64

HC × Year + Corv + TMIN(Breed) + PPT(Brood) 11 -440.26 6.15 0.01 0.64

HC × Year + Corv 9 -442.52 6.53 0.01 0.62

HC × Year + Corv + TMIN(Win) 10 -441.79 7.13 0.01 0.62

HC × Year + Corv + TMIN(Brood) 10 -442.11 7.78 0.01 0.62

HC × Year + Corv + PPT(Brood) 10 -442.41 8.37 0.00 0.62

HC × Year + Corv + TMIN(Win) + PPT(Brood) 11 -441.70 9.03 0.00 0.62

HC × Year + Corv + TMIN(Brood) + PPT(Brood) 11 -441.98 9.59 0.00 0.62

UC × Year + Corv 9 -456.34 34.16 0.00 0.68

UC × Year + Corv + TMIN(Win) 10 -455.38 34.31 0.00 0.67

UC × Year + Corv + TMIN(Brood) 10 -455.42 34.40 0.00 0.67

UC × Year + Corv + TMIN(Breed) 10 -455.62 34.80 0.00 0.66

UC × Year + Corv + PPT(Brood) 10 -456.33 36.22 0.00 0.68

UC × Year + Corv + TMIN(Win) + PPT(Brood) 11 -455.38 36.39 0.00 0.67

UC × Year + Corv + TMIN(Brood) + PPT(Brood) 11 -455.41 36.46 0.00 0.67

UC × Year + Corv + TMIN(Breed) + PPT(Brood) 11 -455.62 36.88 0.00 0.66

Corv + TMIN(Brood) 7 -501.76 120.89 0.00 0.71

Corv + TMIN(Brood) + PPT(Brood) 8 -500.80 121.02 0.00 0.72

Corv + TMIN(Breed) + PPT(Brood) 8 -502.73 124.87 0.00 0.72

Corv + TMIN(Breed) 7 -504.52 126.40 0.00 0.71

Corv + TMIN(Win) + PPT(Brood) 8 -503.68 126.79 0.00 0.71

Corv + PPT(Brood) 7 -505.06 127.49 0.00 0.69

Corv + TMIN(Win) 7 -505.30 127.97 0.00 0.70

Corv 6 -506.86 129.03 0.00 0.69

HC × Year + UC × Year + TMIN(Win) + PPT(Brood) 12 -1037.56 1202.53 0.00 0.86

HC × Year + UC × Year + PPT(Brood) 11 -1038.78 1202.92 0.00 0.87

HC × Year + UC × Year + TMIN(Brood) + PPT(Brood) 12 -1038.12 1203.64 0.00 0.86

HC × Year + UC × Year + TMIN(Breed) + PPT(Brood) 12 -1038.50 1204.40 0.00 0.86

HC × Year + TMIN(Win) + PPT(Brood) 10 -1041.87 1207.07 0.00 0.88

HC × Year + UC × Year + TMIN(Win) 11 -1041.03 1207.42 0.00 0.86

HC × Year + PPT(Brood) 9 -1043.30 1207.91 0.00 0.88

HC × Year + UC × Year 10 -1042.33 1207.99 0.00 0.86

HC × Year + TMIN(Brood) + PPT(Brood) 10 -1042.53 1208.39 0.00 0.88

HC × Year + UC × Year + TMIN(Brood) 11 -1041.57 1208.50 0.00 0.85

HC × Year + TMIN(Breed) + PPT(Brood) 10 -1042.94 1209.21 0.00 0.88

HC × Year + UC × Year + TMIN(Breed) 11 -1041.98 1209.32 0.00 0.85

HC × Year + TMIN(Win) 9 -1045.18 1211.66 0.00 0.87

HC × Year 8 -1046.69 1212.66 0.00 0.88

HC × Year + TMIN(Brood) 9 -1045.86 1213.02 0.00 0.87

HC × Year + TMIN(Breed) + + 9 -1046.30 1213.91 0.00 0.87

UC × Year + TMIN(Brood) + PPT(Brood) 10 -1136.07 1395.48 0.00 0.67

UC × Year + TMIN(Brood) 9 -1138.22 1397.75 0.00 0.66

UC × Year + TMIN(Win) + PPT(Brood) 10 -1137.22 1397.77 0.00 0.68

UC × Year + PPT(Brood) 9 -1138.28 1397.86 0.00 0.70

UC × Year + TMIN(Breed) + PPT(Brood) 10 -1137.84 1399.01 0.00 0.68

UC × Year + TMIN(Win) + + 9 -1139.41 1400.12 0.00 0.67

UC × Year 8 -1140.55 1400.36 0.00 0.69

UC × Year + TMIN(Breed) 9 -1140.05 1401.41 0.00 0.67

Random Effects Only 3 -1262.02 1633.22 0.00 0.64

Baseline Model 5 -1260.77 1634.75 0.00 0.64

PPT(Brood) 6 -1255.19 1625.60 0.00 0.65

TMIN(Brood) 6 -1254.39 1624.00 0.00 0.71

TMIN(Win) 6 -1250.66 1616.56 0.00 0.70

TMIN(Breed) 6 -1248.09 1611.40 0.00 0.74

TMIN(Brood) + PPT(Brood) 7 -1249.41 1616.07 0.00 0.72

TMIN(Win) + PPT(Brood) 7 -1245.68 1608.61 0.00 0.71

TMIN(Breed) + PPT(Brood) 7 -1243.57 1604.38 0.00 0.75

Column Abbreviations: *LL* = Log(*Likelihood*); *K* = number of estimated parameters; AIC*_c_* = Akaike’s Information Criterion with second-order bias correction; ΔAIC*_c_* = difference (Δ) in AIC_c_ between best approximating model and model of interest; *w* = model probability.

**Table S9.** Model evaluation of variable reduction analysis using generalized linear mixed effects models on a joint index of ring-necked pheasants (*Phasianus colchicus*) abundance in the Bay Delta Region of California.

Group Model Covariate *K* *LL* ΔAIC*_c_* *w*

Land Use Harvested Cropland × Year ^a^ 8 -1273.94 0.00 1.00

Unharvested Cropland × Year ^a^ 8 -1367.00 186.13 0.00

Unharvested Cropland 6 -1425.88 299.85 0.00

Harvested Cropland 6 -1538.37 524.83 0.00

Random Effects Only 3 -1565.94 573.92 0.00

Baseline Model 5 -1565.25 576.58 0.00

Avian Predators Corvid Abundance ^a^ 6 -665.98 0.00 1.00

Raptor Abundance ^a^ 6 -691.76 51.55 0.00

Random Effects Only 3 -700.44 62.82 0.00

Baseline Model 5 -699.53 65.05 0.00

Competition Turkey Abundance ^a^ 6 -695.36 0.00 0.85

Random Effects Only 3 -700.44 4.07 0.11

Baseline Model 5 -699.53 6.30 0.04

Climate Minimum temperature (Breeding) ^a^ 6 -1537.09 0.00 1.00

Minimum temperature (Brood-rearing) ^a^ 6 -1573.09 72.01 0.00

Minimum temperature (Winter) ^a^ 6 -1591.14 108.10 0.00

Precipitation (Brood-rearing) 6 -1613.99 153.81 0.00

Random Effects Only 3 -1617.61 155.00 0.00

Precipitation (Breeding) 6 -1615.26 156.35 0.00

Baseline Model 5 -1617.23 158.28 0.00

Precipitation (Winter) 6 -1616.97 159.77 0.00

^a^ These models were carried forward to the second step of the modeling process.

Column Abbreviations: *LL* = Log(*Likelihood*); *K* = number of estimated parameters; AIC*_c_* = Akaike’s Information Criterion with second-order bias correction; ΔAIC*_c_* = difference (Δ) in AIC_c_ between best approximating model and model of interest; *w* = model probability.

**Table S10.** Model evaluation of generalized linear mixed effects models on a joint index of ring-necked pheasant (*Phasianus colchicus*) abundance in the Bay Delta Region of California.

Model Covariate *K* *LL* ΔAIC*_c_* *w R^2^c*

Corv + HC × Year 9 -560.83 0.00 0.08 0.65

HC × Year 8 -562.09 0.46 0.06 0.66

Corv + TMIN(Win) + HC × Year 10 -560.14 0.69 0.06 0.65

Corv + TMIN(Brood) + HC × Year 10 -560.21 0.83 0.05 0.65

TMIN(Win) + HC × Year 9 -561.42 1.18 0.04 0.66

TMIN(Brood) + HC × Year 9 -561.51 1.36 0.04 0.65

Corv + TMIN(Brood) + TMIN(Win) + HC × Year 11 -559.70 1.88 0.03 0.65

Corv + Turk + HC × Year 10 -560.77 1.96 0.03 0.65

Rapt + HC × Year 9 -561.84 2.02 0.03 0.66

Corv + TMIN(Breed) + HC × Year 10 -560.83 2.06 0.03 0.65

Corv + Rapt + HC × Year 10 -560.83 2.06 0.03 0.65

TMIN(Brood) + TMIN(Win) + HC × Year 10 -561.01 2.42 0.02 0.65

Turk + HC × Year 9 -562.08 2.50 0.02 0.66

TMIN(Breed) + HC × Year 9 -562.08 2.51 0.02 0.66

Corv + TMIN(Breed) + TMIN(Win) + HC × Year 11 -560.04 2.56 0.02 0.66

Corv + Turk + TMIN(Win) + HC × Year 11 -560.09 2.66 0.02 0.65

Corv + TMIN(Breed) + TMIN(Brood) + HC × Year 11 -560.14 2.75 0.02 0.65

Corv + Rapt + TMIN(Win) + HC × Year 11 -560.14 2.76 0.02 0.65

Corv + Turk + TMIN(Brood) + HC × Year 11 -560.15 2.77 0.02 0.65

Rapt + TMIN(Win) + HC × Year 10 -561.18 2.77 0.02 0.66

Corv + Rapt + TMIN(Brood) + HC × Year 11 -560.21 2.90 0.02 0.65

Rapt + TMIN(Brood) + HC × Year 10 -561.25 2.90 0.02 0.65

TMIN(Breed) + TMIN(Win) + HC × Year 10 -561.29 2.99 0.02 0.66

Turk + TMIN(Win) + HC × Year 10 -561.41 3.23 0.02 0.66

TMIN(Breed) + TMIN(Brood) + HC × Year 10 -561.42 3.25 0.02 0.66

Turk + TMIN(Brood) + HC × Year 10 -561.50 3.40 0.01 0.65

Corv + HC × Year + UC × Year 11 -560.51 3.49 0.01 0.65

TMIN(Breed) + TMIN(Brood) + TMIN(Win) + HC × Year 11 -560.74 3.95 0.01 0.66

Rapt + TMIN(Brood) + TMIN(Win) + HC × Year 11 -560.76 4.00 0.01 0.65

Corv + Turk + TMIN(Breed) + HC × Year 11 -560.77 4.03 0.01 0.65

Corv + Rapt + Turk + HC × Year 11 -560.77 4.03 0.01 0.65

Rapt + Turk + HC × Year 10 -561.82 4.04 0.01 0.66

Rapt + TMIN(Breed) + HC × Year 10 -561.83 4.08 0.01 0.66

HC × Year + UC × Year 10 -561.85 4.11 0.01 0.66

Corv + Rapt + TMIN(Breed) + HC × Year 11 -560.82 4.13 0.01 0.65

Corv + TMIN(Win) + HC × Year + UC × Year 12 -559.83 4.22 0.01 0.65

Corv + TMIN(Brood) + HC × Year + UC × Year 12 -559.90 4.35 0.01 0.65

Turk + TMIN(Brood) + TMIN(Win) + HC × Year 11 -561.00 4.47 0.01 0.65

Turk + TMIN(Breed) + HC × Year 10 -562.07 4.56 0.01 0.66

Rapt + TMIN(Breed) + TMIN(Win) + HC × Year 11 -561.08 4.64 0.01 0.66

Rapt + Turk + TMIN(Win) + HC × Year 11 -561.16 4.80 0.01 0.66

TMIN(Win) + HC × Year + UC × Year 11 -561.17 4.82 0.01 0.66

Rapt + TMIN(Breed) + TMIN(Brood) + HC × Year 11 -561.17 4.83 0.01 0.65

Rapt + Turk + TMIN(Brood) + HC × Year 11 -561.22 4.92 0.01 0.65

TMIN(Brood) + HC × Year + UC × Year 11 -561.27 5.02 0.01 0.65

Turk + TMIN(Breed) + TMIN(Win) + HC × Year 11 -561.29 5.05 0.01 0.66

Turk + TMIN(Breed) + TMIN(Brood) + HC × Year 11 -561.41 5.30 0.01 0.66

Corv + TMIN(Breed) + HC × Year + UC × Year 12 -560.50 5.56 0.00 0.65

Corv + Turk + HC × Year + UC × Year 12 -560.50 5.56 0.00 0.65

Corv + Rapt + HC × Year + UC × Year 12 -560.50 5.56 0.00 0.65

Rapt + HC × Year + UC × Year 11 -561.61 5.70 0.00 0.66

TMIN(Brood) + TMIN(Win) + HC × Year + UC × Year 12 -560.73 6.02 0.00 0.65

Rapt + Turk + TMIN(Breed) + HC × Year 11 -561.81 6.11 0.00 0.66

TMIN(Breed) + HC × Year + UC × Year 11 -561.84 6.16 0.00 0.66

Turk + HC × Year + UC × Year 11 -561.85 6.18 0.00 0.66

Rapt + TMIN(Win) + HC × Year + UC × Year 12 -560.94 6.43 0.00 0.66

Rapt + TMIN(Brood) + HC × Year + UC × Year 12 -561.01 6.58 0.00 0.65

TMIN(Breed) + TMIN(Win) + HC × Year + UC × Year 12 -561.05 6.66 0.00 0.66

Turk + TMIN(Win) + HC × Year + UC × Year 12 -561.17 6.89 0.00 0.66

TMIN(Breed) + TMIN(Brood) + HC × Year + UC × Year 12 -561.18 6.92 0.00 0.65

Turk + TMIN(Brood) + HC × Year + UC × Year 12 -561.27 7.09 0.00 0.65

Rapt + TMIN(Breed) + HC × Year + UC × Year 12 -561.60 7.77 0.00 0.66

Rapt + Turk + HC × Year + UC × Year 12 -561.61 7.78 0.00 0.66

Turk + TMIN(Breed) + HC × Year + UC × Year 12 -561.84 8.23 0.00 0.66

Corv + Turk + UC × Year 10 -585.42 51.25 0.00 0.70

Corv + Turk + TMIN(Win) + UC × Year 11 -585.13 52.73 0.00 0.70

Corv + Rapt + Turk + UC × Year 11 -585.17 52.82 0.00 0.70

Corv + Turk + TMIN(Brood) + UC × Year 11 -585.18 52.84 0.00 0.69

Corv + Turk + TMIN(Breed) + UC × Year 11 -585.36 53.20 0.00 0.70

Corv + UC × Year 9 -588.12 54.59 0.00 0.69

Corv + Rapt + UC × Year 10 -587.70 55.80 0.00 0.69

Corv + TMIN(Win) + UC × Year 10 -587.84 56.08 0.00 0.69

Corv + TMIN(Brood) + UC × Year 10 -587.93 56.27 0.00 0.68

Corv + TMIN(Breed) + UC × Year 10 -587.96 56.34 0.00 0.69

Corv + Rapt + TMIN(Win) + UC × Year 11 -587.40 57.27 0.00 0.69

Corv + Rapt + TMIN(Breed) + UC × Year 11 -587.49 57.46 0.00 0.69

Corv + Rapt + TMIN(Brood) + UC × Year 11 -587.51 57.49 0.00 0.68

Corv + TMIN(Breed) + TMIN(Win) + UC × Year 11 -587.51 57.50 0.00 0.70

Corv + TMIN(Breed) + TMIN(Brood) + UC × Year 11 -587.66 57.80 0.00 0.69

Corv + TMIN(Brood) + TMIN(Win) + UC × Year 11 -587.70 57.87 0.00 0.68

Turk + UC × Year 9 -591.29 60.93 0.00 0.69

Rapt + Turk + UC × Year 10 -590.81 62.04 0.00 0.69

Turk + TMIN(Win) + UC × Year 10 -590.99 62.40 0.00 0.69

Turk + TMIN(Brood) + UC × Year 10 -591.10 62.61 0.00 0.69

Turk + TMIN(Breed) + UC × Year 10 -591.24 62.89 0.00 0.69

UC × Year 8 -593.58 63.44 0.00 0.68

Rapt + Turk + TMIN(Win) + UC × Year 11 -590.53 63.53 0.00 0.69

Rapt + Turk + TMIN(Brood) + UC × Year 11 -590.61 63.70 0.00 0.69

Rapt + Turk + TMIN(Breed) + UC × Year 11 -590.78 64.04 0.00 0.69

Turk + TMIN(Breed) + TMIN(Win) + UC × Year 11 -590.84 64.16 0.00 0.70

Turk + TMIN(Brood) + TMIN(Win) + UC × Year 11 -590.86 64.19 0.00 0.69

Turk + TMIN(Breed) + TMIN(Brood) + UC × Year 11 -590.99 64.45 0.00 0.69

TMIN(Win) + UC × Year 9 -593.29 64.92 0.00 0.68

Rapt + UC × Year 9 -593.30 64.95 0.00 0.68

TMIN(Brood) + UC × Year 9 -593.43 65.19 0.00 0.68

TMIN(Breed) + UC × Year 9 -593.45 65.24 0.00 0.69

TMIN(Breed) + TMIN(Win) + UC × Year 10 -593.00 66.42 0.00 0.69

Rapt + TMIN(Win) + UC × Year 10 -593.02 66.45 0.00 0.68

Rapt + TMIN(Brood) + UC × Year 10 -593.14 66.69 0.00 0.68

TMIN(Brood) + TMIN(Win) + UC × Year 10 -593.18 66.77 0.00 0.68

Rapt + TMIN(Breed) + UC × Year 10 -593.19 66.80 0.00 0.69

TMIN(Breed) + TMIN(Brood) + UC × Year 10 -593.21 66.82 0.00 0.69

Rapt + TMIN(Breed) + TMIN(Win) + UC × Year 11 -592.77 68.02 0.00 0.69

TMIN(Breed) + TMIN(Brood) + TMIN(Win) + UC × Year 11 -592.80 68.09 0.00 0.69

Rapt + TMIN(Brood) + TMIN(Win) + UC × Year 11 -592.91 68.30 0.00 0.68

Rapt + TMIN(Breed) + TMIN(Brood) + UC × Year 11 -592.95 68.38 0.00 0.69

Corv + TMIN(Breed) + TMIN(Brood) 8 -645.14 166.56 0.00 0.72

Corv + Turk + TMIN(Breed) + TMIN(Brood) 9 -644.84 168.01 0.00 0.71

Corv + Rapt + TMIN(Breed) + TMIN(Brood) 9 -644.94 168.22 0.00 0.72

Corv + TMIN(Breed) + TMIN(Brood) + TMIN(Win) 9 -645.07 168.49 0.00 0.71

Corv + TMIN(Breed) 7 -647.28 168.80 0.00 0.71

Corv + Turk + TMIN(Breed) 8 -646.84 169.97 0.00 0.71

Corv + Rapt + TMIN(Breed) 8 -647.07 170.42 0.00 0.71

Corv + TMIN(Breed) + TMIN(Win) 8 -647.10 170.49 0.00 0.71

Corv + Rapt + Turk + TMIN(Breed) 9 -646.56 171.47 0.00 0.71

Corv + Turk + TMIN(Breed) + TMIN(Win) 9 -646.69 171.73 0.00 0.70

Corv + Rapt + TMIN(Breed) + TMIN(Win) 9 -646.90 172.14 0.00 0.71

Corv + TMIN(Brood) + TMIN(Win) 8 -654.50 185.29 0.00 0.64

Corv + Turk + TMIN(Brood) + TMIN(Win) 9 -654.07 186.48 0.00 0.63

Corv + TMIN(Brood) 7 -656.43 187.09 0.00 0.64

Corv + Rapt + TMIN(Brood) + TMIN(Win) 9 -654.47 187.28 0.00 0.64

Corv + Turk + TMIN(Brood) 8 -655.86 188.01 0.00 0.64

Corv + Rapt + TMIN(Brood) 8 -656.38 189.05 0.00 0.64

Corv + Rapt + Turk + TMIN(Brood) 9 -655.78 189.91 0.00 0.64

Corv + TMIN(Win) 7 -661.35 196.93 0.00 0.60

Corv + Turk + TMIN(Win) 8 -660.56 197.41 0.00 0.59

Corv + Rapt + TMIN(Win) 8 -661.33 198.95 0.00 0.60

Corv + Rapt + Turk + TMIN(Win) 9 -660.52 199.37 0.00 0.59

Rapt + TMIN(Breed) + TMIN(Brood) 8 -663.55 203.38 0.00 0.72

Rapt + Turk + TMIN(Breed) + TMIN(Brood) 9 -662.71 203.76 0.00 0.71

Corv + Turk 7 -664.80 203.82 0.00 0.59

Corv 6 -665.98 204.15 0.00 0.59

Rapt + TMIN(Breed) + TMIN(Brood) + TMIN(Win) 9 -663.27 204.89 0.00 0.71

Corv + Rapt + Turk 8 -664.73 205.74 0.00 0.59

Corv + Rapt 7 -665.96 206.15 0.00 0.59

Turk + TMIN(Breed) + TMIN(Brood) 8 -665.75 207.79 0.00 0.71

TMIN(Breed) + TMIN(Brood) 7 -667.16 208.56 0.00 0.72

Rapt + Turk + TMIN(Breed) 8 -666.21 208.70 0.00 0.70

Rapt + TMIN(Breed) 7 -667.39 209.01 0.00 0.71

Turk + TMIN(Breed) + TMIN(Brood) + TMIN(Win) 9 -665.57 209.49 0.00 0.71

Rapt + Turk + TMIN(Breed) + TMIN(Win) 9 -665.73 209.81 0.00 0.70

Rapt + TMIN(Breed) + TMIN(Win) 8 -666.80 209.89 0.00 0.70

TMIN(Breed) + TMIN(Brood) + TMIN(Win) 8 -666.92 210.13 0.00 0.71

Turk + TMIN(Breed) 7 -669.49 213.20 0.00 0.70

Turk + TMIN(Breed) + TMIN(Win) 8 -669.06 214.41 0.00 0.70

TMIN(Breed) 6 -671.38 214.96 0.00 0.71

TMIN(Breed) + TMIN(Win) 7 -670.83 215.90 0.00 0.70

Rapt + TMIN(Brood) + TMIN(Win) 8 -673.96 224.21 0.00 0.63

Rapt + Turk + TMIN(Brood) + TMIN(Win) 9 -672.97 224.28 0.00 0.62

Rapt + Turk + TMIN(Brood) 8 -675.80 227.88 0.00 0.62

Rapt + TMIN(Brood) 7 -677.07 228.37 0.00 0.63

Turk + TMIN(Brood) + TMIN(Win) 8 -677.49 231.26 0.00 0.62

TMIN(Brood) + TMIN(Win) 7 -679.28 232.79 0.00 0.62

Turk + TMIN(Brood) 7 -680.41 235.05 0.00 0.62

TMIN(Brood) 6 -682.60 237.39 0.00 0.62

Rapt + Turk + TMIN(Win) 8 -682.46 241.20 0.00 0.56

Rapt + TMIN(Win) 7 -684.16 242.55 0.00 0.56

Turk + TMIN(Win) 7 -688.13 250.49 0.00 0.54

Rapt + Turk 7 -689.21 252.66 0.00 0.54

TMIN(Win) 6 -691.08 254.34 0.00 0.55

Rapt 6 -691.76 255.71 0.00 0.54

Turk 6 -695.36 262.90 0.00 0.53

Random Effects Only 3 -700.44 266.97 0.00 0.53

Baseline Model 5 -699.53 269.20 0.00 0.53

Column Abbreviations: *LL* = Log(*Likelihood*); *K* = number of estimated parameters; AIC*_c_* = Akaike’s Information Criterion with second-order bias correction; ΔAIC*_c_* = difference (Δ) in AIC_c_ between best approximating model and model of interest; *w* = model probability.

**Table S11.** Model evaluation of variable reduction analysis using generalized linear mixed effects models on a joint index of ring-necked pheasants (*Phasianus colchicus*) abundance in the Central Region of California.

Group Model Covariate *K* *LL* ΔAIC*c* *w*

Land Use Harvested Cropland × Year ^a^ 8 -1214.13 0.00 1.00

Unharvested Cropland × Year ^a^ 8 -1341.95 255.63 0.00

Harvested Cropland 6 -1449.21 466.12 0.00

Unharvested Cropland 6 -1458.66 485.01 0.00

Baseline Model 5 -1519.29 604.27 0.00

Random Effects Only 3 -1522.15 605.96 0.00

Avian Predators Raptor Abundance ^a^ 6 -931.70 0.00 1.00

Corvid Abundance ^a^ 6 -942.22 21.03 0.00

Random Effects Only 3 -963.80 58.13 0.00

Baseline Model 5 -962.81 60.19 0.00

Competition Random Effects Only 3 -963.80 0.00 0.62

Baseline Model 5 -962.81 2.06 0.22

Turkey Abundance 6 -962.14 2.74 0.16

Climate Minimum temperature (Breeding) ^a^ 6 -1505.10 0.00 1.00

Minimum temperature (Brood-rearing) ^a^ 6 -1538.72 67.25 0.00

Minimum temperature (Winter) ^a^ 6 -1552.12 94.05 0.00

Precipitation (Brood-rearing) ^a^ 6 -1559.03 107.87 0.00

Baseline Model 5 -1570.49 128.77 0.00

Random Effects Only 3 -1572.70 129.17 0.00

Precipitation (Breeding) 6 -1570.34 130.50 0.00

Precipitation (Winter) 6 -1570.48 130.76 0.00

^a^ These models were carried forward to the second step of the modeling process.

Column Abbreviations: *LL* = Log(*Likelihood*); *K* = number of estimated parameters; AIC*_c_* = Akaike’s Information Criterion with second-order bias correction; ΔAIC*_c_* = difference (Δ) in AIC_c_ between best approximating model and model of interest; *w* = model probability.

**Table S12.** Model evaluation of generalized linear mixed effects models on a joint index of ring-necked pheasant (*Phasianus colchicus*) abundance in the Central Region of California.

Model Covariate *K* *LL* ΔAIC*_c_* *w R^2^c*

TMIN(Breed) + PPT(Brood) + HC × Year + UC × Year 12 -771.10 0.00 0.27 0.88

TMIN(Breed) + PPT(Brood) + HC × Year 10 -773.55 0.79 0.19 0.89

TMIN(Breed) + PPT(Brood) + TMIN(Win) + HC × Year 11 -772.90 1.55 0.13 0.90

Rapt + TMIN(Breed) + PPT(Brood) + HC × Year 11 -773.25 2.25 0.09 0.89

Corv + TMIN(Breed) + PPT(Brood) + HC × Year 11 -773.49 2.73 0.07 0.89

PPT(Brood) + HC × Year + UC × Year 11 -773.64 3.02 0.06 0.86

PPT(Brood) + TMIN(Win) + HC × Year + UC × Year 12 -773.28 4.35 0.03 0.87

Rapt + PPT(Brood) + HC × Year + UC × Year 12 -773.29 4.38 0.03 0.87

TMIN(Brood) + PPT(Brood) + HC × Year + UC × Year 12 -773.51 4.82 0.02 0.87

Corv + PPT(Brood) + HC × Year + UC × Year 12 -773.64 5.07 0.02 0.86

PPT(Brood) + HC × Year 9 -777.06 5.78 0.02 0.87

Rapt + PPT(Brood) + HC × Year 10 -776.68 7.06 0.01 0.87

PPT(Brood) + TMIN(Win) + HC × Year 10 -776.80 7.30 0.01 0.87

TMIN(Brood) + PPT(Brood) + HC × Year 10 -776.89 7.48 0.01 0.87

Corv + PPT(Brood) + HC × Year 10 -776.94 7.57 0.01 0.87

Rapt + PPT(Brood) + TMIN(Win) + HC × Year 11 -776.38 8.50 0.00 0.87

TMIN(Breed) + HC × Year + UC × Year 11 -776.45 8.64 0.00 0.87

Rapt + TMIN(Brood) + PPT(Brood) + HC × Year 11 -776.54 8.83 0.00 0.87

Rapt + Corv + PPT(Brood) + HC × Year 11 -776.61 8.97 0.00 0.87

TMIN(Brood) + PPT(Brood) + TMIN(Win) + HC × Year 11 -776.61 8.97 0.00 0.87

Corv + PPT(Brood) + TMIN(Win) + HC × Year 11 -776.68 9.10 0.00 0.87

TMIN(Breed) + TMIN(Win) + HC × Year + UC × Year 12 -775.70 9.19 0.00 0.88

Corv + TMIN(Brood) + PPT(Brood) + HC × Year 11 -776.76 9.26 0.00 0.87

TMIN(Breed) + HC × Year 9 -779.06 9.77 0.00 0.88

TMIN(Breed) + TMIN(Win) + HC × Year 10 -778.36 10.41 0.00 0.89

Rapt + TMIN(Breed) + HC × Year + UC × Year 12 -776.37 10.55 0.00 0.87

Corv + TMIN(Breed) + HC × Year + UC × Year 12 -776.43 10.67 0.00 0.87

HC × Year + UC × Year 10 -778.56 10.81 0.00 0.85

Rapt + TMIN(Breed) + HC × Year 10 -778.97 11.63 0.00 0.88

Corv + TMIN(Breed) + HC × Year 10 -779.02 11.74 0.00 0.88

TMIN(Win) + HC × Year + UC × Year 11 -778.13 12.01 0.00 0.85

Rapt + TMIN(Breed) + TMIN(Win) + HC × Year 11 -778.24 12.22 0.00 0.89

Corv + TMIN(Breed) + TMIN(Win) + HC × Year 11 -778.33 12.41 0.00 0.89

TMIN(Brood) + HC × Year + UC × Year 11 -778.43 12.60 0.00 0.85

Rapt + HC × Year + UC × Year 11 -778.43 12.61 0.00 0.85

Corv + HC × Year + UC × Year 11 -778.55 12.85 0.00 0.85

Rapt + Corv + TMIN(Breed) + HC × Year 11 -778.95 13.64 0.00 0.88

Rapt + TMIN(Win) + HC × Year + UC × Year 12 -777.97 13.74 0.00 0.86

TMIN(Brood) + TMIN(Win) + HC × Year + UC × Year 12 -777.98 13.75 0.00 0.86

HC × Year 8 -782.09 13.79 0.00 0.85

Corv + TMIN(Win) + HC × Year + UC × Year 12 -778.13 14.05 0.00 0.85

Rapt + TMIN(Brood) + HC × Year + UC × Year 12 -778.31 14.42 0.00 0.85

Rapt + Corv + HC × Year + UC × Year 12 -778.42 14.64 0.00 0.85

Corv + TMIN(Brood) + HC × Year + UC × Year 12 -778.42 14.64 0.00 0.85

TMIN(Win) + HC × Year 9 -781.77 15.19 0.00 0.86

TMIN(Brood) + HC × Year 9 -781.92 15.48 0.00 0.85

Rapt + HC × Year 9 -781.94 15.54 0.00 0.85

Corv + HC × Year 9 -782.00 15.65 0.00 0.85

TMIN(Brood) + TMIN(Win) + HC × Year 10 -781.58 16.85 0.00 0.86

Rapt + TMIN(Win) + HC × Year 10 -781.60 16.89 0.00 0.86

Corv + TMIN(Win) + HC × Year 10 -781.69 17.07 0.00 0.86

Rapt + TMIN(Brood) + HC × Year 10 -781.79 17.28 0.00 0.85

Corv + TMIN(Brood) + HC × Year 10 -781.82 17.34 0.00 0.86

Rapt + Corv + HC × Year 10 -781.88 17.46 0.00 0.85

Rapt + TMIN(Brood) + TMIN(Win) + HC × Year 11 -781.43 18.60 0.00 0.86

Corv + TMIN(Brood) + TMIN(Win) + HC × Year 11 -781.49 18.72 0.00 0.86

Rapt + Corv + TMIN(Win) + HC × Year 11 -781.54 18.83 0.00 0.86

Rapt + Corv + TMIN(Brood) + HC × Year 11 -781.72 19.19 0.00 0.86

Rapt + TMIN(Breed) + PPT(Brood) + UC × Year 11 -835.89 127.52 0.00 0.72

TMIN(Breed) + PPT(Brood) + UC × Year 10 -837.71 129.11 0.00 0.72

Corv + TMIN(Breed) + PPT(Brood) + UC × Year 11 -837.67 131.07 0.00 0.72

TMIN(Breed) + PPT(Brood) + TMIN(Win) + UC × Year 11 -837.69 131.13 0.00 0.72

Rapt + TMIN(Breed) + UC × Year 10 -839.20 132.10 0.00 0.71

TMIN(Breed) + UC × Year 9 -840.57 132.78 0.00 0.71

Rapt + Corv + TMIN(Breed) + UC × Year 11 -839.04 133.81 0.00 0.71

Rapt + TMIN(Breed) + TMIN(Win) + UC × Year 11 -839.15 134.04 0.00 0.71

Corv + TMIN(Breed) + UC × Year 10 -840.51 134.71 0.00 0.71

TMIN(Breed) + TMIN(Win) + UC × Year 10 -840.54 134.77 0.00 0.71

Rapt + PPT(Brood) + UC × Year 10 -841.06 135.82 0.00 0.65

Corv + TMIN(Breed) + TMIN(Win) + UC × Year 11 -840.47 136.69 0.00 0.71

Rapt + TMIN(Brood) + PPT(Brood) + UC × Year 11 -840.75 137.24 0.00 0.67

Rapt + Corv + PPT(Brood) + UC × Year 11 -840.96 137.67 0.00 0.65

Rapt + PPT(Brood) + TMIN(Win) + UC × Year 11 -841.05 137.85 0.00 0.66

PPT(Brood) + UC × Year 9 -843.29 138.23 0.00 0.65

Rapt + UC × Year 9 -843.92 139.49 0.00 0.65

TMIN(Brood) + PPT(Brood) + UC × Year 10 -842.91 139.51 0.00 0.66

PPT(Brood) + TMIN(Win) + UC × Year 10 -843.25 140.20 0.00 0.65

Corv + PPT(Brood) + UC × Year 10 -843.28 140.25 0.00 0.65

Rapt + TMIN(Brood) + UC × Year 10 -843.60 140.90 0.00 0.66

UC × Year 8 -845.65 140.92 0.00 0.64

Rapt + Corv + UC × Year 10 -843.81 141.32 0.00 0.65

TMIN(Brood) + PPT(Brood) + TMIN(Win) + UC × Year 11 -842.88 141.51 0.00 0.67

Rapt + TMIN(Win) + UC × Year 10 -843.92 141.53 0.00 0.65

Corv + TMIN(Brood) + PPT(Brood) + UC × Year 11 -842.90 141.55 0.00 0.66

TMIN(Brood) + UC × Year 9 -845.28 142.20 0.00 0.66

Corv + PPT(Brood) + TMIN(Win) + UC × Year 11 -843.24 142.23 0.00 0.65

Rapt + Corv + TMIN(Brood) + UC × Year 11 -843.51 142.77 0.00 0.66

Corv + UC × Year 9 -845.64 142.92 0.00 0.64

TMIN(Win) + UC × Year 9 -845.64 142.92 0.00 0.65

Rapt + TMIN(Brood) + TMIN(Win) + UC × Year 11 -843.60 142.95 0.00 0.66

Rapt + Corv + TMIN(Win) + UC × Year 11 -843.81 143.37 0.00 0.65

Corv + TMIN(Brood) + UC × Year 10 -845.26 144.22 0.00 0.66

TMIN(Brood) + TMIN(Win) + UC × Year 10 -845.27 144.23 0.00 0.66

Corv + TMIN(Win) + UC × Year 10 -845.62 144.93 0.00 0.64

Corv + TMIN(Brood) + TMIN(Win) + UC × Year 11 -845.26 146.25 0.00 0.66

Rapt + Corv + TMIN(Breed) + PPT(Brood) 9 -893.67 239.00 0.00 0.78

Rapt + TMIN(Breed) + PPT(Brood) 8 -898.48 246.58 0.00 0.77

Rapt + TMIN(Breed) + PPT(Brood) + TMIN(Win) 9 -898.06 247.78 0.00 0.77

Rapt + Corv + TMIN(Brood) + PPT(Brood) 9 -902.05 255.75 0.00 0.73

Rapt + Corv + TMIN(Breed) 8 -905.24 260.10 0.00 0.76

Rapt + Corv + TMIN(Breed) + TMIN(Win) 9 -904.81 261.28 0.00 0.76

Rapt + TMIN(Brood) + PPT(Brood) + TMIN(Win) 9 -908.13 267.92 0.00 0.72

Rapt + TMIN(Brood) + PPT(Brood) 8 -909.31 268.23 0.00 0.71

Rapt + Corv + PPT(Brood) + TMIN(Win) 9 -908.81 269.28 0.00 0.68

Rapt + TMIN(Breed) 7 -911.21 269.99 0.00 0.76

Corv + TMIN(Breed) + PPT(Brood) + TMIN(Win) 9 -909.17 270.00 0.00 0.79

Corv + TMIN(Breed) + PPT(Brood) 8 -910.32 270.25 0.00 0.79

Rapt + Corv + PPT(Brood) 8 -910.56 270.73 0.00 0.67

Rapt + TMIN(Breed) + TMIN(Win) 8 -910.82 271.24 0.00 0.76

Rapt + Corv + TMIN(Brood) + TMIN(Win) 9 -911.58 274.81 0.00 0.72

Rapt + Corv + TMIN(Brood) 8 -912.64 274.90 0.00 0.71

Rapt + PPT(Brood) + TMIN(Win) 8 -916.73 283.07 0.00 0.66

Rapt + PPT(Brood) 7 -918.57 284.71 0.00 0.65

Corv + TMIN(Brood) + PPT(Brood) + TMIN(Win) 9 -916.79 285.22 0.00 0.75

Corv + TMIN(Brood) + PPT(Brood) 8 -918.97 287.55 0.00 0.74

Corv + TMIN(Breed) + TMIN(Win) 8 -919.60 288.81 0.00 0.78

Corv + TMIN(Breed) 7 -920.68 288.93 0.00 0.78

Rapt + TMIN(Brood) + TMIN(Win) 8 -920.11 289.84 0.00 0.70

Rapt + TMIN(Brood) 7 -921.22 290.01 0.00 0.70

TMIN(Breed) + PPT(Brood) + TMIN(Win) 8 -920.48 290.56 0.00 0.79

Rapt + Corv + TMIN(Win) 8 -920.48 290.58 0.00 0.66

TMIN(Breed) + PPT(Brood) 7 -921.72 291.02 0.00 0.79

Rapt + Corv 7 -922.18 291.94 0.00 0.65

Corv + TMIN(Brood) + TMIN(Win) 8 -926.16 301.93 0.00 0.74

Corv + TMIN(Brood) 7 -928.20 303.98 0.00 0.73

Corv + PPT(Brood) + TMIN(Win) 8 -928.56 306.74 0.00 0.68

Rapt + TMIN(Win) 7 -929.91 307.40 0.00 0.64

Rapt 6 -931.70 308.95 0.00 0.63

Corv + PPT(Brood) 7 -931.99 311.56 0.00 0.67

TMIN(Breed) + TMIN(Win) 7 -932.33 312.24 0.00 0.78

TMIN(Breed) 6 -933.51 312.56 0.00 0.78

TMIN(Brood) + PPT(Brood) + TMIN(Win) 8 -932.14 313.89 0.00 0.73

TMIN(Brood) + PPT(Brood) 7 -934.77 317.11 0.00 0.73

Corv + TMIN(Win) 7 -938.92 325.41 0.00 0.66

Corv 6 -942.22 329.98 0.00 0.65

TMIN(Brood) + TMIN(Win) 7 -942.99 333.56 0.00 0.72

TMIN(Brood) 6 -945.48 336.51 0.00 0.71

PPT(Brood) + TMIN(Win) 7 -946.53 340.65 0.00 0.65

PPT(Brood) 6 -950.74 347.03 0.00 0.63

TMIN(Win) 6 -958.71 362.97 0.00 0.62

Random Effects Only 3 -963.80 367.08 0.00 0.60

Baseline model 5 -962.81 369.14 0.00 0.60

Column Abbreviations: *LL* = Log(*Likelihood*); *K* = number of estimated parameters; AIC*_c_* = Akaike’s Information Criterion with second-order bias correction; ΔAIC*_c_* = difference (Δ) in AIC_c_ between best approximating model and model of interest; *w* = model probability.

**Table S13.** Model evaluation of variable reduction analysis using generalized linear mixed effects models on a joint index of ring-necked pheasants (*Phasianus colchicus*) abundance in the South Coast Region of California.

Group Model Covariate *K* *LL* ΔAIC*_c_* *w*

Land Use Unharvested Cropland × Year ^a^ 8 -842.37 0.00 1.00

Harvested Cropland × Year ^a^ 8 -852.64 20.54 0.00

Unharvested Cropland 6 -881.54 74.25 0.00

Harvested Cropland 6 -890.34 91.86 0.00

Random Effects Only 3 -924.27 153.61 0.00

Baseline Model 5 -924.14 157.41 0.00

Avian Predators Corvid Abundance ^a^ 6 -265.19 0.00 0.96

Random Effects Only 3 -271.60 6.53 0.04

Baseline Model 5 -271.60 10.70 0.00

Raptor Abundance 6 -271.28 12.18 0.00

Competition Random Effects Only 3 -271.60 0.00 0.84

Baseline Model 5 -271.60 4.17 0.10

Turkey Abundance 6 -271.10 5.30 0.06

Climate Minimum temperature (Breeding) ^a^ 6 -947.29 0.00 1.00

Minimum temperature (Brood-rearing) ^a^ 6 -956.98 19.36 0.00

Minimum temperature (Winter) ^a^ 6 -962.00 29.41 0.00

Random Effects Only 3 -975.36 50.04 0.00

Precipitation (Winter) 6 -973.86 53.14 0.00

Baseline Model 5 -975.06 53.50 0.00

Precipitation (Brood-rearing) 6 -974.53 54.46 0.00

Precipitation (Breeding) 6 -974.97 55.35 0.00

^a^ These models were carried forward to the second step of the modeling process.

Column Abbreviations: *LL* = Log(*Likelihood*) ; *K* = number of estimated parameters; AIC*_c_* = Akaike’s Information Criterion with second-order bias correction; ΔAIC*_c_* = difference (Δ) in AIC_c_ between best approximating model and model of interest; *w* = model probability.

**Table S14.** Model evaluation of generalized linear mixed effects models on a joint index of ring-necked pheasant (*Phasianus colchicus*) abundance in the South Coast Region of California.

Model Covariate *K* *LL* ΔAIC*_c_* *w R^2^c*

Corv + UC × Year 9 -258.07 0.00 0.15 0.23

Corv + TMIN(Breed) + UC × Year 10 -257.10 0.27 0.13 0.25

TMIN(Breed) + UC × Year 9 -258.36 0.58 0.11 0.27

UC × Year 8 -259.85 1.37 0.08 0.24

Corv + TMIN(Breed) + HC × Year 10 -257.70 1.48 0.07 0.29

Corv + HC × Year 9 -258.84 1.54 0.07 0.26

Corv + TMIN(Brood) + UC × Year 10 -258.05 2.17 0.05 0.23

Corv + TMIN(Win) + UC × Year 10 -258.06 2.20 0.05 0.23

TMIN(Breed) + HC × Year 9 -259.40 2.67 0.04 0.32

Corv + TMIN(Breed) 7 -261.75 3.01 0.03 0.24

TMIN(Brood) + UC × Year 9 -259.78 3.42 0.03 0.23

TMIN(Win) + UC × Year 9 -259.85 3.56 0.03 0.24

Corv + TMIN(Brood) + HC × Year 10 -258.83 3.74 0.02 0.25

Corv + TMIN(Win) + HC × Year 10 -258.83 3.74 0.02 0.26

HC × Year 8 -261.19 4.06 0.02 0.27

Corv + HC × Year + UC × Year 11 -257.99 4.29 0.02 0.23

Corv + TMIN(Breed) + HC × Year + UC × Year 12 -256.98 4.51 0.02 0.26

TMIN(Breed) + HC × Year + UC × Year 11 -258.25 4.81 0.01 0.27

HC × Year + UC × Year 10 -259.76 5.59 0.01 0.23

TMIN(Brood) + HC × Year 9 -261.13 6.13 0.01 0.26

TMIN(Win) + HC × Year 9 -261.18 6.22 0.01 0.27

Corv + TMIN(Win) + HC × Year + UC × Year 12 -257.98 6.52 0.01 0.23

Corv + TMIN(Brood) + HC × Year + UC × Year 12 -257.98 6.52 0.01 0.23

TMIN(Breed) 6 -264.74 6.86 0.00 0.30

Corv + TMIN(Brood) 7 -263.77 7.05 0.00 0.22

TMIN(Brood) + HC × Year + UC × Year 11 -259.70 7.70 0.00 0.23

Corv 6 -265.19 7.75 0.00 0.18

TMIN(Win) + HC × Year + UC × Year 11 -259.76 7.82 0.00 0.23

Corv + TMIN(Win) 7 -264.57 8.66 0.00 0.21

Random Effects Only 3 -271.60 14.28 0.00 0.21

TMIN(Brood) 6 -269.45 16.26 0.00 0.28

Baseline Model 5 -271.60 18.45 0.00 0.21

TMIN(Win) 6 -270.64 18.65 0.00 0.27

Column Abbreviations: *LL* = Log(*Likelihood*); *K* = number of estimated parameters; AIC*_c_* = Akaike’s Information Criterion with second-order bias correction; ΔAIC*_c_* = difference (Δ) in AIC_c_ between best approximating model and model of interest; *w* = model probability.

**Table S15.** Model evaluation of variable reduction analysis using generalized linear mixed effects models on a joint index of ring-necked pheasants (*Phasianus colchicus*) abundance in the Inland Deserts Region of California.

Group Model Covariate *K* *LL* ΔAIC*_c_* *w*

Land Use Harvested Cropland × Year ^a^ 8 -680.18 0.00 1.00

Unharvested Cropland × Year ^a^ 8 -685.64 10.92 0.00

Harvested Cropland 6 -705.86 47.24 0.00

Unharvested Cropland 6 -716.39 68.30 0.00

Baseline Model 5 -721.43 76.34 0.00

Random Effects Only 3 -729.58 88.57 0.00

Avian Predators Random Effects Only 3 -406.32 0.00 0.75

Baseline Model 5 -406.18 3.84 0.11

Raptor Abundance 6 -405.25 4.06 0.10

Corvid Abundance 6 -406.16 5.88 0.04

Competition Random Effects Only 3 -406.32 0.00 0.77

Turkey Abundance 5 -406.18 3.84 0.11

Baseline Model 5 -406.18 3.84 0.11

Climate Minimum temperature (Breeding) ^a^ 6 -726.87 0.00 0.97

Minimum temperature (Winter) ^a^ 6 -730.50 7.26 0.03

Minimum temperature (Brood-rearing) ^a^ 6 -734.25 14.75 0.00

Precipitation (Winter) 6 -737.31 20.88 0.00

Baseline Model 5 -738.91 22.05 0.00

Precipitation (Brood-rearing) 6 -738.12 22.50 0.00

Precipitation (Breeding) 6 -738.46 23.19 0.00

Random Effects Only 3 -747.71 35.58 0.00

^a^ These models were carried forward to the second step of the modeling process.

Column Abbreviations: *LL* = Log(*Likelihood*); *K* = number of estimated parameters; AIC*_c_* = Akaike’s Information Criterion with second-order bias correction; ΔAIC*_c_* = difference (Δ) in AIC_c_ between best approximating model and model of interest; *w* = model probability.

**Table S16.** Model evaluation of generalized linear mixed effects models on a joint index of ring-necked pheasant (*Phasianus colchicus*) abundance in the Inland Desert Region of California.

Model Covariate *K* *LL* ΔAIC*_c_* *w R2c*

TMIN(Brood) + HC × Year + UC × Year 11 -670.97 0.00 0.80 0.41

HC × Year + UC × Year 10 -674.20 4.39 0.09 0.39

TMIN(Win) + HC × Year + UC × Year 11 -673.68 5.43 0.05 0.39

TMIN(Breed) + HC × Year + UC × Year 11 -674.06 6.19 0.04 0.40

TMIN(Brood) + HC × Year 9 -677.23 8.37 0.01 0.43

HC × Year 8 -680.18 12.22 0.00 0.42

TMIN(Brood) + UC × Year 9 -679.55 13.01 0.00 0.35

TMIN(Breed) + HC × Year 9 -680.16 14.24 0.00 0.43

TMIN(Win) + HC × Year 9 -680.17 14.26 0.00 0.42

UC × Year 8 -685.64 23.14 0.00 0.40

TMIN(Win) + UC × Year 9 -685.37 24.65 0.00 0.33

TMIN(Breed) + UC × Year 9 -685.63 25.18 0.00 0.42

TMIN(Breed) 6 -710.54 68.83 0.00 0.75

TMIN(Win) 6 -712.71 73.17 0.00 0.74

TMIN(Brood) 6 -718.85 85.45 0.00 0.68

Baseline Model 5 -721.43 88.56 0.00 0.36

Random Effects Only 3 -729.58 100.79 0.00 0.30

Column Abbreviations: *LL* = Log(*Likelihood*); *K* = number of estimated parameters; AIC*_c_* = Akaike’s Information Criterion with second-order bias correction; ΔAIC*_c_* = difference (Δ) in AIC_c_ between best approximating model and model of interest; *w* = model probability.

**Table S17.** Model evaluation of pesticide generalized linear mixed effects models on a joint index of ring-necked pheasants (*Phasianus colchicus*) abundance in California.

Model Covariate *K* *LL* ΔAIC*_c_* *w R^2^c*

HC × Pest × Year + UC × Pest × Year 16 -1645.47 0.00 0.09 0.58

HC × Pest × Year + UC × Pest × Year + Corv 17 -1644.50 0.10 0.08 0.59

HC × Pest × Year + UC × Pest × Year + PPT(Brood) 17 -1644.98 1.07 0.05 0.59

HC × Pest × Year + UC × Pest × Year + Corv + PPT(Brood) 18 -1644.04 1.24 0.05 0.59

HC × Pest × Year + UC × Pest × Year + PPT(Breed) 17 -1645.16 1.42 0.04 0.58

HC × Pest × Year + UC × Pest × Year + Corv + PPT(Breed) 18 -1644.16 1.48 0.04 0.59

HC × Pest × Year + UC × Pest × Year + TMIN(Breed) 17 -1645.22 1.55 0.04 0.58

HC × Pest × Year + UC × Pest × Year + Corv + TMIN(Breed) 18 -1644.24 1.63 0.04 0.59

HC × Pest × Year + UC × Pest × Year + TMIN(Brood) 17 -1645.26 1.64 0.04 0.59

HC × Pest × Year + UC × Pest × Year + Corv + TMIN(Brood) 18 -1644.27 1.69 0.04 0.59

HC × Pest × Year + UC × Pest × Year + Rapt 17 -1645.37 1.85 0.03 0.58

HC × Pest × Year + UC × Pest × Year + TMIN(Win) 17 -1645.45 2.01 0.03 0.59

HC × Pest × Year + UC × Pest × Year + Turk 17 -1645.46 2.02 0.03 0.58

HC × Pest × Year + UC × Pest × Year + Corv + TMIN(Win) 18 -1644.48 2.12 0.03 0.59

HC × Pest × Year + UC × Pest × Year + Corv + Rapt 18 -1644.49 2.13 0.03 0.59

HC × Pest × Year + UC × Pest × Year + Corv + Turk 18 -1644.49 2.13 0.03 0.59

HC × Pest × Year + UC × Pest × Year + TMIN(Breed) + PPT(Brood) 18 -1644.74 2.64 0.02 0.58

HC × Pest × Year + UC × Pest × Year + PPT(Brood) + PPT(Breed) 18 -1644.77 2.69 0.02 0.58

HC × Pest × Year + UC × Pest × Year + TMIN(Brood) + PPT(Brood) 18 -1644.79 2.74 0.02 0.59

HC × Pest × Year + UC × Pest × Year + Rapt + PPT(Brood) 18 -1644.89 2.93 0.02 0.59

HC × Pest × Year + UC × Pest × Year + TMIN(Brood) + PPT(Breed) 18 -1644.89 2.94 0.02 0.59

HC × Pest × Year + UC × Pest × Year + TMIN(Win) + PPT(Brood) 18 -1644.92 3.00 0.02 0.59

HC × Pest × Year + UC × Pest × Year + TMIN(Breed) + PPT(Breed) 18 -1644.94 3.03 0.02 0.58

HC × Pest × Year + UC × Pest × Year + Turk + PPT(Brood) 18 -1644.97 3.10 0.02 0.59

HC × Pest × Year + UC × Pest × Year + Rapt + PPT(Breed) 18 -1645.05 3.26 0.02 0.58

HC × Pest × Year + UC × Pest × Year + Rapt + TMIN(Breed) 18 -1645.12 3.40 0.02 0.58

HC × Pest × Year + UC × Pest × Year + Turk + PPT(Breed) 18 -1645.15 3.46 0.02 0.58

HC × Pest × Year + UC × Pest × Year + TMIN(Win) + PPT(Breed) 18 -1645.16 3.46 0.02 0.58

HC × Pest × Year + UC × Pest × Year + Rapt + TMIN(Brood) 18 -1645.17 3.48 0.02 0.59

HC × Pest × Year + UC × Pest × Year + Turk + TMIN(Breed) 18 -1645.21 3.58 0.01 0.58

HC × Pest × Year + UC × Pest × Year + Turk + TMIN(Brood) 18 -1645.25 3.65 0.01 0.59

HC × Pest × Year + UC × Pest × Year + Rapt + TMIN(Win) 18 -1645.36 3.87 0.01 0.58

HC × Pest × Year + UC × Pest × Year + Rapt + Turk 18 -1645.37 3.88 0.01 0.58

HC × Pest × Year + UC × Pest × Year + Turk + TMIN(Win) 18 -1645.44 4.04 0.01 0.59

HC × Pest × Year + Corv 13 -1657.63 18.20 0.00 0.59

HC × Pest × Year + Corv + PPT(Brood) 14 -1656.96 18.90 0.00 0.59

HC × Pest × Year 12 -1659.07 19.06 0.00 0.59

HC × Pest × Year + Corv + TMIN(Brood) 14 -1657.23 19.44 0.00 0.60

HC × Pest × Year + PPT(Brood) 13 -1658.35 19.64 0.00 0.59

HC × Pest × Year + Corv + PPT(Breed) 14 -1657.33 19.65 0.00 0.59

HC × Pest × Year + Corv + TMIN(Breed) 14 -1657.46 19.91 0.00 0.59

HC × Pest × Year + Corv + Rapt 14 -1657.58 20.15 0.00 0.59

HC × Pest × Year + Corv + TMIN(Brood) + PPT(Brood) 15 -1656.59 20.20 0.00 0.61

HC × Pest × Year + Corv + TMIN(Win) 14 -1657.61 20.20 0.00 0.59

HC × Pest × Year + Corv + Turk 14 -1657.63 20.24 0.00 0.59

HC × Pest × Year + TMIN(Brood) 13 -1658.71 20.37 0.00 0.60

HC × Pest × Year + PPT(Breed) 13 -1658.81 20.56 0.00 0.58

HC × Pest × Year + Corv + PPT(Brood) + PPT(Breed) 15 -1656.78 20.58 0.00 0.59

HC × Pest × Year + Corv + TMIN(Breed) + PPT(Brood) 15 -1656.80 20.61 0.00 0.59

HC × Pest × Year + Rapt 13 -1658.86 20.67 0.00 0.58

HC × Pest × Year + Corv + TMIN(Brood) + PPT(Breed) 15 -1656.85 20.72 0.00 0.60

HC × Pest × Year + TMIN(Breed) 13 -1658.93 20.80 0.00 0.58

HC × Pest × Year + Corv + Rapt + PPT(Brood) 15 -1656.92 20.85 0.00 0.59

HC × Pest × Year + Corv + Turk + PPT(Brood) 15 -1656.95 20.93 0.00 0.59

HC × Pest × Year + Corv + TMIN(Win) + PPT(Brood) 15 -1656.96 20.93 0.00 0.59

HC × Pest × Year + TMIN(Brood) + PPT(Brood) 14 -1658.02 21.02 0.00 0.60

HC × Pest × Year + TMIN(Win) 13 -1659.05 21.04 0.00 0.58

HC × Pest × Year + Turk 13 -1659.07 21.09 0.00 0.59

HC × Pest × Year + Rapt + PPT(Brood) 14 -1658.14 21.27 0.00 0.59

HC × Pest × Year + PPT(Brood) + PPT(Breed) 14 -1658.19 21.37 0.00 0.59

HC × Pest × Year + Corv + Rapt + TMIN(Brood) 15 -1657.18 21.39 0.00 0.60

HC × Pest × Year + TMIN(Breed) + PPT(Brood) 14 -1658.20 21.39 0.00 0.58

HC × Pest × Year + Corv + TMIN(Breed) + PPT(Breed) 15 -1657.19 21.41 0.00 0.58

HC × Pest × Year + Corv + Turk + TMIN(Brood) 15 -1657.23 21.47 0.00 0.60

HC × Pest × Year + Corv + TMIN(Win) + PPT(Breed) 15 -1657.24 21.51 0.00 0.59

HC × Pest × Year + Corv + Rapt + PPT(Breed) 15 -1657.29 21.60 0.00 0.59

HC × Pest × Year + Turk + PPT(Brood) 14 -1658.34 21.67 0.00 0.59

HC × Pest × Year + TMIN(Win) + PPT(Brood) 14 -1658.35 21.68 0.00 0.59

HC × Pest × Year + Corv + Turk + PPT(Breed) 15 -1657.33 21.68 0.00 0.59

HC × Pest × Year + TMIN(Brood) + PPT(Breed) 14 -1658.37 21.72 0.00 0.60

HC × Pest × Year + Corv + Rapt + TMIN(Breed) 15 -1657.42 21.86 0.00 0.59

HC × Pest × Year + Corv + Turk + TMIN(Breed) 15 -1657.46 21.95 0.00 0.59

HC × Pest × Year + Rapt + TMIN(Brood) 14 -1658.50 21.98 0.00 0.60

HC × Pest × Year + Corv + Rapt + TMIN(Win) 15 -1657.56 22.14 0.00 0.59

HC × Pest × Year + Rapt + PPT(Breed) 14 -1658.59 22.16 0.00 0.58

HC × Pest × Year + Corv + Rapt + Turk 15 -1657.58 22.19 0.00 0.59

HC × Pest × Year + Corv + Turk + TMIN(Win) 15 -1657.61 22.24 0.00 0.59

HC × Pest × Year + TMIN(Breed) + PPT(Breed) 14 -1658.68 22.35 0.00 0.58

HC × Pest × Year + TMIN(Win) + PPT(Breed) 14 -1658.71 22.40 0.00 0.58

HC × Pest × Year + Turk + TMIN(Brood) 14 -1658.71 22.40 0.00 0.60

HC × Pest × Year + Rapt + TMIN(Breed) 14 -1658.71 22.41 0.00 0.58

HC × Pest × Year + Turk + PPT(Breed) 14 -1658.80 22.59 0.00 0.58

HC × Pest × Year + Rapt + TMIN(Win) 14 -1658.82 22.63 0.00 0.58

HC × Pest × Year + TMIN(Brood) + PPT(Brood) + PPT(Breed) 15 -1657.81 22.64 0.00 0.60

HC × Pest × Year + Rapt + TMIN(Brood) + PPT(Brood) 15 -1657.81 22.64 0.00 0.60

HC × Pest × Year + Rapt + Turk 14 -1658.86 22.70 0.00 0.58

HC × Pest × Year + Turk + TMIN(Breed) 14 -1658.93 22.84 0.00 0.58

HC × Pest × Year + Rapt + PPT(Brood) + PPT(Breed) 15 -1657.99 23.00 0.00 0.58

HC × Pest × Year + Rapt + TMIN(Breed) + PPT(Brood) 15 -1658.00 23.02 0.00 0.58

HC × Pest × Year + Turk + TMIN(Brood) + PPT(Brood) 15 -1658.01 23.05 0.00 0.60

HC × Pest × Year + Turk + TMIN(Win) 14 -1659.05 23.08 0.00 0.58

HC × Pest × Year + TMIN(Breed) + PPT(Brood) + PPT(Breed) 15 -1658.07 23.15 0.00 0.58

HC × Pest × Year + Rapt + Turk + PPT(Brood) 15 -1658.13 23.29 0.00 0.59

HC × Pest × Year + Rapt + TMIN(Win) + PPT(Brood) 15 -1658.14 23.31 0.00 0.59

HC × Pest × Year + Rapt + TMIN(Brood) + PPT(Breed) 15 -1658.15 23.32 0.00 0.60

HC × Pest × Year + TMIN(Win) + PPT(Brood) + PPT(Breed) 15 -1658.17 23.37 0.00 0.58

HC × Pest × Year + Turk + PPT(Brood) + PPT(Breed) 15 -1658.18 23.39 0.00 0.59

HC × Pest × Year + Turk + TMIN(Breed) + PPT(Brood) 15 -1658.20 23.42 0.00 0.58

HC × Pest × Year + Turk + TMIN(Win) + PPT(Brood) 15 -1658.34 23.71 0.00 0.59

HC × Pest × Year + Turk + TMIN(Brood) + PPT(Breed) 15 -1658.37 23.76 0.00 0.60

HC × Pest × Year + Rapt + TMIN(Breed) + PPT(Breed) 15 -1658.46 23.95 0.00 0.58

HC × Pest × Year + Rapt + TMIN(Win) + PPT(Breed) 15 -1658.46 23.95 0.00 0.58

HC × Pest × Year + Rapt + Turk + TMIN(Brood) 15 -1658.50 24.01 0.00 0.60

HC × Pest × Year + Rapt + Turk + PPT(Breed) 15 -1658.58 24.17 0.00 0.58

HC × Pest × Year + Turk + TMIN(Breed) + PPT(Breed) 15 -1658.68 24.37 0.00 0.58

HC × Pest × Year + Turk + TMIN(Win) + PPT(Breed) 15 -1658.70 24.43 0.00 0.58

HC × Pest × Year + Rapt + Turk + TMIN(Breed) 15 -1658.71 24.44 0.00 0.58

HC × Pest × Year + Rapt + Turk + TMIN(Win) 15 -1658.82 24.66 0.00 0.58

UC × Pest × Year + Corv 13 -1665.34 33.64 0.00 0.61

UC × Pest × Year + Corv + TMIN(Brood) 14 -1664.54 34.05 0.00 0.61

UC × Pest × Year + Corv + PPT(Breed) 14 -1665.00 34.99 0.00 0.61

UC × Pest × Year + Corv + PPT(Brood) 14 -1665.06 35.10 0.00 0.61

UC × Pest × Year + Corv + TMIN(Brood) + PPT(Breed) 15 -1664.06 35.14 0.00 0.61

UC × Pest × Year + Corv + TMIN(Win) 14 -1665.10 35.19 0.00 0.61

UC × Pest × Year + Corv + Turk 14 -1665.25 35.49 0.00 0.61

UC × Pest × Year + Corv + TMIN(Breed) 14 -1665.25 35.49 0.00 0.61

UC × Pest × Year + Corv + TMIN(Brood) + PPT(Brood) 15 -1664.27 35.57 0.00 0.61

UC × Pest × Year + Corv + Rapt 14 -1665.29 35.57 0.00 0.61

UC × Pest × Year + Corv + Turk + TMIN(Brood) 15 -1664.42 35.85 0.00 0.61

UC × Pest × Year + Corv + Rapt + TMIN(Brood) 15 -1664.48 35.99 0.00 0.61

UC × Pest × Year 12 -1667.70 36.31 0.00 0.61

UC × Pest × Year + Corv + TMIN(Win) + PPT(Brood) 15 -1664.71 36.45 0.00 0.61

UC × Pest × Year + Corv + PPT(Brood) + PPT(Breed) 15 -1664.80 36.63 0.00 0.61

UC × Pest × Year + Corv + TMIN(Win) + PPT(Breed) 15 -1664.88 36.79 0.00 0.61

UC × Pest × Year + Corv + TMIN(Breed) + PPT(Breed) 15 -1664.93 36.88 0.00 0.61

UC × Pest × Year + Corv + Turk + PPT(Breed) 15 -1664.94 36.89 0.00 0.61

UC × Pest × Year + TMIN(Brood) 13 -1666.98 36.90 0.00 0.61

UC × Pest × Year + Corv + Rapt + PPT(Breed) 15 -1664.95 36.93 0.00 0.61

UC × Pest × Year + Corv + TMIN(Breed) + PPT(Brood) 15 -1664.97 36.97 0.00 0.61

UC × Pest × Year + Corv + Turk + PPT(Brood) 15 -1664.98 36.98 0.00 0.61

UC × Pest × Year + Corv + Rapt + PPT(Brood) 15 -1665.00 37.03 0.00 0.61

UC × Pest × Year + Corv + Turk + TMIN(Win) 15 -1665.02 37.07 0.00 0.61

UC × Pest × Year + Corv + Rapt + TMIN(Win) 15 -1665.03 37.09 0.00 0.61

UC × Pest × Year + Corv + Turk + TMIN(Breed) 15 -1665.16 37.35 0.00 0.61

UC × Pest × Year + Corv + Rapt + Turk 15 -1665.19 37.41 0.00 0.61

UC × Pest × Year + Corv + Rapt + TMIN(Breed) 15 -1665.21 37.44 0.00 0.61

UC × Pest × Year + PPT(Brood) 13 -1667.38 37.70 0.00 0.61

UC × Pest × Year + PPT(Breed) 13 -1667.38 37.71 0.00 0.61

UC × Pest × Year + TMIN(Win) 13 -1667.46 37.87 0.00 0.61

UC × Pest × Year + TMIN(Brood) + PPT(Breed) 14 -1666.54 38.07 0.00 0.61

UC × Pest × Year + Turk 13 -1667.60 38.15 0.00 0.61

UC × Pest × Year + TMIN(Breed) 13 -1667.62 38.18 0.00 0.61

UC × Pest × Year + Rapt 13 -1667.68 38.32 0.00 0.61

UC × Pest × Year + TMIN(Brood) + PPT(Brood) 14 -1666.68 38.34 0.00 0.61

UC × Pest × Year + Turk + TMIN(Brood) 14 -1666.85 38.69 0.00 0.61

UC × Pest × Year + Rapt + TMIN(Brood) 14 -1666.97 38.91 0.00 0.61

UC × Pest × Year + TMIN(Win) + PPT(Brood) 14 -1667.03 39.05 0.00 0.61

UC × Pest × Year + PPT(Brood) + PPT(Breed) 14 -1667.15 39.29 0.00 0.61

UC × Pest × Year + TMIN(Win) + PPT(Breed) 14 -1667.26 39.50 0.00 0.61

UC × Pest × Year + Turk + PPT(Brood) 14 -1667.29 39.57 0.00 0.61

UC × Pest × Year + TMIN(Breed) + PPT(Brood) 14 -1667.30 39.59 0.00 0.61

UC × Pest × Year + Turk + PPT(Breed) 14 -1667.31 39.60 0.00 0.61

UC × Pest × Year + TMIN(Breed) + PPT(Breed) 14 -1667.32 39.62 0.00 0.61

UC × Pest × Year + TMIN(Brood) + PPT(Brood) + PPT(Breed) 15 -1666.35 39.72 0.00 0.61

UC × Pest × Year + Rapt + PPT(Brood) 14 -1667.37 39.72 0.00 0.61

UC × Pest × Year + Rapt + PPT(Breed) 14 -1667.37 39.72 0.00 0.61

UC × Pest × Year + Turk + TMIN(Win) 14 -1667.37 39.73 0.00 0.61

UC × Pest × Year + Rapt + TMIN(Win) 14 -1667.45 39.89 0.00 0.61

UC × Pest × Year + Turk + TMIN(Brood) + PPT(Breed) 15 -1666.45 39.91 0.00 0.61

UC × Pest × Year + Turk + TMIN(Breed) 14 -1667.52 40.02 0.00 0.61

UC × Pest × Year + Rapt + TMIN(Brood) + PPT(Breed) 15 -1666.53 40.08 0.00 0.61

UC × Pest × Year + Turk + TMIN(Brood) + PPT(Brood) 15 -1666.57 40.16 0.00 0.61

UC × Pest × Year + Rapt + Turk 14 -1667.59 40.17 0.00 0.61

UC × Pest × Year + Rapt + TMIN(Breed) 14 -1667.61 40.20 0.00 0.61

UC × Pest × Year + Rapt + TMIN(Brood) + PPT(Brood) 15 -1666.67 40.36 0.00 0.61

UC × Pest × Year + Rapt + Turk + TMIN(Brood) 15 -1666.85 40.71 0.00 0.61

UC × Pest × Year + TMIN(Win) + PPT(Brood) + PPT(Breed) 15 -1666.94 40.89 0.00 0.61

UC × Pest × Year + Turk + TMIN(Win) + PPT(Brood) 15 -1666.96 40.94 0.00 0.61

UC × Pest × Year + Rapt + TMIN(Win) + PPT(Brood) 15 -1667.03 41.08 0.00 0.61

UC × Pest × Year + Turk + PPT(Brood) + PPT(Breed) 15 -1667.09 41.19 0.00 0.61

UC × Pest × Year + TMIN(Breed) + PPT(Brood) + PPT(Breed) 15 -1667.09 41.21 0.00 0.61

UC × Pest × Year + Rapt + PPT(Brood) + PPT(Breed) 15 -1667.14 41.31 0.00 0.61

UC × Pest × Year + Turk + TMIN(Win) + PPT(Breed) 15 -1667.19 41.40 0.00 0.61

UC × Pest × Year + Turk + TMIN(Breed) + PPT(Brood) 15 -1667.22 41.46 0.00 0.61

UC × Pest × Year + Turk + TMIN(Breed) + PPT(Breed) 15 -1667.24 41.51 0.00 0.61

UC × Pest × Year + Rapt + TMIN(Win) + PPT(Breed) 15 -1667.25 41.53 0.00 0.61

UC × Pest × Year + Rapt + Turk + PPT(Brood) 15 -1667.29 41.60 0.00 0.61

UC × Pest × Year + Rapt + TMIN(Breed) + PPT(Brood) 15 -1667.29 41.61 0.00 0.61

UC × Pest × Year + Rapt + Turk + PPT(Breed) 15 -1667.30 41.62 0.00 0.61

UC × Pest × Year + Rapt + TMIN(Breed) + PPT(Breed) 15 -1667.31 41.63 0.00 0.61

UC × Pest × Year + Rapt + Turk + TMIN(Win) 15 -1667.37 41.76 0.00 0.61

UC × Pest × Year + Rapt + Turk + TMIN(Breed) 15 -1667.51 42.05 0.00 0.61

Corv + PPT(Brood) 7 -1716.51 123.80 0.00 0.62

Corv + Turk + PPT(Brood) 8 -1715.60 124.02 0.00 0.62

PPT(Brood) 6 -1717.64 124.04 0.00 0.62

Turk + PPT(Brood) 7 -1716.80 124.38 0.00 0.62

Corv 6 -1718.08 124.94 0.00 0.62

Corv + Turk 7 -1717.20 125.18 0.00 0.62

Survey type 5 -1719.24 125.23 0.00 0.62

Corv + Rapt + PPT(Brood) 8 -1716.25 125.32 0.00 0.62

Corv + TMIN(Breed) + PPT(Brood) 8 -1716.34 125.49 0.00 0.62

Corv + TMIN(Brood) + PPT(Brood) 8 -1716.38 125.57 0.00 0.62

Turk 6 -1718.41 125.60 0.00 0.62

Corv + PPT(Brood) + PPT(Breed) 8 -1716.43 125.66 0.00 0.62

Corv + Rapt + Turk + PPT(Brood) 9 -1715.42 125.67 0.00 0.62

Corv + Turk + TMIN(Breed) + PPT(Brood) 9 -1715.45 125.73 0.00 0.62

TMIN(Breed) + PPT(Brood) 7 -1717.48 125.75 0.00 0.62

Corv + TMIN(Win) + PPT(Brood) 8 -1716.47 125.76 0.00 0.62

Corv + Turk + PPT(Brood) + PPT(Breed) 9 -1715.48 125.78 0.00 0.62

Corv + Turk + TMIN(Brood) + PPT(Brood) 9 -1715.48 125.80 0.00 0.62

TMIN(Brood) + PPT(Brood) 7 -1717.52 125.83 0.00 0.62

Rapt + PPT(Brood) 7 -1717.56 125.92 0.00 0.62

PPT(Brood) + PPT(Breed) 7 -1717.57 125.92 0.00 0.62

Corv + Turk + TMIN(Win) + PPT(Brood) 9 -1715.55 125.94 0.00 0.62

TMIN(Win) + PPT(Brood) 7 -1717.60 126.00 0.00 0.62

Turk + TMIN(Breed) + PPT(Brood) 8 -1716.65 126.11 0.00 0.62

Turk + PPT(Brood) + PPT(Breed) 8 -1716.68 126.18 0.00 0.62

Turk + TMIN(Brood) + PPT(Brood) 8 -1716.69 126.18 0.00 0.62

Turk + TMIN(Win) + PPT(Brood) 8 -1716.75 126.30 0.00 0.62

Rapt + Turk + PPT(Brood) 8 -1716.76 126.33 0.00 0.62

Corv + Rapt 7 -1717.84 126.46 0.00 0.62

Corv + PPT(Breed) 7 -1717.85 126.48 0.00 0.62

Corv + Turk + PPT(Breed) 8 -1716.89 126.58 0.00 0.62

Corv + TMIN(Breed) 7 -1717.90 126.58 0.00 0.62

Corv + TMIN(Brood) 7 -1717.95 126.69 0.00 0.62

PPT(Breed) 6 -1719.02 126.81 0.00 0.62

Corv + Rapt + Turk 8 -1717.01 126.84 0.00 0.62

Corv + Turk + TMIN(Breed) 8 -1717.02 126.86 0.00 0.62

TMIN(Breed) 6 -1719.06 126.89 0.00 0.63

Corv + Turk + TMIN(Brood) 8 -1717.07 126.94 0.00 0.62

Corv + TMIN(Win) 7 -1718.08 126.95 0.00 0.62

TMIN(Brood) 6 -1719.11 127.00 0.00 0.62

Corv + Rapt + TMIN(Breed) + PPT(Brood) 9 -1716.09 127.01 0.00 0.62

Turk + PPT(Breed) 7 -1718.13 127.04 0.00 0.62

Corv + Rapt + TMIN(Brood) + PPT(Brood) 9 -1716.13 127.09 0.00 0.62

Rapt 6 -1719.17 127.11 0.00 0.62

Corv + Rapt + PPT(Brood) + PPT(Breed) 9 -1716.17 127.18 0.00 0.62

Corv + Turk + TMIN(Win) 8 -1717.20 127.20 0.00 0.62

Corv + Rapt + TMIN(Win) + PPT(Brood) 9 -1716.20 127.23 0.00 0.62

TMIN(Win) 6 -1719.23 127.24 0.00 0.62

Turk + TMIN(Breed) 7 -1718.25 127.29 0.00 0.62

Corv + TMIN(Breed) + PPT(Brood) + PPT(Breed) 9 -1716.27 127.37 0.00 0.62

Turk + TMIN(Brood) 7 -1718.29 127.38 0.00 0.62

Corv + TMIN(Brood) + PPT(Brood) + PPT(Breed) 9 -1716.27 127.38 0.00 0.62

Rapt + Turk 7 -1718.38 127.55 0.00 0.62

Turk + TMIN(Win) 7 -1718.41 127.62 0.00 0.62

Rapt + TMIN(Breed) + PPT(Brood) 8 -1717.41 127.63 0.00 0.62

TMIN(Breed) + PPT(Brood) + PPT(Breed) 8 -1717.42 127.65 0.00 0.62

TMIN(Brood) + PPT(Brood) + PPT(Breed) 8 -1717.43 127.66 0.00 0.62

Corv + TMIN(Win) + PPT(Brood) + PPT(Breed) 9 -1716.42 127.66 0.00 0.62

Rapt + TMIN(Brood) + PPT(Brood) 8 -1717.45 127.71 0.00 0.62

Rapt + PPT(Brood) + PPT(Breed) 8 -1717.49 127.80 0.00 0.62

Rapt + TMIN(Win) + PPT(Brood) 8 -1717.52 127.85 0.00 0.62

TMIN(Win) + PPT(Brood) + PPT(Breed) 8 -1717.56 127.92 0.00 0.62

Turk + TMIN(Brood) + PPT(Brood) + PPT(Breed) 9 -1716.54 127.92 0.00 0.62

Turk + TMIN(Breed) + PPT(Brood) + PPT(Breed) 9 -1716.55 127.94 0.00 0.62

Corv + Rapt + PPT(Breed) 8 -1717.60 128.00 0.00 0.62

Rapt + Turk + TMIN(Breed) + PPT(Brood) 9 -1716.62 128.06 0.00 0.62

Corv + Rapt + TMIN(Breed) 8 -1717.65 128.11 0.00 0.62

Rapt + Turk + PPT(Brood) + PPT(Breed) 9 -1716.65 128.13 0.00 0.62

Rapt + Turk + TMIN(Brood) + PPT(Brood) 9 -1716.65 128.13 0.00 0.62

Corv + TMIN(Brood) + PPT(Breed) 8 -1717.67 128.14 0.00 0.62

Turk + TMIN(Win) + PPT(Brood) + PPT(Breed) 9 -1716.67 128.17 0.00 0.62

Corv + TMIN(Breed) + PPT(Breed) 8 -1717.68 128.17 0.00 0.63

Corv + Rapt + TMIN(Brood) 8 -1717.70 128.21 0.00 0.62

Rapt + Turk + TMIN(Win) + PPT(Brood) 9 -1716.70 128.23 0.00 0.62

Corv + Rapt + Turk + PPT(Breed) 9 -1716.70 128.24 0.00 0.62

Corv + Turk + TMIN(Brood) + PPT(Breed) 9 -1716.70 128.24 0.00 0.62

Corv + Turk + TMIN(Breed) + PPT(Breed) 9 -1716.74 128.31 0.00 0.62

Corv + TMIN(Win) + PPT(Breed) 8 -1717.81 128.44 0.00 0.62

Corv + Rapt + TMIN(Win) 8 -1717.84 128.48 0.00 0.62

TMIN(Brood) + PPT(Breed) 7 -1718.85 128.49 0.00 0.63

TMIN(Breed) + PPT(Breed) 7 -1718.86 128.51 0.00 0.63

Corv + Rapt + Turk + TMIN(Breed) 9 -1716.84 128.52 0.00 0.62

Corv + Turk + TMIN(Win) + PPT(Breed) 9 -1716.86 128.55 0.00 0.62

Corv + Rapt + Turk + TMIN(Brood) 9 -1716.88 128.60 0.00 0.62

Rapt + PPT(Breed) 7 -1718.95 128.69 0.00 0.63

Turk + TMIN(Brood) + PPT(Breed) 8 -1717.96 128.73 0.00 0.62

TMIN(Win) + PPT(Breed) 7 -1718.98 128.76 0.00 0.62

Rapt + TMIN(Breed) 7 -1718.99 128.78 0.00 0.63

Turk + TMIN(Breed) + PPT(Breed) 8 -1717.99 128.78 0.00 0.63

Corv + Rapt + Turk + TMIN(Win) 9 -1717.01 128.86 0.00 0.62

Rapt + TMIN(Brood) 7 -1719.04 128.88 0.00 0.63

Rapt + Turk + PPT(Breed) 8 -1718.09 128.99 0.00 0.62

Turk + TMIN(Win) + PPT(Breed) 8 -1718.10 129.01 0.00 0.62

Rapt + TMIN(Win) 7 -1719.17 129.13 0.00 0.62

Rapt + Turk + TMIN(Breed) 8 -1718.22 129.24 0.00 0.62

Rapt + Turk + TMIN(Brood) 8 -1718.26 129.33 0.00 0.62

Rapt + TMIN(Breed) + PPT(Brood) + PPT(Breed) 9 -1717.35 129.53 0.00 0.62

Rapt + TMIN(Brood) + PPT(Brood) + PPT(Breed) 9 -1717.35 129.54 0.00 0.62

Rapt + Turk + TMIN(Win) 8 -1718.38 129.57 0.00 0.62

Corv + Rapt + TMIN(Brood) + PPT(Breed) 9 -1717.41 129.66 0.00 0.62

Corv + Rapt + TMIN(Breed) + PPT(Breed) 9 -1717.44 129.70 0.00 0.63

Rapt + TMIN(Win) + PPT(Brood) + PPT(Breed) 9 -1717.48 129.79 0.00 0.62

Random Effects Only 3 -1723.58 129.90 0.00 0.62

Corv + Rapt + TMIN(Win) + PPT(Breed) 9 -1717.58 129.99 0.00 0.62

Rapt + TMIN(Brood) + PPT(Breed) 8 -1718.78 130.37 0.00 0.63

Rapt + TMIN(Breed) + PPT(Breed) 8 -1718.79 130.40 0.00 0.63

Rapt + TMIN(Win) + PPT(Breed) 8 -1718.92 130.66 0.00 0.62

Rapt + Turk + TMIN(Brood) + PPT(Breed) 9 -1717.92 130.68 0.00 0.62

Rapt + Turk + TMIN(Breed) + PPT(Breed) 9 -1717.95 130.74 0.00 0.63

Rapt + Turk + TMIN(Win) + PPT(Breed) 9 -1718.07 130.97 0.00 0.62

^a^ These models were carried forward to the second step of the modeling process.

Column Abbreviations: *LL* = Log(*Likelihood*); *K* = number of estimated parameters; AIC*_c_* = Akaike’s Information Criterion with second-order bias correction; ΔAIC*_c_* = difference (Δ) in AIC_c_ between best approximating model and model of interest; *w* = model probability.

**Table S18.** Model evaluation of post hoc crop type analysis using generalized linear mixed effects models on ring-necked pheasant (*Phasianus colchicus*) abundance in California.

Model Covariate *K* *LL* ΔAIC*_c_* *w*

Barley 6 -2095.38 0.00 1.00

Sugar beets 6 -2119.71 48.66 0.00

Nut trees 6 -2123.78 56.79 0.00

Winter wheat 6 -2141.47 92.17 0.00

Sorghum 6 -2175.37 159.97 0.00

Vegetable seed 6 -2184.50 178.23 0.00

Cotton 6 -2194.10 197.43 0.00

Grapes 6 -2194.52 198.26 0.00

Rice 6 -2199.94 209.10 0.00

Corn 6 -2202.66 214.54 0.00

Oats 6 -2203.37 215.97 0.00

Hay 6 -2203.38 215.99 0.00

Baseline Model 5 -2204.71 216.65 0.00

Wheat 6 -2204.05 217.33 0.00

Random Effects Only 3 -2207.20 217.59 0.00

Fruit trees 6 -2204.71 218.64 0.00

Column Abbreviations: *LL* = Log(*Likelihood*); *K* = number of estimated parameters; AIC*_c_* = Akaike’s Information Criterion with second-order bias correction; ΔAIC*_c_* = difference (Δ) in AIC_c_ between best approximating model and model of interest; *w* = model probability.

**Supporting Information, Figure Legends**

**Fig. S1.** Land use practices (millions of acres) taking place in California, USA from 1945–2012. Solid line represents LOESS curve.

**Fig. S2.** Acres planted (millions) of harvested cropland (solid line), cropland used as pasture (long dashed line), unharvested cropland (dot-dashed line) and CRP (dotted line) in A) Northern, B) North Central, C) Bay Delta, D) Central, E) South Coast and F) Inland Deserts regions in California, 1949-2007 (USDA Agricultural Census data).

**Fig. S3.** Statewide average Breeding Bird Survey counts for A) corvids, B) raptors and C) turkeys in California, USA from 1948–2012. Solid line represents LOESS curve.

**Fig. S4.** Statewide climate trends showing A) minimum temperature (°C) during the breeding season (April – July), B) minimum temperature (°C) during the brood-rearing season (August – November), C) minimum temperature (°C) during the winter season (December – March), D) precipitation during the breeding season, E) precipitation during the brood-rearing season and F) precipitation during the winter season in California 1913-2013. Solid line represents LOESS curve.

**Fig. S5.** Acres planted (millions) of A) barley, B) sugar beets, C) sorghum, D) nut trees, E) rice and F) winter wheat in California, USA from 1945–2013. Solid line represents LOESS curve.

**Fig. S6**. Acres of (A) rice planted in the North Central region of California, USA from 1953–2013 and (B) nut trees planted in the Central region of California, USA from 1980–2013. Solid line represents LOESS curve.
